# Optic Atrophy 1 Is Epistatic to the Core MICOS Component MIC60 in Mitochondrial Cristae Shape Control

**DOI:** 10.1016/j.celrep.2016.11.049

**Published:** 2016-12-13

**Authors:** Christina Glytsou, Enrique Calvo, Sara Cogliati, Arpit Mehrotra, Irene Anastasia, Giovanni Rigoni, Andrea Raimondi, Norihito Shintani, Marta Loureiro, Jesùs Vazquez, Luca Pellegrini, Jose Antonio Enriquez, Luca Scorrano, Maria Eugenia Soriano

**Affiliations:** 1 Department of Biology, University of Padova, Padova 35121, Italy; 2 IRCCS Fondazione Santa Lucia, Rome 00179, Italy; 3 Dulbecco-Telethon Institute, Venetian Institute of Molecular Medicine, Padova 35129, Italy; 4 Centro Nacional de Investigaciones Cardiovasculares Carlos III, Madrid 28029, Spain; 5 Centro Imaging Sperimentale, IRCCS Istituto Scientifico San Raffaele, Milano 20132, Italy; 6 Graduate School of Pharmaceutical Sciences, Osaka University, Osaka 565-0871, Japan; 7 Department of Molecular Biology, Medical Biochemistry and Pathology, Université Laval, Quebec G1J 2G3, Canada

**Keywords:** mitochondria, cristae, MICOS, OPA1, proteomics

## Abstract

The mitochondrial contact site and cristae organizing system (MICOS) and Optic atrophy 1 (OPA1) control cristae shape, thus affecting mitochondrial function and apoptosis. Whether and how they physically and functionally interact is unclear. Here, we provide evidence that OPA1 is epistatic to MICOS in the regulation of cristae shape. Proteomic analysis identifies multiple MICOS components in native OPA1-containing high molecular weight complexes disrupted during cristae remodeling. MIC60, a core MICOS protein, physically interacts with OPA1, and together, they control cristae junction number and stability, OPA1 being epistatic to MIC60. OPA1 defines cristae width and junction diameter independently of MIC60. Our combination of proteomics, biochemistry, genetics, and electron tomography provides a unifying model for mammalian cristae biogenesis by OPA1 and MICOS.

## Highlights

• Complexes containing OPA1 and MIC60 are targeted during cristae remodeling
• OPA1 lies upstream of MIC60 in regulating cristae junction number and stability
• OPA1 is the sole regulator of cristae junction width

## Graphical Abstract

**Figure:**
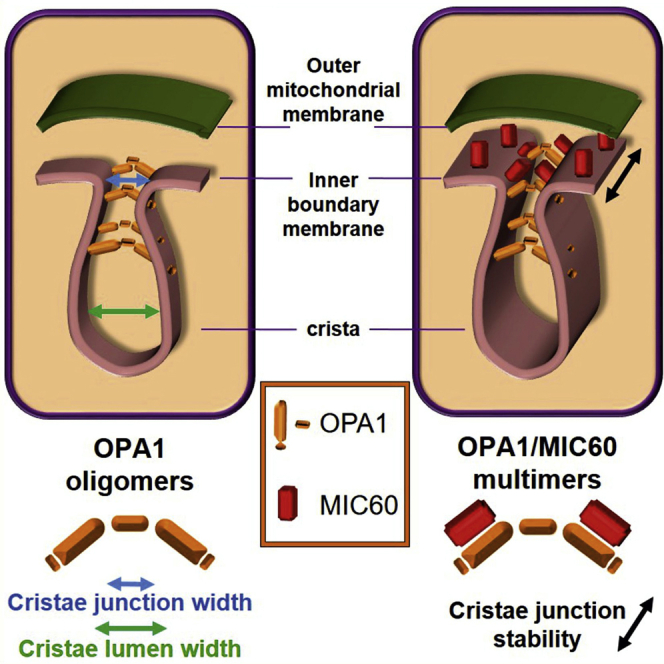

## Teaser

Mitochondrial ultrastructure dictates mitochondrial function and cell viability. Glytsou et al. find that Optic Atrophy 1 (OPA1) and the MICOS component MIC60 interact in cristae shape regulation and place OPA1 epistatic to MIC60 in cristae biogenesis and remodeling, providing a unifying molecular model of cristae morphology control.

## Introduction

The pleotropic roles of mitochondria in energy conversion, cell death, calcium homeostasis, intermediary metabolism, cell differentiation, and even immunity are matched by their morphological and ultrastructural complexity (Pernas and Scorrano, 2016). Mitochondria are organized into five sub-compartments: outer membrane (OMM), intermembrane space (IMS), inner boundary membrane (IBM), cristae, and matrix (Cogliati et al., 2016). This organization allows sub-compartmentalization of biochemical processes. For instance, cristae host the oxidative phosphorylation components, including cytochrome *c*, an essential cofactor of effector caspases activation during apoptosis (Pernas and Scorrano, 2016), and allow their assembly into respiration-competent complexes (RCCs) and respiration-competent super-complexes (RCSs) (Acin-Perez and Enriquez, 2014, Acín-Pérez et al., 2008, Lapuente-Brun et al., 2013). Therefore, cristae dictate organelle bioenergetic capacity (Hackenbrock, 1966), a concept validated in vivo using mouse genetic models (Cogliati et al., 2013, Varanita et al., 2015), and participate in apoptosis by controlling the complete cytochrome *c* release (Scorrano et al., 2002).

Functional and electron tomography (ET) studies revealed a high degree of structural complexity of the cristae sub-compartment at the level of both the cristae lumen (CL, the space inside the cristae invagination) and the cristae junction (CJ, the opening of the cristae into the IBM) (Frey and Mannella, 2000). Width is a critical functional parameter of both CL and CJ: the cristae lumen width (CLW), i.e., the distance between the opposing faces of the cristae membrane, controls RCSs assembly and mitochondrial respiratory efficiency (Cogliati et al., 2013). The cristae junction width (CJW) measures 20–40 nm and regulates metabolite diffusion and respiratory chain component segregation between the inner and the cristae membranes (Gilkerson et al., 2003). In addition, CJW increases during apoptosis, allowing cytochrome *c* redistribution from the lumen to the IMS (Große et al., 2016, Scorrano et al., 2002, Yamaguchi et al., 2008). Both CLW and CJW are controlled by the dynamin guanosine triphosphatase (GTPase) OPA1 (Cogliati et al., 2013, Frezza et al., 2006), independent of its role in mitochondrial fusion (Cipolat et al., 2004, Frezza et al., 2006). The direct relationship among CJW, CLW, and OPA1 affects cell death in vitro (Corrado et al., 2016, Costa et al., 2010, Jiang et al., 2014, Landes et al., 2010, Yamaguchi et al., 2008) and in vivo (Civiletto et al., 2015, Varanita et al., 2015). Molecularly, increased apoptotic CLW and CJW correlates with destabilization of ∼720 kDa OPA1-containing complexes of unknown composition (Cogliati et al., 2013, Frezza et al., 2006, Varanita et al., 2015).

In addition to OPA1, a large complex, the mitochondrial contact site and cristae organizing system (MICOS) regulates CJ biogenesis in multiple organisms (Herrmann, 2011, Huynen et al., 2016, Jans et al., 2013, John et al., 2005, van der Laan et al., 2016, Zerbes et al., 2012). In yeast, MICOS can be subdivided into a sub-complex formed by Mic27, Mic10, and Mic12 and a second one comprising Mic60 (mitofilin) and Mic19 (CHCHD3). Mic19 connects the two sub-complexes (Friedman et al., 2015). This picture is less clear in mammals: first, a homolog of Mic12 has not been yet identified; second, the existence of these two sub-complexes remains unexplored (Huynen et al., 2016); third, silencing of the mammalian homolog of yeast Mic10 does not alter cristae shape, whereas in yeast, its deletion results in a thylakoid-like cristae morphology (Alkhaja et al., 2012, Barbot et al., 2015, Bohnert et al., 2015). Thus, MICOS complex composition and regulation may have changed during vertebrate evolution, perhaps to accommodate its recruitment into the process of apoptotic cristae remodeling. The intriguing possibility that MIC60 and more generally the MICOS complex interact with OPA1 in CLW and CJW regulation has not been substantiated by the retrieval of OPA1 or of its yeast homolog Mgm1p in the MICOS complex so far.

Here we set out to investigate whether MICOS and OPA1 interact in cristae biogenesis and remodeling. A combination of proteomics and biochemistry indicates that mammalian MIC60 interacts with OPA1 in high molecular weight complexes (HMWCs) eliminated during cristae remodeling. An epistasis analysis places OPA1 upstream of MIC60 in the same pathway controlling CJ stability; conversely, OPA1 defines CLW and CJW independently of MIC60. Accordingly, MIC60 does not regulate apoptotic cristae remodeling and cytochrome *c* redistribution. Our results provide a unifying model for mammalian cristae biogenesis and apoptotic remodeling.

## Results

### MIC60 and OPA1 Are Retrieved in the Same Complexes Targeted during Cristae Remodeling

**Figure 1:**
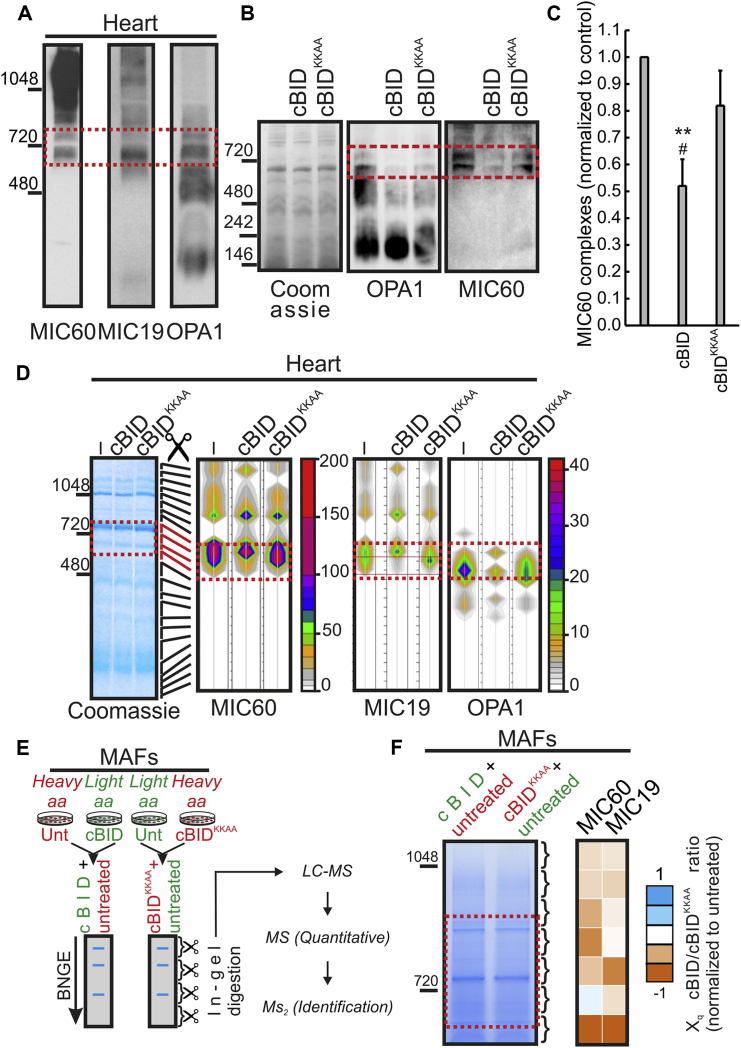
MIC60-Containing Complexes Are Disrupted during Cristae Remodeling (A) Complexes extracted from mouse heart mitochondria were separated by blue native (BN)-PAGE, transferred onto a PVDF membrane, and probed with the indicated antibodies. Boxed area: common OPA1 and MIC60 complexes. (B) Protein complexes extracted from mouse heart mitochondria treated as indicated were separated by BNGE, Coomassie stained or transferred onto PVDF membranes, and probed using the indicated antibodies. (C) Quantitative densitometric analysis of MIC60 complex levels in experiments as in (B). Data represent mean ± SEM of three independent experiments. ^∗∗^p < 0.01 in a paired sample Student’s t test between cBID and untreated; #p < 0.05 between cBID and cBID^KKAA^. (D) Mass spectrometry analysis of MIC60, MIC19, and OPA1 in HMWCs. Heart mitochondria were treated for 20 min as indicated, and extracted complexes were separated by BNGE and Coomassie stained (left). The indicated gel bands were sliced, processed, and analyzed by MS. Color contour plots (right) indicate the number of spectral counts corresponding to MIC60, MIC19, or OPA1 peptides along the BNGE. (E) Schematic representation of the SILAC experiments. One experiment (left) contains complexes extracted from control (Unt) and cBID-treated mitochondria isolated from mouse adult fibroblasts (MAFs) grown in a medium containing heavy and light amino acids, respectively. A second experiment (right) contains complexes extracted from control (Unt, light) and cBID^KKAA^-treated (heavy) mitochondria. Mixed mitochondrial complexes were separated in native conditions, and the obtained gel bands from 500 to 1,048 kDa were excised. A pool of triplicates for each experiment was analyzed by qualitative and quantitative MS. (F) Heatmap of the ratios of MIC60 peptides in cBID-treated versus cBID^KKAA^-treated samples from three independent SILAC experiments as in (E). Orange, decrease; white, no change; blue, increase.

Cytochrome *c* is a key activator of programmed cell death in vertebrates, possibly explaining the recruitment of cristae remodeling and CJ proteins like OPA1 in this process at the onset of vertebrate evolution. Yeast and mammalian MIC60 form high molecular weight complexes (HMWCs) (Guarani et al., 2015, Li et al., 2016), but whether and how its core component MIC60, conserved in all ancestral mitochondrial precursors from which cristae are retrieved (Muñoz-Gómez et al., 2015a), changes during physiopathological processes remains unknown. We therefore decided to address whether mammalian MIC60 HMWCs change during apoptotic cristae remodeling, when OPA1-containing HMWCs (∼720 kDa) are disassembled (Cogliati et al., 2013, Varanita et al., 2015). Blue native gel electrophoresis (BNGE) confirmed that in isolated heart mitochondria, MIC60 and MIC19, the other crucial MICOS component interacting with MIC60 to regulate CJ biogenesis (Darshi et al., 2011, Li et al., 2016, Xie et al., 2007), are retrieved in 600–1,000 kDa HMWCs that partially overlap with the OPA1-containing HMWCs (dashed box in Figure 1A). We next induced apoptotic changes to mitochondria by incubating them with the recombinant caspase-8 cleaved BH3-only BCL-2 family member BID (cBID) or with its cristae remodeling incompetent mutant cBID^KKAA^ (Cogliati et al., 2013). Not only the ∼720 kDa OPA1 but also the MIC60-containing complexes running at the same apparent molecular weight (MW) were selectively destabilized during cristae remodeling (dashed box in Figure 1B and quantification in Figure 1C).

To further characterize the fate of the MIC60/MIC19-containing complexes changed during apoptotic-cristae remodeling, we excised and processed for mass spectrometry (MS) analysis protein complexes separated by BNGE from cBID- and cBID^KKAA^-treated mouse heart mitochondria. A color contour plot representation of the number of spectral counts from MIC60 peptides identified by semiquantitative proteomic analysis in the different HMWCs isolated from normal and apoptotic mitochondria indicated that cBID, but not cBID^KKAA^, greatly reduced the number of MIC60/MIC19 peptides across the ∼720 kDa HMWCs. In these HMWCs, OPA1 levels were also reduced (dashed boxes in Figure 1D). Finally, a quantitative proteomic analysis of mitochondrial complexes isolated after stable isotope labeling with amino acids in cell culture (SILAC) of mouse adult fibroblasts (MAFs) (Figure 1E) confirmed that cBID, but not cBID^KKAA^, caused a significant loss of a comprehensive set of MIC60/MIC19 peptides in the ∼720 kDa MIC60/MIC19-containing HMWCs (Figure 1F). In conclusion, the two critical mammalian MICOS components, MIC60 and MIC19, assemble in HMWCs disrupted during apoptotic cristae remodeling.

### Yeast and Mammalian MIC60 Diverge

Because OPA1 and MICOS components are retrieved in the same HMWC targeted during cristae remodeling, we reasoned that OPA1 and MIC60 could physically interact. However, because in yeast the interaction between the OPA1 homolog Mgm1p and the MIC60 homolog Fcj1 does not occur, divergent OPA1 and MIC60 structural features are present in vertebrates to support their physical interaction. A bioinformatic analysis indicated that the transmembrane helices (TMHs) of mammalian and yeast MIC60 are highly divergent (Figures S1A–S1C). This finding is not unexpected, because TMHs are typically poorly conserved; however, whereas the TMH of yeast MIC60 can form a highly structured hydrophobic α helix, that of mammalian MIC60 is predicted to be poorly structured and hydrophobic, mostly because it contains two clusters of glycine residues (Figure S1B) that could facilitate the mobility and dynamic MIC60 interaction with a variety of proteins. Furthermore, MIC60 TMH is strictly conserved in vertebrates, indicating that it was subjected to structural constraints not only linked to its membrane-anchoring function. We also found motifs strictly conserved in all vertebrates absent in yeast Mic60 (Figure S1C): these sequences span the middle mammalian MIC60 and do not display obvious homology with any other mouse and human sequence, suggesting again strong purifying selection during vertebrate evolution. In conclusion, whereas the C-terminal domain is conserved in all eukaryotic orthologs of MIC60 (Muñoz-Gómez et al., 2015a, Muñoz-Gómez et al., 2015b), novel TMH and N-terminal domains emerged at the outset of vertebrate evolution, possibly to recruit MIC60 into a pathway-like apoptosis or to confer new partners and mechanisms of regulation.

### OPA1 and MIC60 Interact Physically

**Figure 2:**
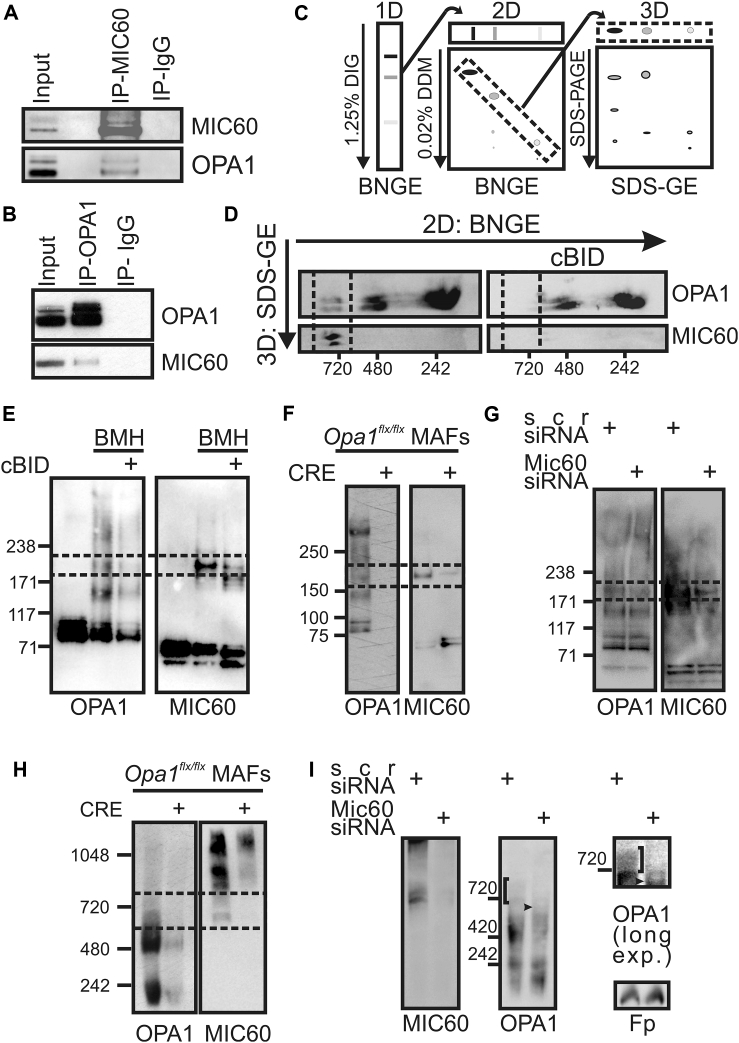
MIC60 and OPA1 Interact in Complexes Disrupted during Cristae Remodeling (A) Liver mitochondrial lysates (250 μg) were immunoprecipitated with anti-MIC60 coupled to Protein A agarose beads. Bound proteins were separated by SDS-GE and immunoblotted using the indicated antibodies. Input was diluted 1:10. (B) Mitochondrial lysates from mouse embryonic fibroblast (MEF; 125 μg) were immunoprecipitated with anti-OPA1 coupled to Protein A agarose beads. Bound proteins were separated by SDS-GE and immunoblotted using the indicated antibodies. Input was diluted 1:5. (C) Schematic representation of 3D BN-BN-SDS-GE analysis of membrane complexes isolated from mouse heart mitochondria. (D) Western blots using the indicated antibodies of 3D SDS-GE of mouse heart mitochondria treated as indicated. (E) Equal amounts (25 μg) of protein from MAF mitochondria treated as indicated and crosslinked where indicated (BMH) were separated by SDS-GE and immunoblotted using the indicated antibodies. Boxed area: OPA1-MIC60 adduct. (F) Mitochondria isolated from *Opa1*^flx/flx^ MAFs infected for 48 hr as indicated were crosslinked with 5 mM BMH. Equal amounts (25 μg) of proteins were separated by SDS-GE and immunoblotted with the indicated antibodies. Boxed area: OPA1-MIC60 adduct. See also Figure S2C. (G) Equal amounts (25 μg) of mitochondrial protein from MAFs transfected with scramble (scr) or Mic60 siRNA (3) were crosslinked with 5 mM BMH, separated by SDS-GE, and immunoblotted using the indicated antibodies. Boxed area: OPA1-MIC60 adduct. See also Figure S2D. (H) Protein complexes extracted from mitochondria (150 μg) isolated from *Opa1*^flx/flx^ MAFs infected for 48 hr as indicated were separated by BNGE and immunoblotted using the indicated antibodies. Boxed area: common OPA1 and MIC60 complexes. (I) Protein complexes extracted from mitochondria (150 μg) isolated from MAFs transfected with scramble (scr) or Mic60 siRNA (3) were separated by BNGE and immunoblotted using the indicated antibodies. Square brackets: OPA1 complex destabilized after *Mic60* silencing. Arrows: lower HMWC containing OPA1. DIG, digitonin; DDM, n-dodecyl β-D-maltoside; IP, immunoprecipitation; IP-IgG, beads coupled to irrelevant immunoglobulin G (IgG).

Comforted by the results of the bioinformatics analysis, we decided to verify in the wet lab the possibility of an OPA1-MIC60 interaction. First, we found that a fraction of OPA1 co-immunoprecipitated with MIC60 (Figure 2A) and reciprocally that MIC60 co-immunoprecipitated with OPA1 (Figure 2B). To address whether this interaction also occurred in native complexes, we turned to three-dimensional (3D) blue native-blue native-SDS gel electrophoresis (BN-BN-SDS-GE) that allows the identification of proteins coexisting in detergent-resistant complexes (Figure 2C). Immunoblotting of 3D SDS gel electrophoresis (SDS-GE) showed that MIC60 was retrieved in the ∼720 kDa OPA1-containing complex targeted during cristae remodeling (Figure 2D). Moreover, the long-arm, homo-bifunctional, maleimide crosslinker bismaleimidohexane (BMH) stabilized a ∼180–190 kDa complex immunoreactive for both OPA1 (MW ≈ 90–100 kDa) and MIC60 (MW ≈ 90 kDa) that was also reduced in apoptotic cBID-treated mitochondria (Figure 2E). This OPA1- and MIC60-immunoreactive adduct was also observed when we crosslinked proteins using the primary amine’s long-arm cleavable crosslinker dithiobis(succinimidyl propionate) (DSP) (Figure S2A), suggesting that its formation does not depend on the crosslinker used. Finally, the amount of OPA1 co-immunoprecipitated with MIC60 was greater in these DSP-treated mitochondria (Figure S2B), substantiating that crosslinking stabilizes a specific MIC60-OPA1 interaction. To verify that the crosslinked adduct was specifically formed by OPA1 and MIC60, we inspected its stability following deletion of either OPA1 or MIC60. Upon adenoviral CRE recombinase delivery to *Opa1*^flx/flx^ mouse adult fibroblasts (MAFs) (Cogliati et al., 2013), *Opa1* was successfully deleted (Figure S2C) and MIC60 was no longer retrieved in the crosslinked ∼180–190 kDa complex (Figure 2F). Similarly, *Mic60* silencing using three different short interfering RNA (siRNA) decreased MIC60 levels (Figure S2D), as well as the amount of OPA1 found in the ∼180–190 kDa crosslinked form (Figure 2G). These experiments confirm that the MIC60/OPA1 crosslinked adduct is specific and depends on both OPA1 and MIC60.

We next analyzed whether also the MIC60-OPA1 native ∼720 kDa complexes were affected by *Opa1* and *Mic60* ablation. After acute *Opa1* ablation, MIC60 was no longer retrieved in ∼720 kDa complexes (Figure 2H). Similarly, in *Mic60*-silenced mitochondria, the ∼720 kDa complex was no longer immunoreactive for OPA1 and a new OPA1-positive complex ∼600–650 kDa appeared (Figure 2I). In conclusion, these experiments indicate that OPA1 physically interacts with MIC60 and recruits it in a ∼720 kDa HMWC: when *Opa1* is deleted, MIC60 is no longer retrieved in this complex, and when MIC60 is silenced, OPA1 is found in a new ∼600–650 kDa HMWC.

### The Effect of MIC60 on Mammalian Cristae Junctions Requires OPA1

**Figure 3:**
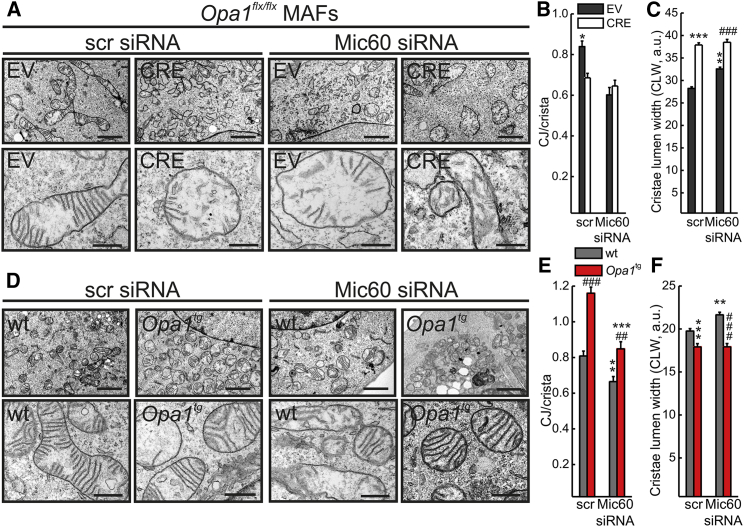
MIC60 and OPA1 Lie in the Same Genetic Pathway Controlling the CJ Number (A) Representative EM of *Opa1*^flx/flx^ MAFs infected with empty vector-GFP (EV) or Cre recombinase-GFP (CRE) adenoviruses and transfected with scramble (scr) or Mic60 siRNA (2). Scale bars, 2 μm (upper panel) and 500 nm (lower panel). See also Figure S3A. (B) Quantification of CJ number per crista in experiments as in (A). Data represent mean ± SEM of four independent experiments (n = 100–150 mitochondria per condition). ^∗^p < 0.05 in a paired sample Student’s t test between EV-scr and all other conditions. (C) CLW analysis in experiments as in (A). Data represent mean ± SEM of four independent experiments (n = 150–400 cristae per condition). ^∗∗∗^p < 0.001 in a paired sample Student’s t test between EV-scr and CRE-scr; ^∗∗^p < 0.01 in a paired sample Student’s t test between EV-scr and EV-Mic60 siRNA; ###p < 0.001 in a paired sample Student’s t test EV-Mic60 siRNA and CRE-Mic60 siRNA. (D) Representative EM of mitochondria from MAFs of the indicated genotype transfected where indicated using scr or Mic60 siRNA (2). Scale bars, 2 μm (upper panel) and 500 nm (lower panel). See also Figures S3B and S3C. (E) Quantification of CJ number per crista in experiments as in (D). Data represent mean ± SEM of four independent experiments (n = 100–150 per condition). ###p < 0.001 in one-way ANOVA between wt-scr and *Opa1*^tg^-scr; ^∗∗∗^p < 0.001 in one-way ANOVA between wt-Mic60 siRNA and *Opa1*^tg^-Mic60 siRNA; ^∗∗^p < 0.01 in a paired sample Student’s t test between wt-scr and wt-Mic60 siRNA; ##p < 0.01 in a paired sample Student’s t test between *Opa1*^tg^-scr and *Opa1*^tg^-Mic60 siRNA. (F) CLW analysis in experiments as in (D). Data represent mean ± SEM of three independent experiments (n = 130–300 cristae per condition). ^∗∗∗^p < 0.001 in one-way ANOVA between WT and *Opa1*^tg^-scr; ###p < 0.001 in one-way ANOVA between wt-Mic60 siRNA and *Opa1*^tg^-Mic60 siRNA; ^∗∗^p < 0.01 in a paired sample Student’s t test between wt-scr and wt-Mic60 siRNA.

**Figure 4:**
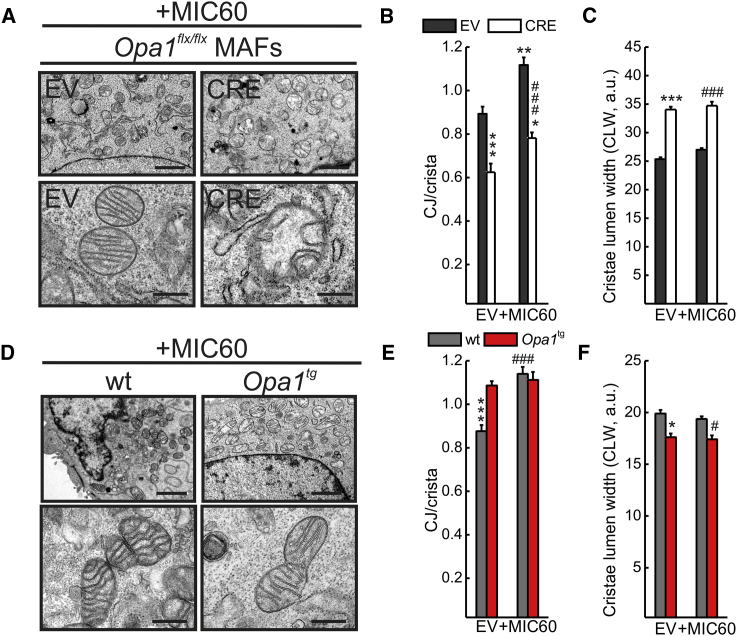
OPA1 Is Epistatic to MIC60 in the Control of the CJ Number (A) Representative EM of *Opa1*^flx/flx^ MAFs infected and transfected as indicated. Scale bars, 2 μm (upper panel) and 500 nm (lower panel). See also Figure S4A. (B) Quantification of CJ number per crista in experiments as in (A). Data represent mean ± SEM of four independent experiments (n = 100–150 per condition). ^∗∗∗^p < 0.001 in a paired sample Student’s t test between EV-(EV) and CRE-(EV); ^∗∗^p < 0.01 in a paired sample Student’s t test between EV-(EV) and EV-(+MIC60); ^∗^p < 0.05 in a paired sample Student’s t test between CRE-(EV) and CRE-(+MIC60); ###p < 0.001 in a paired sample Student’s t test between EV-(EV) and CRE-(+MIC60). (C) CLW analysis in experiments as in in (A). Data represent mean ± SEM of four independent experiments (n = 150–250 cristae per condition). ^∗∗∗^p < 0.001 in a paired sample Student’s t test between EV-(EV) and CRE-(EV); ###p < 0.001 in a paired sample Student’s t test between EV-(+MIC60) and CRE-(+MIC60). (D) Representative EM of mitochondria from MAFs of the indicated genotype transfected as indicated. Scale bars, 2 μm (upper panel) and 500 nm (lower panel). See also Figure S4C. (E) Quantification of CJ number per crista in experiments as in (D). Data represent mean ± SEM of four independent experiments (n = 100–150 per condition). ^∗∗∗^p < 0.001 in one-way ANOVA between wt-(EV) and *Opa1*^tg^-(EV) or *Opa1*^tg^-(+MIC60); ###p < 0.001 in a paired sample Student’s t test between wt-(EV) and wt-(+MIC60). (F) CLW analysis in experiments as in (D). Data represent mean ± SEM of four independent experiments (n = 100–150 cristae per condition). ^∗^p < 0.05 in one-way ANOVA between wt-(EV) and *Opa1*^tg^-(EV); #p < 0.05 in a one-way ANOVA between wt-(+MIC60) and *Opa1*^tg^-(+MIC60).

The physical interaction between MIC60 and OPA1 in the ∼720 kDa complex that is targeted during cristae remodeling prompted us to investigate their relative role in cristae and CJ biogenesis. We therefore turned to electron microscopy (EM) and morphometric analysis to perform a genetic epistatic analysis, capitalizing on *Mic60* silencing or overexpression in established models of acute *Opa1* ablation by means of adenoviral CRE delivery to *Opa1*^flx/flx^ MAFs, or permanent, mild, transgene-driven *Opa1* overexpression (*Opa1*^tg^ MAFs). When we efficiently silenced *Mic60* in empty vector (EV)-infected *Opa1*^flx/flx^ MAFs (Figure S3A), the number of CJs per crista (Figures 3A and 3B) and number of cristae with CJ (Figure S3E) were reduced by ∼30%, similar to previous reports in yeast and mammals (John et al., 2005, Rabl et al., 2009). However, *Opa1* deletion (Figure S3A) per se induced a significant reduction in the number of CJs per crista (Figures 3A and 3B) and number of cristae with CJ (Figures S3D and S3E). Combined *Mic60* downregulation and *Opa1* ablation induced a quasi-complete reduction in OPA1 and MIC60 levels (Figure S3A) but did not further reduce the number of CJs per crista (Figures 3A and 3B) and number of cristae with CJ (Figure S3E). Thus, OPA1 and MIC60 are components of the same pathway regulating CJ biogenesis. Conversely, *Mic60* silencing induced a minor increase in CLW, which was dramatically increased upon *Opa1* deletion (Figures 3A and 3C). Cristae were not further widened when *Mic60* was ablated following *Opa1* deletion (Figures 3A and 3C), indicating that OPA1 acts as a master regulator of CLW. In *Opa1*^tg^ MAFs, where OPA1 is mildly overexpressed (Figure S3B), the number of CJs per crista (Figures 3D and 3E) and number of cristae with CJ (Figure S3F) were increased; efficient *Mic60* silencing (Figure S3C) reduced both the number of CJs per crista (Figures 3D and 3E) and the number of cristae with CJ (Figure S3F), irrespective of whether OPA1 was overexpressed. Conversely, CLW was narrower in *Opa1*^tg^ MAFs and was not affected by *Mic60* silencing (Figures 3D and 3F), confirming the main role of OPA1 in the regulation of CLW. To relatively position MIC60 and OPA1 in the pathway controlling CJ biogenesis, we completed our epistatic analysis by measuring the same parameters of CJ number and CLW in sorted GFP^+^ and RFP^+^ cells upon infection with the GFP-expressing adenoviruses and MIC60-V5/mtRFP cotransfection. As expected, MIC60-V5 expression in EV-infected *Opa1*^flx/flx^ MAFs (Figure S4A) increased the number of CJs per crista (Figures 4A and 4B) and number of cristae displaying a CJ (Figure S4B), whereas it did not have any effect on CLW (Figures 4A and 4C). The number of CJs per crista was reduced when *Opa1* was deleted; when MIC60-V5 was expressed in *Opa1*-deleted cells (at levels comparable to those achieved in EV-infected *Opa1*^flx/flx^ MAFs) (Figure S4A), the number of CJs per crista increased only marginally, and it did not reach the levels obtained upon MIC60-V5 expression in EV-infected *Opa1*^flx/flx^ MAFs (Figures 4A and 4B). The same picture was observed when we measured the percentage of cristae displaying a CJ (Figure S4B). As expected, MIC60 overexpression was not able to reduce the cristae lumen widening caused by *Opa1* ablation (Figures 4A and 4C). Finally, MIC60 overexpression (Figure S4C) did not further increase the number of CJs per crista (Figures 4D and 4E) and number of cristae displaying a CJ (Figure S4D), nor it did further narrow CLW in *Opa1*^tg^ MAFs (Figures 4D and 4F). In conclusion, OPA1 and MIC60 cooperate to control mammalian CJ biogenesis, OPA1 being placed upstream of MIC60. Conversely, CLW is controlled solely by OPA1.

### OPA1 Specifies Cristae Junction Width Independently of MIC60

**Figure 5:**
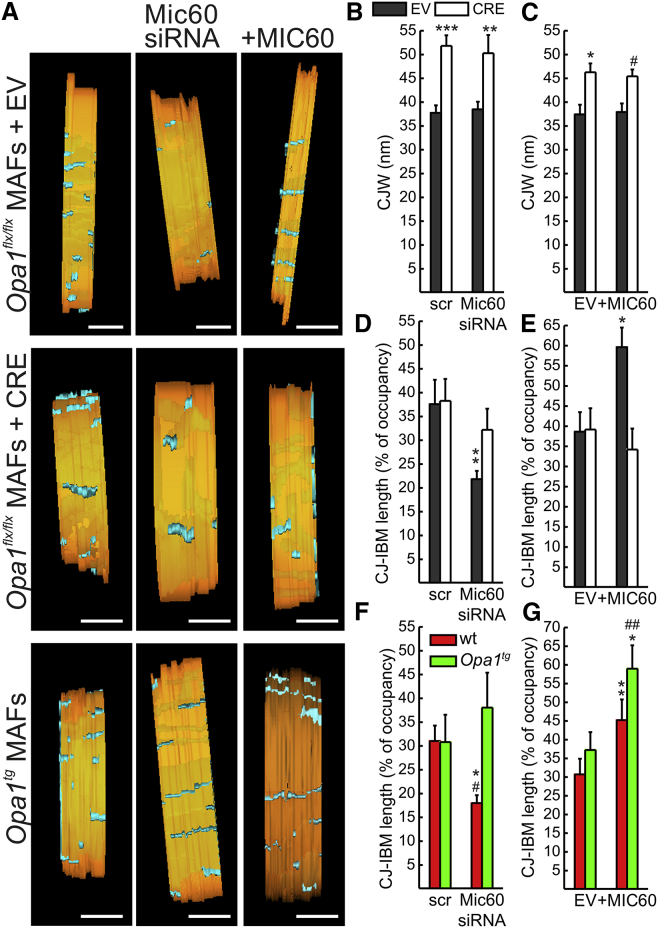
OPA1 Is Epistatic to MIC60 in the Control of CJ Stability and CJW (A) Surface-rendered views of representative tomographic reconstructions of mitochondria from MAFs of the indicated genotype infected and transfected as indicated. Scr, scramble; +MIC60, MIC60 overexpression; EV, empty vector; Mic60 siRNA, siRNA (3); CRE, Cre recombinase. The inner membrane is pseudocolored in orange, and cristae are pseudocolored in cyan. The outer membrane has been electronically peeled out to highlight the CJ. Scale bars, 120 nm. (B and C) Quantification of CJW in experiments as in (A). Data represent mean ± SEM of three independent experiments (three to four tomograms per condition). (B) ^∗∗∗^p < 0.001 in a paired sample Student’s t test between EV-scr and CRE-scr; ^∗∗^p < 0.01 between EV-Mic60 siRNA and CRE-Mic60 siRNA. (C) ^∗^p < 0.05 in a paired sample Student’s t test between EV-(EV) and CRE-(EV); #p < 0.05 between EV-(+MIC60) and CRE-(+MIC60). (D–G) Quantification of the percentage of IBM occupied by a CJ in experiments as in (A). Data represent average ± SEM of three independent experiments (three to four tomograms per condition). (D) ^∗∗^p < 0.01 in a paired sample Student’s t test between EV-Mic60 siRNA and all other conditions. (E) ^∗^p < 0.05 in a paired sample Student’s t test between EV-(+MIC60) and all other conditions. (F) ^∗^p < 0.05 in one-way ANOVA between wt-Mic60 siRNA and *Opa1*^tg^-scr or *Opa1*^tg^-Mic60 siRNA; #p < 0.05 in a paired sample Student’s t test between wt-Mic60 siRNA and wt-scr. (G) ^∗^p < 0.05 in one-way ANOVA between *Opa1*^tg^-(+MIC60) and wt-(+MIC60); ^∗∗^p < 0.01 in a paired sample Student’s t test between wt-(EV) and wt-(+MIC60); ##p < 0.01 in a paired sample Student’s t test between *Opa1*^tg^-(EV) and *Opa1*^tg^-(+MIC60). See also Figure S5.

OPA1 oligomers also control CJW (Figure S5) (Frezza et al., 2006), crucial to limiting cytochrome *c* mobilization from the cristae compartment to the IMS and hence to modulating apoptosis. We therefore wished to understand whether MIC60 cooperated with OPA1 to regulate CJW. To this end, we acquired electron tomograms of mitochondria in the same cellular models employed before for the epistatic analysis of the relative position of OPA1 and MIC60 in CJ biogenesis. Rotations of representative surface that were rendered views of tomographic reconstructions of mitochondria allowed to highlight the individual openings of the CJs (cyan) into the IBM (orange in Figures 5A and S5) and to measure the CJW (Figure S5). Acute *Opa1* ablation increased CJW by ∼35%–40% (37.74 ± 1.57 nm in EV-infected *Opa1*^flx/flx^ mitochondria versus 51.82 ± 2.20 nm in CRE-infected *Opa1*^flx/flx^ mitochondria; n = 20 CJ in three to four independent tomograms) (Figures 5A and 5B), whereas CJW was not affected by *Mic60* silencing (37.74 ± 1.57 nm in control-silenced mitochondria versus 38.48 ± 1.61 nm in *Mic60*-silenced mitochondria; n = 20 CJ in three to four independent tomograms) (Figures 5A and 5B) or overexpression (37.42 ± 2.09 nm in control-transfected mitochondria versus 37.91 ± 1.81 nm in MIC60-transfected mitochondria; n = 20–26 CJ in three independent tomograms) (Figures 5A and 5C). In conclusion, OPA1 specifies independently of MIC60 not only cristae lumen but also cristae junction width.

### MIC60 Requires OPA1 to Stabilize the Inner Membrane-Crista Junction

A closer inspection of tomograms from MIC60-transfected mitochondria revealed a striking increase in the major axis of otherwise normal, narrow CJs (Figure 5A, top right panel). We therefore decided to measure whether levels of MIC60 influenced the CJ-IBM length, i.e., the relative IBM occupancy by an individual CJ (Figure S5). *Mic60* silencing decreased this parameter, whereas its overexpression increased it. While *Opa1* ablation per se did not affect CJ-IBM length, it abolished the increase induced by MIC60 overexpression (Figures 5A, 5D, and 5E); accordingly, OPA1 overexpression blunted the decrease in CJ-IBM length caused by *Mic60* silencing, without influencing per se the degree of IBM occupancy by CJs (Figures 5A, 5F, and 5G). In conclusion, stability of the junction between cristae and IBM requires MIC60; however, OPA1 is epistatic to MIC60 in this function.

### MIC60 Is Dispensable for Apoptotic Cristae Remodeling and Cytochrome *c* Mobilization

**Figure 6:**
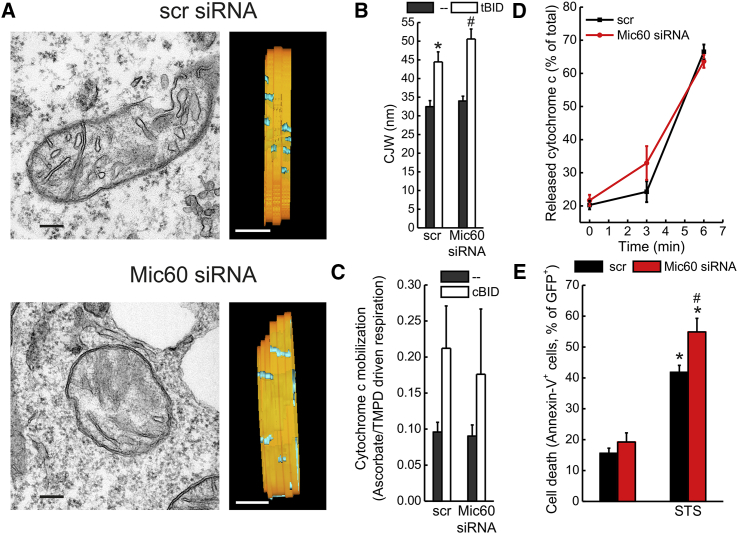
MIC60 Does Not Control Apoptotic Cristae Remodeling and Cytochrome Release (A) Representative electron micrographs (left panels) and rotations of surface-rendered views of tomographic reconstructions (right panels) of mitochondria from MAFs transfected with scramble (scr) or Mic60 siRNA (2) and transduced with tBID-GFP-expressing retroviruses. In tomographic reconstructions, the inner membrane is depicted in orange and the CJ is depicted in cyan. The outer membrane has been omitted to highlight the CJ. Scale bars, 200 nm (EM) and 120 nm (tomograms). (B) Quantification of the CJW in experiments as in (A). Data represent average ± SEM of three independent experiments (n = 3 tomograms per condition). ^∗^p < 0.05 in a paired sample Student’s t test between scr-tBID and scr-untreated (−); #p < 0.05 in a paired sample Student’s t test between Mic60 siRNA-tBID and Mic60 siRNA-untreated (−). (C) Ascorbate/TMPD-driven respiration of mitochondria isolated from MAFs transfected with scramble (scr) or Mic60 siRNA (1–3) and treated where indicated with cBID. Data represent average ± SEM of three independent experiments. (D) Isolated mitochondria from MAFs transfected as indicated with scr or siRNA Mic60 (1–3) were treated with cBID, and cytochrome *c* release was measured at the indicated time points. Data represent mean ± SEM of four independent experiments. (E) MAFs transfected with scramble (scr) or Mic60 siRNA (1–3) were treated where indicated with 2 μM staurosporine (STS) for 6 hr, and cell death was determined cytofluorimetrically. Data represent mean ± SEM of three independent experiments. ^∗^p < 0.05 in a paired sample Student’s t test between scr-STS and scr-untreated and between Mic60 siRNA-STS and Mic60 siRNA-untreated; #p < 0.05 in a paired sample Student’s t test between Mic60 siRNA-STS and scr-STS.

The extent of CJ-IBM junction might influence cytochrome *c* redistribution, release, and cell death. In such a model, *Mic60* silencing, by reducing the cristae-IBM junction, should inhibit intramitochondrial cytochrome *c* redistribution, release, and apoptosis. However, quantitation of electron tomograms indicated that *Mic60* silencing did not reduce the CJ widening triggered by the proapoptotic stimulus cBID (Figures 6A and 6B); accordingly, cytochrome *c* mobilization (Figure 6C) and release (Figure 6D) were not affected. Conversely, apoptosis was slightly increased by *Mic60* ablation (Figure 6E), opposite to what the model predicted. This marginal increase in cell death possibly reflects the mitochondrial fragmentation (Figures S6A and S6B) and dysfunction (Figures S6C and S6D) caused by *Mic60* ablation. In conclusion, the MIC60-controlled CJ-IBM length does not participate in the apoptotic redistribution of cytochrome *c* and apoptosis.

## Discussion

How membranes are shaped is a crucial question in biology. The case of the inner mitochondrial membrane is particularly interesting because of its organization into two separate yet connected compartments, the IBM and cristae (Frey and Mannella, 2000). The discovery of OPA1 as a master regulator of cristae remodeling was instrumental to probe the role of cristae shape in mitochondrial bioenergetics (Cogliati et al., 2013), apoptosis (Frezza et al., 2006, Yamaguchi et al., 2008), and tissue damage (Varanita et al., 2015). Studies in yeast identified an essential role for MICOS, a large multiprotein complex whose components are partially conserved up to *H. sapiens*, in CJ formation, raising the question of the role of OPA1 in cristae biogenesis and MICOS in cristae remodeling in mammals. Proteomics and biochemistry identify complexes composed of, and physical interactions between, the core MICOS component MIC60 and OPA1. Genetics, electron tomography, and functional studies identify that OPA1 is epistatic to MIC60 in the regulation of CJ number and stability. OPA1 is the sole regulator of CJW and CLW, explaining the marginal effect of MIC60 on cytochrome *c* redistribution, release, and apoptosis.

While in yeast the central MICOS component MIC60 controls not only mitochondrial protein import (Körner et al., 2012, Pfanner et al., 2014, Stojanovski et al., 2012) and inner-outer membrane contact sites (Ott et al., 2012, Xie et al., 2007) but especially cristae and CJ biogenesis (Alkhaja et al., 2012, Darshi et al., 2011, John et al., 2005, Körner et al., 2012), in mammals MIC60 and MICOS complex function is less defined. Mammals lack some MICOS components (Muñoz-Gómez et al., 2015a, Muñoz-Gómez et al., 2015b), and our bioinformatics analysis identified some striking primary sequence differences even in the strongly conserved MIC60. At a major difference from yeast, *Chordata* MIC60 harbors a highly conserved TMH with a glycine cluster that probably facilitates interaction with other proteins. This highly conserved TMH appeared in evolution, together with the use of mitochondrial cytochrome *c*, to amplify intrinsic apoptosis, suggesting that mammalian MIC60 could participate in the mitochondrial apoptotic remodeling controlled by OPA1 (Cogliati et al., 2013, Frezza et al., 2006, Jiang et al., 2014, Yamaguchi et al., 2008).

In mammalian mitochondria, MIC60 is retrieved also in ∼720 kDa HMWCs (Guarani et al., 2015, Li et al., 2016), the same MW of the OPA1 complexes targeted during cristae remodeling. Proteomic analysis of the ∼720 kDa OPA1-containing complexes targeted during cristae remodeling in heart and fibroblast mitochondria identified MIC60 and the other MICOS component, MIC19. In a proteomic repertoire of mitochondrial proteins co-immunoprecipitated with tagged MICOS components, OPA1 was not cataloged (Guarani et al., 2015). However, MIC60 tagging might have perturbed the MIC60-OPA1 interaction, and several other lines of evidence substantiate the MICOS-OPA1 interaction: (1) in the same complexes targeted during cristae remodeling, we retrieved not only MIC60 and MIC19 but also the MICOS components and interactors QIL1, SAMM50, MTX2, and SLC25A12; (2) OPA1 and MIC60 directly interact; and (3) *Opa1* or *Mic60* deletion destabilizes this HMWC. When *Opa1* is depleted, MIC60 is retrieved not in ∼720 kDa complexes but in >1 MDa complexes; when *Mic60* is downregulated, a new ∼650 kDa OPA1-containing complex appears, compatible with the loss of MIC60 from the ∼720 kDa complex. The partial destabilization of the ∼720 kDa OPA1 complex can also lend a molecular explanation to the marginal effect of *Mic60* ablation on CLW, mitochondrial bioenergetics, and cell death, all controlled by this OPA1 complex (Cogliati et al., 2013).

MIC60 and OPA1 not only interact physically but also are components of the same pathway regulating cristae biogenesis. We addressed the roles of MIC60 and OPA1 on a handful of EM or ET cristae morphometric parameters: CJ number, CJW, CJ-IBM occupancy (an indicator of CJ stability), and cristae lumen width (a parameter correlating with RCSs assembly and mitochondrial respiratory efficiency) (Cogliati et al., 2013). This comprehensive morphometry analysis revealed that (1) OPA1 is the sole regulator of CLW and CJW, (2) OPA1 and MIC60 lie in the same pathway controlling CJ number and stability, and (2) surprisingly, OPA1 is epistatic to MIC60. While this work was under revision, Barrera et al., (2016) confirmed that OPA1 and MIC60 interact physically and that levels of the former affect CJ number. However, whether they lie in the same genetic pathway and how they are relatively positioned in the control of the multiple parameters of cristae shape was not addressed (Barrera et al., 2016).

In principle, our genetic analysis does not address whether the epistatic effect of OPA1 is direct or mediated, for example, by the stimulation of ATP synthase dimerization (Patten et al., 2014). Dimers of ATP synthase modulate cristae shape at least in yeast (Paumard et al., 2002), suggesting that a genetic analysis similar to the one presented here should be performed to dissect the relative role of ATPase dimerization subunits in OPA1-MIC60-controlled CJ biogenesis. However, the discovery of a physical interaction between OPA1 and MIC60 strongly suggests that OPA1 directly effects MIC60 to control cristae shape. Our epistatic analysis can also explain molecularly why mild OPA1 overexpression can inhibit apoptotic cristae remodeling and correct models of primary mitochondrial dysfunction with altered cristae shape (Civiletto et al., 2015, Varanita et al., 2015): even if other major cristae biogenesis components like MICOS are altered, OPA1 per se ameliorates all cristae biogenesis parameters. Finally, they can rationalize why *Mic60* silencing reduces CJ number but paradoxically increases cytochrome *c* release and apoptosis (John et al., 2005, Van Laar et al., 2016, Yang et al., 2012): *Mic60* ablation destabilizes the OPA1-containing complex and results in mitochondrial fragmentation and dysfunction, both of which can contribute to cell death.

Our work unravels that OPA1 epistatically influences the core MICOS component MIC60 in CJ biogenesis and assigns OPA1 the key role in the control of apoptotic cristae remodeling. These discoveries pave the way toward investigating how cristae shape coordinates with OPA1-dependent mitochondrial fusion.

## Experimental Procedures

### Molecular Biology

Details on pcDNA6.2-hMic60-V5 generation, mtRFP plasmids, Mic60 targeting siRNAs, and cBID production can be found in the Supplemental Information.

### Cell Culture, Transfection, and Infection

Details on the used cell types can be found in the Supplemental Information. Acute *Opa1* ablation in *Opa1*^flx/flx^ MAFs was obtained by infection with adenoviruses expressing cytomegalovirus (CMV)-Cre-GFP (CRE; 200 pfu/cell; Vector BioLabs) or (CMV)-GFP (EV; Vector BioLabs). Infection efficiency was typically around 80% after 48 hr, as determined by counting GFP-positive cells. Details on simultaneous MIC60 overexpression or silencing and *Opa1* ablation can be found in the Supplemental Information.

### Mitochondrial Isolation and In Vitro Assays

Mitochondrial isolation and functional assays were performed as in Frezza et al. (2007). Details can be found in the Supplemental Information.

### Biochemistry

Total cell lysates prepared in RIPA buffer were separated under denaturing conditions in Tris-acetate 3%–8% or Bis-Tris 4%–12% (NuPAGE, Life Technologies) polyacrylamide gels, transferred onto polyvinylidene fluoride (PVDF) membranes (Millipore), and probed using the indicated antibodies. Further details on the used antibodies can be found in the Supplemental Information. Details on chemical crosslinking can also be found in the Supplemental Information.

### BN-BN-SDS-GE

Digitonin (1.25%, Life Technologies) extracted protein complexes from mitochondria purified from heart (250 μg) or MAFs of the specified genotype after the indicated genetic manipulation (150 μg) were separated by blue native gel electrophoresis (BNGE) on a precast native Bis-Tris 3%–12% gel (Life Technologies).

Two-dimensional (2D) blue native-blue native gel electrophoresis (BN-BNGE) was performed by excising and casting the lane obtained from the first BNGE onto a single-well native gel (NuPAGE Novex Bis-Tris 4%–12% ZOOM gel; Life Technologies), adding 0.02% n-dodecyl-D-maltoside (DDM; Sigma) in the cathode buffers.

Third dimensional (3D) SDS gel electrophoresis (SDS-GE) was performed after excising the diagonal and incubating it with reducing solutions (RSs) RSa for 10 min, RSb for 7 min, and RSc for 12 min to facilitate the complexes’ dissociation during the 3D SDS-GE. After the treatment, the diagonal was loaded onto a Bis-Tris 4%–12% ZOOM NuPAGE Novex gel (Life Technologies) and run under denaturating conditions to separate the individual proteins from the complexes, which were then transferred onto PVDF membrane and probed using the indicated antibodies. Further details can be found in the Supplemental Information.

### BNGE-Based Semiquantitative Proteomic Analysis, SILAC Labeling, and Quantitative Proteomic Analysis

Details on BNGE-based semiquantitative proteomic analysis can be found in the Supplemental Information. For SILAC labeling, MAFs were grown separately in DMEM containing 4.5 g/L glucose, 2 mM glutamine, 10% fetal bovine serum (FBS), 50 U/mL penicillin, 50 mg/mL streptomycin, 50 mg/mL uridine, and phenol red supplemented with either light L-lysine and L-arginine or heavy [U-13C6]-L-lysine HCl and [U-13C6]-L-arginine (100 mg/L of each amino acid) (SILAC Protein Identification and Quantification Media Kit; Invitrogen). After six doublings, light and heavy cell subpopulations were harvested and mitochondria were isolated separately as described previously. Details on treatments, BNGE, tryptic digestion, liquid chromatography-mass spectrometry (LC-MS), protein identification, and peptide quantification can be found in the Supplemental Information.

### Electron Microscopy and Tomography

Electron microscopy and electron tomography were performed as previously described. Details can be found in the Supplemental Information.

### Mitochondrial Morphometry

Mitochondrial parameters were measured using ImageJ (NIH) by two operators blinded to the identity of the sample. Cristae lumen width was quantified with the ImageJ Freehand line selection tool. Sample size is indicated in the figure legends.

## Author Contributions

Conceptualization, M.E.S. and L.S.; Methodology, E.C., J.V., J.A.E., L.S., and M.E.S.; Software, E.C. and J.V.; Validation, C.G., S.C., and M.E.S.; Formal Analysis, C.G., E.C., J.V., M.L., L.P., and M.E.S.; Investigation, C.G., S.C., A.M., I.A., G.R., E.C., A.R., N.S., and M.E.S.; Resources, J.A.E., J.V., M.E.S., and L.S.; Data Curation, C.G., E.C., L.P., and M.E.S.; Writing – Original Draft, C.G., L.P., L.S., and M.E.S.; Writing – Review & Editing, C.G., M.E.S., and L.S.; Visualization, C.G., E.C., L.P., L.S., and M.E.S., Supervision, M.E.S.; Project Administration, M.E.S.; Funding Acquisition, L.S. and M.E.S.

## Supplemental Information

Document S1. Supplemental Experimental Procedures and Figures S1–S6

Document S2. Article plus Supplemental Information
