# Supplementary material for: Optic Atrophy 1 Is Epistatic to the Core MICOS Component MIC60 in Mitochondrial Cristae Shape Control
Source: Cell Rep. 2016 Dec 13;17(11):3024–34. doi: 10.1016/j.celrep.2016.11.049 (PMC5186903; doi:10.1016/j.celrep.2016.11.049)
Supplement: Document S1. Supplemental Experimental Procedures and Figures S1–S6 [file mmc1.pdf]

Cell Reports, Volume *17*

Supplemental Information

Optic Atrophy 1 Is Epistatic to the Core MICOS Component MIC60 in Mitochondrial Cristae Shape Control

Christina Glytsou, Enrique Calvo, Sara Cogliati, Arpit Mehrotra, Irene Anastasia, Giovanni Rigoni, Andrea Raimondi, Norihito Shintani, Marta Loureiro, Jesùs Vazquez, Luca Pellegrini, Jose Antonio Enriquez, Luca Scorrano, and Maria Eugenia Soriano

Supplemental Figures

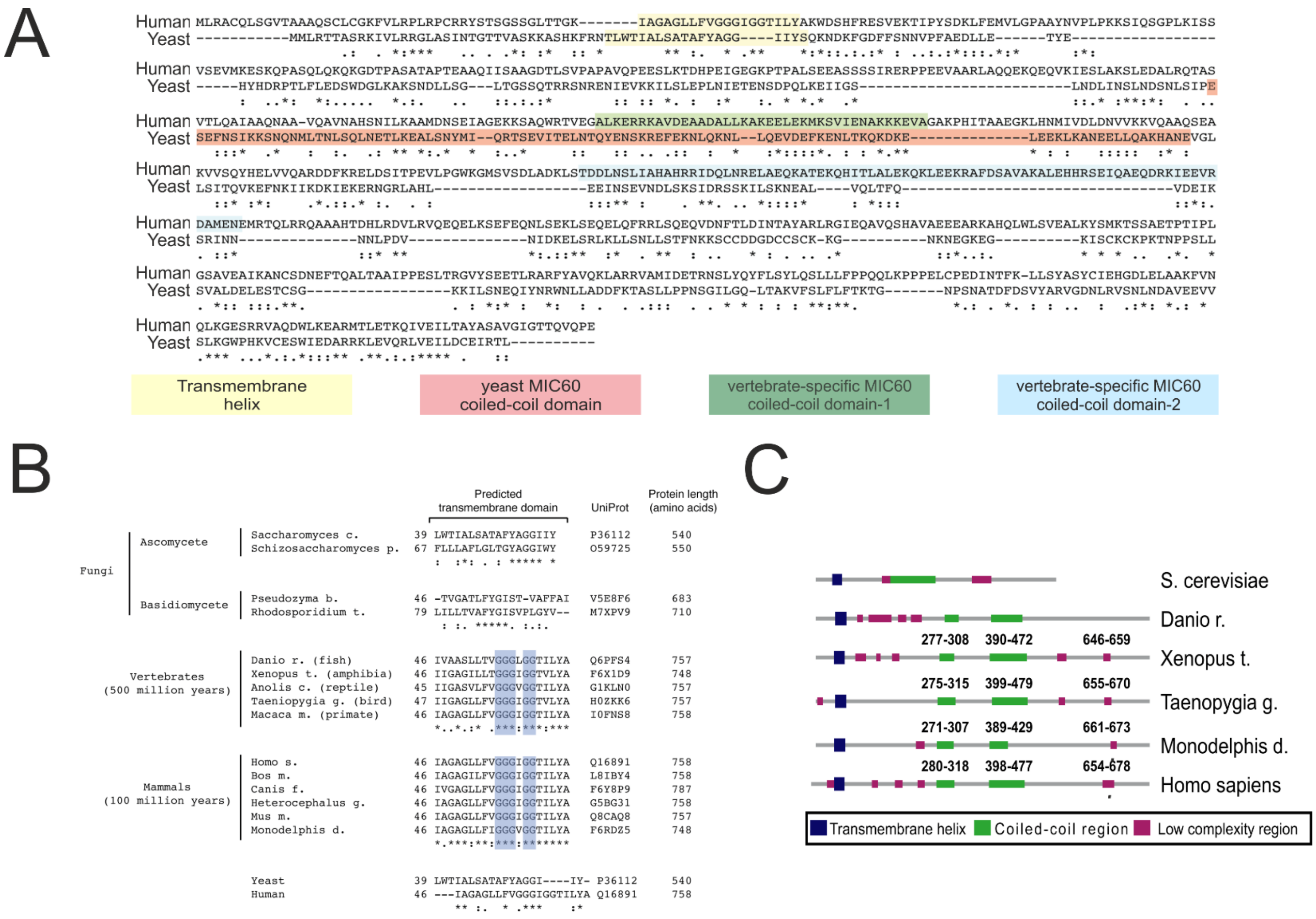

Figure S1. Comparative evolutionary analysis of MIC60 homologues in fungi and vertebrates. Related to Figure 1.

(A) Bioinformatic analysis showing limited evolutionary and structural conservation between yeast and mammalian MIC60 homologs. The predicted transmembrane and coil-coiled domains are highlighted.

(B) Multiple amino acid sequence alignment of the predicted transmembrane domain of MIC60 homologues identified in the genomes of key eukaryotic model organisms (fungi, vertebrates, and mammals). The boxed areas indicate the clusters of glycine residues.

(C) Spatial and structural organization of the predicted protein domains that compose MIC60 homologues showing conservation among vertebrates but not between mammals and yeast.

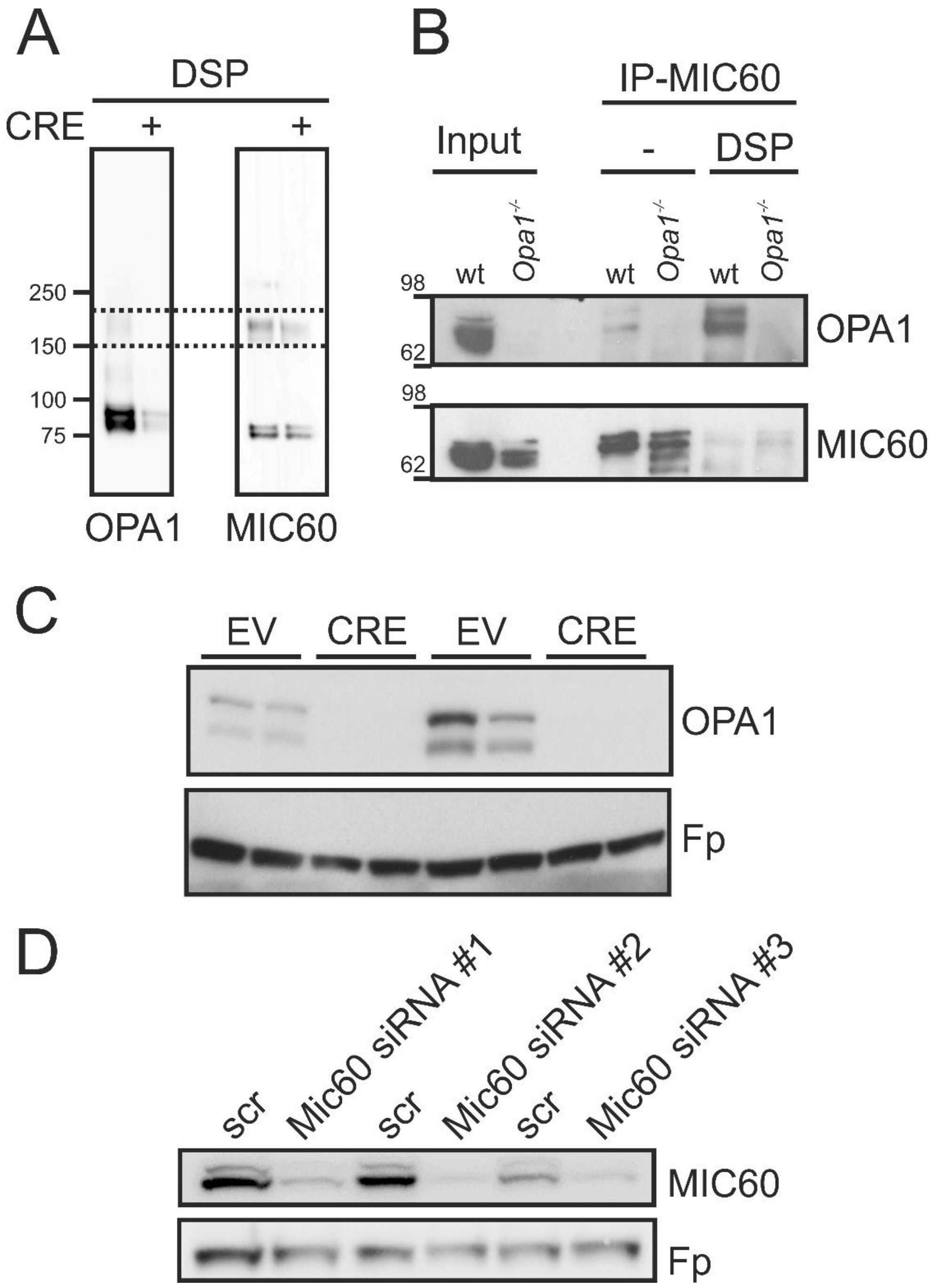

Figure S2. OPA1-MIC60 crosslinked complexes co-immunoprecipitate. Related to Figure 2.

(A) Isolated mitochondria from *Opa1*$^{flx/flx}$ MAFs infected with the indicated adenoviruses (EV, empty vector-GFP; CRE, Cre recombinase-GFP) were crosslinked with 2 mM DSP and equal amounts (20 µg) of proteins were separated in Tris-Acetate 3%-8% gel and immunoblotted with the indicated antibodies. The boxed are shows OPA1 and MIC60 complexes with the same electrophoretic migration.

(B) Lysates from DSP-crosslinked or native mitochondria (125 µg) were immunoprecipitated with anti-MIC60 antibody coupled to Protein-A agarose beads and bound proteins were separated by SDS-GE and immunoblotted using the indicated antibodies. Input is diluted 1:10.

(C) Western blot analysis of OPA1 protein levels after acute ablation. Equal amounts of proteins (40 µg) from *Opa1*$^{flx/flx}$ MAFs infected with the indicated adenoviruses (EV, empty vector-GFP; CRE, Cre recombinase-GFP) were separated by SDS-GE and immunoblotted with the indicated antibodies.

(D) Western blot analysis of MIC60 protein levels after silencing. Equal amounts of proteins (40 µg) from MAFs transfected with scramble (scr) or three independent Mic60 siRNAs (#1, #2 or #3) were separated by SDS-GE and immunoblotted with the indicated antibodies. All three Mic60 siRNA oligos were used independently to perform all the experiments where *Mic60* silencing is indicated. For each figure the specific Mic60 siRNA used is indicated in the figure legend.

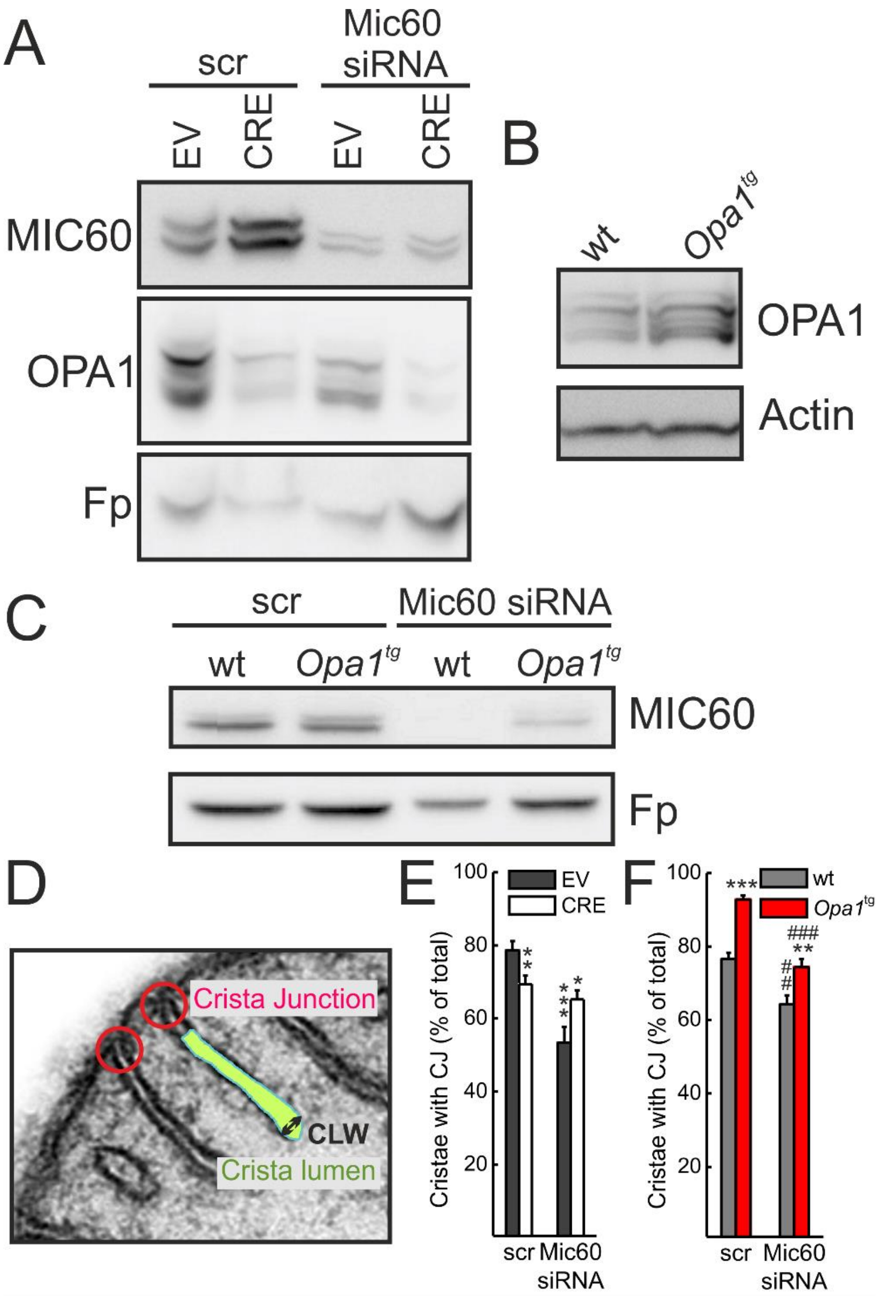

Figure S3. MIC60 and OPA1 are in the same pathway controlling CJ biogenesis. Related to Figure 3.
(A) Protein levels of MIC60 and OPA1 after *Mic60* knock-down (Mic60 siRNA #2) and/or *Opa1* acute ablation *Opa1*$^{flx/flx}$ MAFs. Equal amounts of protein (25 µg) were loaded onto a Tris-Acetate 3%-8% gel and immunoblotted with the indicated antibodies. EV: empty vector-GFP; CRE: Cre recombinase-GFP; scr: scramble; Mic60 siRNA: MIC60 silencing.
(B) OPA1 protein levels in wt and *Opa1*^{tg} MAFs. Equal amounts of protein samples (25 µg) were separated in a Tris-Acetate 3%-8% gel and immunoblotted with the indicated antibodies.
(C) MIC60 protein levels after *Mic60* knock-down (Mic60 siRNA #2) in wt and *Opa1*^{tg} MAFs. Equal amounts of protein samples (25 µg) were separated in a Tris-Acetate 3%-8% gel and immunoblotted with the indicated antibodies.
(D) Scheme illustrating the CJ (red circles) and CLW (black arrow) parameters. Cristae lumen is colored in green.
(E) Percentage of cristae with CJ in 100-150 randomly selected mitochondria from each experimental condition, as indicated. Data represent average ± SEM of 4 independent experiments. ***p<0.001 in a paired sample Student's t-test between EV-scr and -Mic60 siRNA; **p < 0.01 in a paired sample Student's t-test between EV- and CRE-scr; *p<0.05 in a paired sample Student's t-test between EV- and CRE-Mic60 siRNA.
(F) Percentage of cristae with CJ in 100-150 randomly selected mitochondria from each experimental condition, as indicated. Data represent average ± SEM of 4 independent experiments. ***p<0.001 in one-way ANOVA between wt- and *Opa1*^{tg}-scr; ## p<0.01 in a paired sample Student's t-test between wt-scr and -Mic60 siRNA; ### p<0.001 in a paired sample Student's t-test between *Opa1*^{tg}-scr and -Mic60 siRNA*;* **p<0.01 in one-way ANOVA between wt- and *Opa1*^{tg}-Mic60 siRNA.

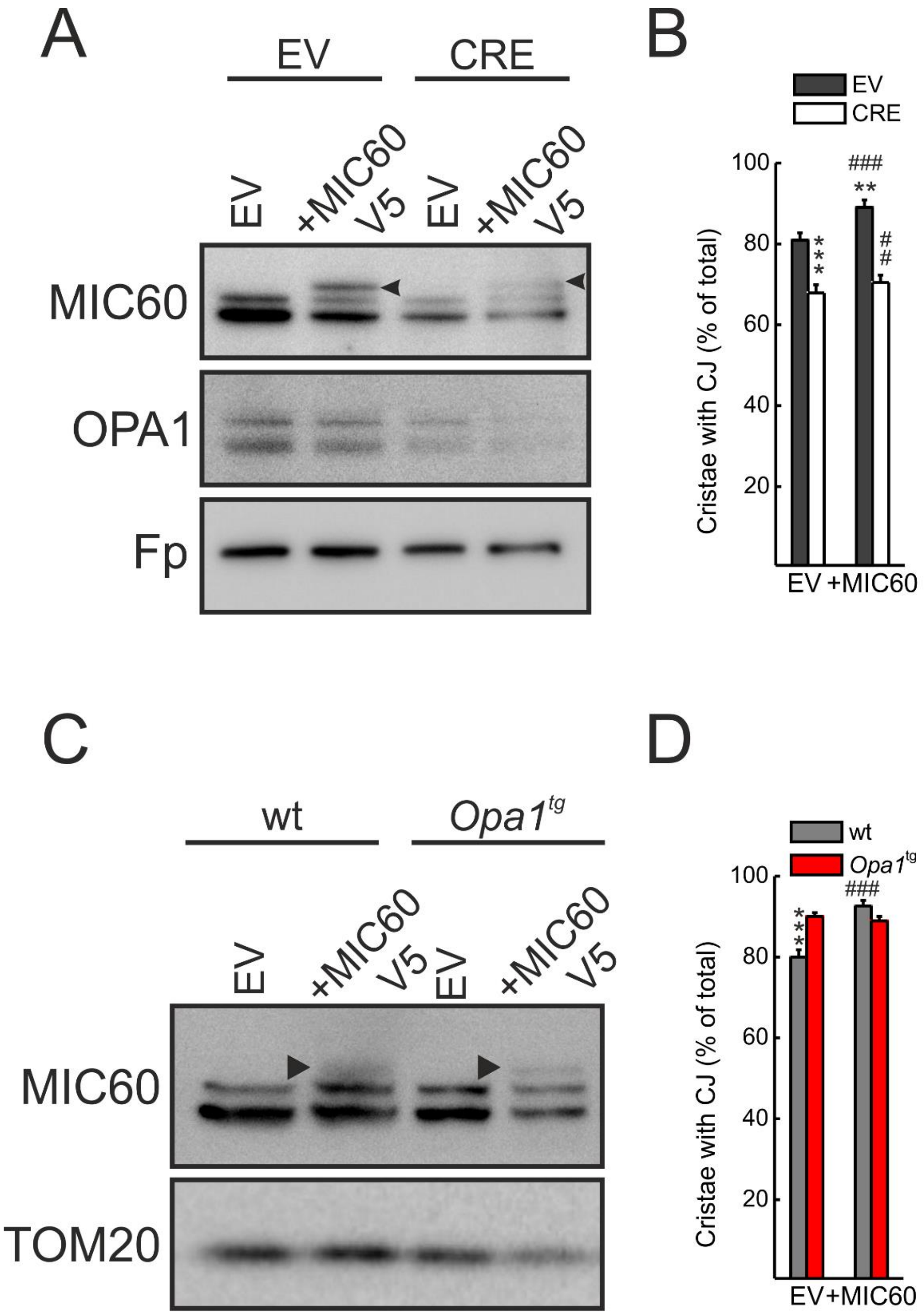

Figure S4. OPA1 is epistatic to MIC60 in regulation of CJ biogenesis. Related to Figure 4.
(A) Protein levels of MIC60 and OPA1 after MIC60 overexpression and/or *Opa1* acute ablation in *Opa1*$^{flx/flx}$ MAFs. Equal amounts of protein lysates (25 μg) were loaded onto a 3%-8% Tris-Acetate gel and immunoblotted with the indicated antibodies. EV: empty vector-GFP; CRE: Cre recombinase-GFP; +MIC60-V5: overexpression of MIC60.
(B) Percentage of cristae with CJ in 100-150 randomly selected mitochondria from each experimental condition. Data represent average ± SEM of four independent experiments. ***p<0.001 in a paired sample Student's t-test between EV- and CRE-(EV); ### p<0.001 in a paired sample Student's t-test between EV- and CRE-(+MIC60); **p<0.01 in a paired sample Student's t-test between EV-(EV) and -(+MIC60); ## p<0.01 in a paired sample Student's t-test between EV-(EV) and CRE-(+MIC60).
(C) Protein levels of MIC60 after MIC60 overexpression in wt and *Opa1*^{tg} MAFs. Equal amounts of protein samples (25 μg) were separated in a Tris-Acetate 3%-8% gel and immunoblotted with the indicated antibodies.
(D) Percentage of cristae with CJ in 100-150 randomly selected mitochondria from each experimental condition. Data represent average ± SEM of four independent experiments. ***p<0.001 in one-way ANOVA between wt- and *Opa1*^{tg}-(EV) or *Opa1*^{tg}-(+MIC60); ### p<0.001 in a paired sample Student's t-test between wt-(EV) and -(+MIC60).

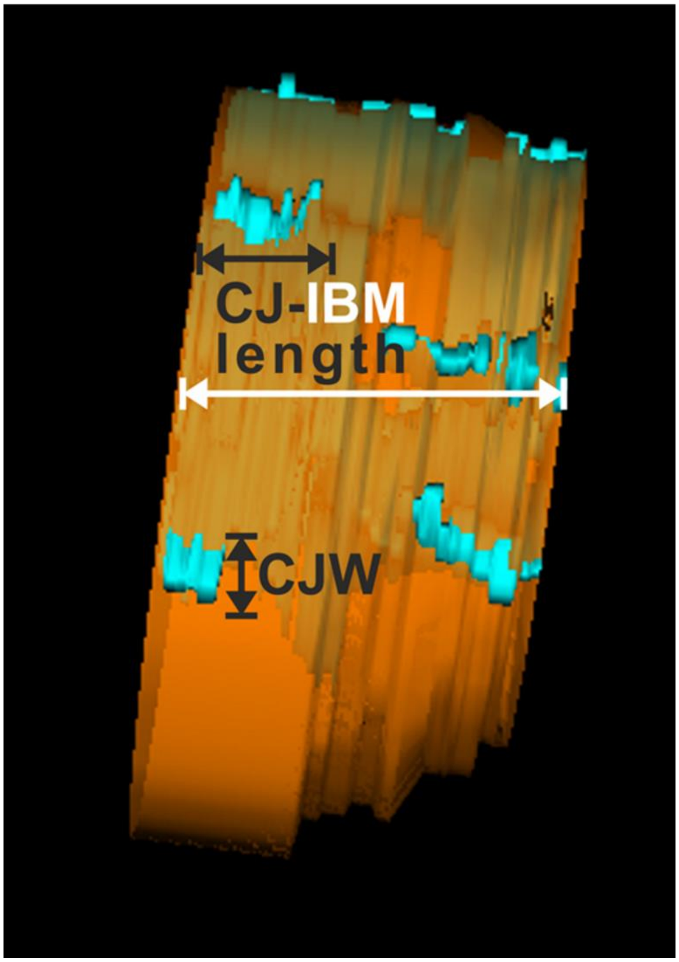

Figure S5. CJ parameters measurements on a 3D tomographic reconstruction of a mitochondrion. Related to Figure 5.
Inner membrane is depicted in orange and cristae junction in cyan. Outer membrane has been peeled out to highlight CJ. The length of the inner boundary membrane (IBM) is denoted with a white double-edged arrow. Cristae junction width (CJW) and cristae junction-inner boundary membrane length (CJ-IBM length) are denoted with black double-edged arrows. The CJW corresponds to the length of the vertical line that defines the CJ in the different frames. CJ-IBM length corresponds to the length of the horizontal line that defines the presence of the same CJ over successive tomography frames. Details in their quantification are described in *Experimental procedures section.*

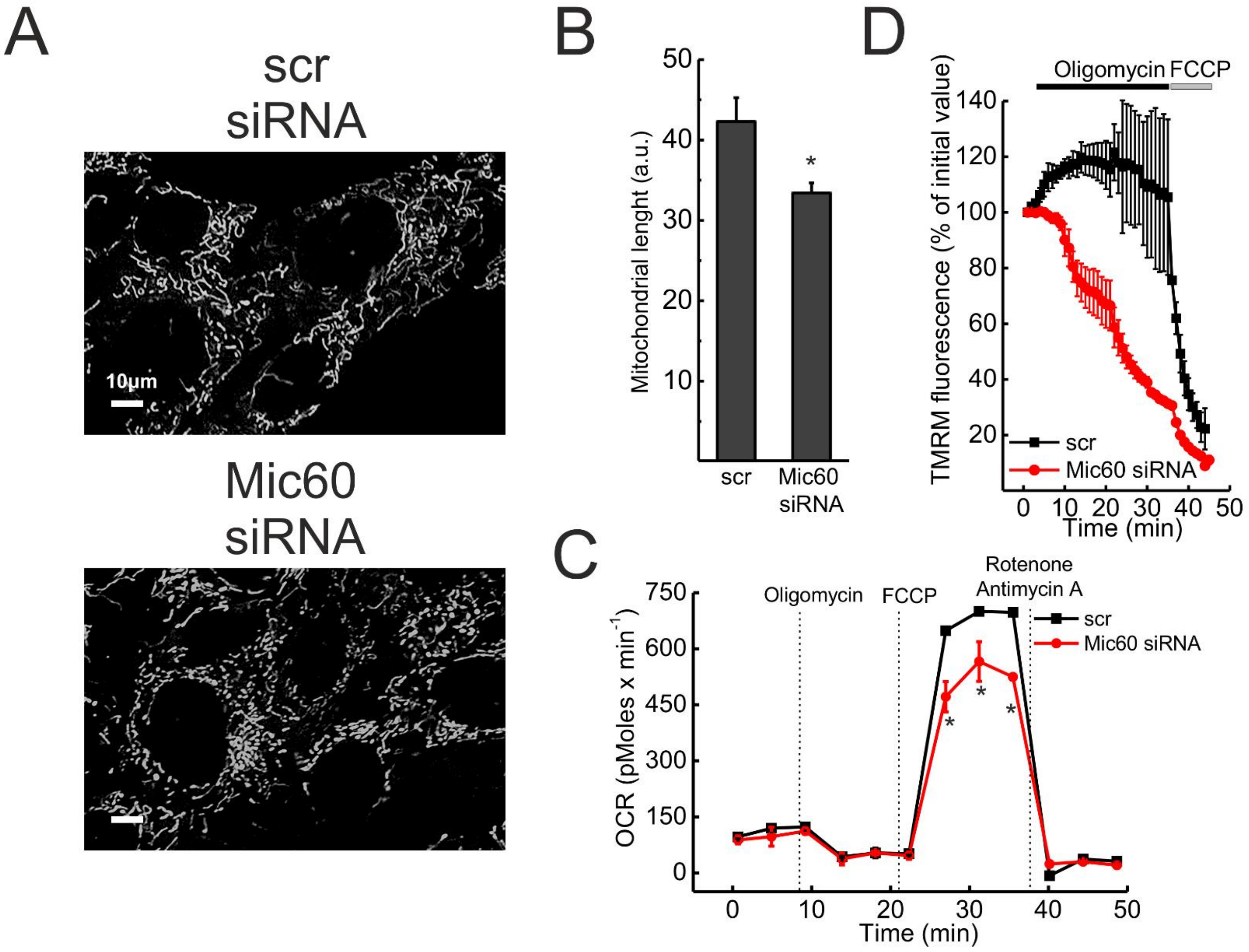

Figure S6. *Mic60* silencing leads to mitochondrial dysfunction. Related to Figure 6.

(A) Representative confocal images of MAFs transfected with scramble (scr) or Mic60 siRNA (#3) and labelled with tetramethylrhodamine methyl ester (TMRM). Scale bar: 10 µm.

(B) Average mitochondrial major axis length. Experiments were as in (A). Data represent average ± SEM of three independent experiments (six mitochondria per cell, at least 30 cells/experiment). *p<0.05 in a paired sample Student's t-test.

(C) Seahorse analysis of MAFs upon *Mic60* silencing (Mic60 siRNA #1-3). Oxygen consumption rate was measured in basal conditions and after the addition of oligomycin 1.3 µM, FCCP 0.4 µM and Rotenone 1 µM/Antimycin A 1.2 µM. Data represent average ± SEM of three independent experiments. * p<0.05 in a paired sample Student's t-test.

(D) Mitochondrial membrane potential in MAFs transfected with scramble (scr) or Mic60 siRNA (Mic60 siRNA #1-3) was monitored following TMRM fluorescence change. Where indicated, 2 µM oligomycin and 2 µM FCCP were added. Data represent average ± SEM of four independent experiments.

Supplemental Experimental Procedures. Related to Experimental Procedures

Bioinformatic analysis

The non-redundant protein sequence database at the National Center for Biotechnology Information (NIH, Bethesda) was searched iteratively using the PSI-BLAST program with the yeast and human MIC60 as starting queries. PSI-BLAST was run with expectation value of 0.01 as the cut-off for inclusion of sequences into the position-specific scoring matrix. Multiple alignments of MIC60 homologues were constructed using the MUltiple Sequence Comparison by Log- Expectation program (MUSCLE) and manually adjusted on the basis of the examination of PSI-BLAST search outputs and the superposition of the predicted transmembrane helices. Transmembrane helices were manually predicted and validated using the TMpred, HMMTOP, and TMHMM programs. Protein domains composition and location were detected and analyzed using the Simple Modular Architecture Research Tool program (SMART).

Molecular Biology

pcDNA6.2-hMic60-V5 vector was generated by subcloning the human, transcript variant 1 DNA NM_006839.1 from donor plasmid pCMV6-AC to pDEST-V5, using the Gateway technology (ThermoFisher). Mitochondrially targeted red fluorescent protein (mtRFP), corresponding to pDsRed2-Mito was previously described (Cipolat et al., 2004).
For Mic60 silencing the following siRNAs were used: Stealth Mic60 siRNA#1 (MSS233674, LifeTechnologies), Mic60 siRNA#2 (MSS233683, LifeTechnologies) or Mic60 siRNA#3 (MSS233684, LifeTechnologies). Silencer Negative Control N° 1 siRNA (Ambion, Life technologies).
Recombinant wild type and mutant BID were produced, purified and cleaved with caspase-8, as previously described (Frezza et al., 2006).

Cell culture, Transfection and Infection

Mouse embryonic fibroblasts (MEFs) and wild type (WT), *Opa1*$^{flx/flx}$ and *Opa1*^{tg} SV40 transformed mouse adult fibroblast (MAFs) were cultured as previously described (Cogliati et al., 2013).
For MIC60 overexpression and simultaneous acute *Opa1* ablation, *Opa1*$^{flx/flx}$ MAFs were infected with the indicated adenoviruses, and after 16 hrs co-transfected with mtRFP and pcDNA6.2-hMIC60-V5 (+MIC60) or pcDNA6.2 (EV) using Lipofectamine. After 24 hrs GFP-positive and RFP–positive cells were sorted and subcultured for 16 hrs before being processed for further analysis.
For *Mic60* silencing (Mic60 siRNA) and simultaneous acute *Opa1* ablation, *Opa1*$^{flx/flx}$ MAFs were infected with the indicated adenoviruses, and after 24 hrs cells were transfected with the indicated siRNA. Cells were sorted for GFP-positive cells and processed for further analysis.

Mitochondrial isolation and *in vitro* assays

Mitochondria from mouse adult fibroblasts (MAFs) and mouse liver and heart tissues were isolated as previously described (Frezza et al., 2007).
Mitochondria (1 mg/ml) were resuspended in Experimental Buffer (EB) (Frezza et al., 2007) and treated as detailed in the Figure legends. Cytochrome c redistribution and release assays were performed as previously described (Frezza et al., 2006).

Electrophoresis

For 1st dimension BNGE sample preparation, isolated mitochondria treated as specified in the Figure legends were resuspended in Native solubilization buffer (NB, 50µl) containing Native Sample buffer (Life Technologies), 1.25% digitonin (Life Technologies) and 1:100 protease inhibitor cocktail (PIC, SIGMA). After 10 min incubation on ice, samples were centrifuged at 25000 x *g* at 4°C. Supernatants were collected and 2 µl of 5% Coomassie G-250 (Life Technologies) were added before loading. Electrophoresis was performed in the presence of cathode (dark blue cathode: 45 min; light blue cathode: 90 min) and anode buffers prepared following manufacturer's instructions (Life Technologies) in precast native Bis-Tris 3%-12% gel (Life Technologies). When indicated, protein complexes were blotted onto polyvinylidene difluoride (PVDF) membranes (Millipore) and probed for the indicated antibodies or stained with Coomassie Blue (40% methanol, 10% acetic acid, 0.25 gr Coomassie Brilliant Blue R-250 - Bio-Rad).
2nd dimension BN-BNGE was performed at 125 V for 1.5 hr using dark cathode buffer and at 150 V, overnight with light cathode buffer. During 2D BN-BNGE, complexes resistant to detergent are aligned in a diagonal easily identified by the Coomassie present in the running samples.
To prepare samples for 3rd dimension SDS-GE, the following solutions were used: RSa=1x LDS sample buffer (Life technologies), 4% β-mercaptoethanol; RSb=1x LDS sample buffer, 1% N, N-dimethylacrylamide (DMA, SIGMA) and RSc=1x LDS sample buffer, 20% ethanol and 0.1% β-mercaptoethanol.

BNGE of heart samples was performed in presence of dark cathode at 100 V for 3.5 hrs and bright cathode buffer at 150 V overnight.

Biochemistry

Protein lysis was performed in presence of proteases inhibitor cocktail (PIC, Sigma) for 30 min at 4°C. After centrifugation at maximal speed for 20 min, supernatants were collected and protein concentration was determined using Bradford reagent (Bio-Rad).
The following antibodies were used: anti-MIC60 polyclonal (1:1000, Proteintech), anti-OPA1 monoclonal (1:1000, BD PharMingen), anti-Fp (Flavoprotein subunit of succinate dehydrogenase, complex-II) monoclonal (1:4000, Abcam), anti-MIC19 polyclonal (1:1000, Proteintech), anti-TOM20 (1:2000, Santa Cruz) and anti-ACTIN (1:10000, Millipore) antibodies. Isotype matched secondary antibodies were conjugated to horseradish peroxidase and chemiluminescence was detected with ECL (Amersham) using either traditional methods with films (Amersham) or ImageQuant LAS 4000 (GE Healthcare Life Sciences).
Chemical crosslinking with dithiobis(succinimidyl propionate) (DSP, Pierce) was performed by incubating isolated mitochondria (1 mg/ml) with 2 mM DSP in EB for 30 min. Reaction was quenched with 20 mM Tris-HCl. Following quenching of the BMH or DSP crosslinking reaction, mitochondria were pelleted by centrifugation at 12000 x g, 10 min, 4°C. Pellet were solubilized in Laemmli buffer (LB) 2X, boiled and electrophoretically separated using a 3%-8% Tris-Acetate gel (Life technologies).

Immunoprecipitation

Isolated mitochondria from liver were lysed in 150 mM NaCl, 25 mM Tris-Cl pH 7.4, 1 mM EDTA, 5% glycerol, 0.1% Triton X-100 in the presence of PIC (Sigma). Lysates were precleared with 20 µl of Protein-A agarose beads (Roche) for 30 min at 4°C to avoid unspecific binding. Protein-A agarose beads (30 µl) were incubated with 2.4 µg anti-MIC60 in lysis buffer for 2 hr at 4°C. Subsequently, mitochondrial lysates (250 µg) were incubated with the antibody-coupled beads for 2 hr at room temperature. The immunoprecipitated material was washed once with 250 mM NaCl, 25 mM Tris-Cl pH 7.4, 1 mM EDTA, 0.05% Triton X-100. Bound proteins were eluted in LB 2X with 5% β-mercaptoethanol and 1% SDS, boiled and separated electrophoretically in a Bis-Tris 4%-12% gel (Life technologies).
Isolated mitochondria from mouse embryonic fibroblasts were lysed as described above. Beads were incubated with anti-OPA1 in lysis buffer (2 µg) and after 1 h the mitochondrial lysates (125 µg) were incubated with the pretreated beads for 3 hr. After washing of beads, bound proteins were eluted in LB 2X with 5% β-mercaptoethanol, and after boiling samples were separated electrophoretically in a Bis-Tris 4%-12% gel (Life technologies) and immunoblotted as indicated in figure legend.
For immunoprecipitation after chemical crosslinking with DSP, crosslinked mitochondria were lysed in 150 mM NaCl, 20 mM HEPES/KOH pH 7.4, 1 mM EDTA, 5% glycerol, 0.1% Triton X-100 and centrifuged at 21000 x g for 30 min. Part of the lysates were boiled in LB 1X containing when indicated 5% β-mercaptoethanol and 25 mM DTT to cleave the DSP linker. For immunoprecipitation, beads were incubated with anti-MIC60 in lysis buffer (2.4 µg) and after 1 hr the crosslinked mitochondria lysates (125 µg) were incubated with the pretreated beads for 3 hrs. The boiled samples were separated electrophoretically in a Tris-Acetate 3%-8% gel.

BNGE based semi-quantitative proteomic analysis

For mass spectrometry analysis of mitochondrial heart complexes, complexes solubilized from mitochondria purified from two mouse CD1 hearts as described above were separated by BNGE using a homemade 20x20 cm, 3%-13% acrylamide gel (Schagger, 1995). Three different experiments were loaded in parallel for each experimental condition. Gels were stained using GelCode Blue Stain (Thermo Scientific) and each lane was divided and excised in 25 bands covering the whole electrophoretic run. The same bands from the triplicate samples were pooled in an Eppendorf tube, minced and digested with modified porcine trypsin (Promega) at a final trypsin-protein 1:20 ratio overnight at 37°C in 100 mM ammonium bicarbonate, pH 8.8. The resulting tryptic peptide mixtures were subjected to nano-liquid chromatography coupled to mass spectrometry using a data-independent scanning (DiS) method as described (Guaras et al., 2016). Peptides were injected onto a C-18 reversed phase (RP) nano-column (75 µm I.D. and 50 cm, Acclaim PepMap100, Thermo Scientific) and analyzed in a continuous acetonitrile gradient consisting of 0%-30% B in 240 min, 50-90% B in 3 min (B=90% acetonitrile, 0.5% acetic acid). A flow rate of ca. 200 nL/min was used to elute peptides from the RP nano-column to an emitter nanospray needle for real time ionization and peptide fragmentation on an Orbitrap Fusion mass spectrometer (Thermo Fisher, San José, CA, USA). Protein identification was performed using Sequest running under Proteome Discoverer 1.4.0.288 (Thermo Fisher Scientific), allowing two missed cleavages, and using 3 Da and 20 ppm precursor and fragment mass tolerances, respectively. False discovery rate (FDR) of identification was controlled as described by the novel algorithm Dxtractor (Guaras et al., 2016; Cogliati et al., 2016).

SILAC labeling and proteomics

For SILAC labeling, in experiment “a” light mitochondria were treated with 20 pmol/mg cBID in EB for 30 min, 25°C, and heavy mitochondria were untreated. After centrifugation at 10000 x g, 10 min, 4°C, the pellets from both samples were mixed and resuspended together in NB to extract the complexes. In experiment “b”, light mitochondria were untreated while heavy mitochondria were treated with 20 pmol/mg cBID^{KKAA} in EB for 30 min, 25°C. After centrifugation the pellets were mixed and processed as in experiment “a”.

Protein complexes from experiments “a” and “b” were loaded onto parallel lanes and complexes were separated by BNGE. Three lanes from experiment “a” and “b” were stained with Coomassie and bands between 500 and 1000 kDa were excised with a scalpel and digested with trypsin. Digestion proceeded overnight at 37°C in 100 mM ammonium bicarbonate, pH 8.8. The resulting tryptic peptide mixtures were subjected to nano-liquid chromatography coupled to tandem mass spectrometry (LC-MS) for protein identification. Peptides were injected onto a C-18 reversed phase (RP) nano-column (75 μm I.D. and 50 cm, Acclaim PepMap100, Thermo Scientific) and analyzed in a continuous acetonitrile gradient consisting of 0-30% B in 240 min, 50-90% B in 3 min (B=90% acetonitrile, 0.5% acetic acid). A flow rate of ca. 200 nL/min was used to elute peptides from the RP nano-column to an emitter nanospray needle for real time ionization and peptide fragmentation on a Q Exactive mass spectrometer (Thermo Fisher, San José, CA, USA). Protein identification was performed using Sequest running under Proteome Discoverer 1.4.0.288 (Thermo Fisher Scientific). Peptide quantification was performed as described (Lopez-Ferrer et al., 2006; Ramos-Fernandez et al., 2007; Bonzon-Kulichenko et al., 2011), using QuiXoT. Proteins identified with only one peptide or peptides identified with only one PSM were removed from the data. Isotopic envelope of ion parents produced by the superimposition of unlabeled and singly labeled peptides was determined by least-squares fitting to spectrum peaks, bycalculating isotopic distributions from the peptide mass as described previously (Lopez-Ferrer et al., 2005). Statistical analysis took into account four different sources of variance at the spectrum-fitting, scan, peptide, and protein levels (Jorge et al., 2009).

Virus production and infection

The retroviral expression vector pMSCV-IRES-GFP containing truncated Bid (pMIG-tBid) was used to generate amphotropic retroviruses expressing tBID as described previously (Cogliati et al., 2013). The efficiency of transduction was typically around 80%-90% as determined by counting GFP positive cells with an epifluorescence microscope (data not shown). Experiments were performed 48 hrs post-infection on sorted GFP–positive cells.

Apoptosis detection

For cell death detection, 4 x 10^5 *Opa1*$^{flx/flx}$ MAFs were grown in 12-well plates, transfected with Mic60 siRNA and after. 48 hr treated with 2 μM Staurosporine (STS). After 6 hr cells were harvested and stained with Annexin-V-Alexa 568 (eBiosciences). Cell death was measured by flow cytometry (FACSCalibur, BD Biosciences) as the percentage of the Annexin-V-positive events in the total population.

Imaging

For imaging of mitochondrial network, cells (3.5 x 10^5) were seeded on 13 mm round glass coverslips, transfected as indicated and after 48 hr incubated with 5 nM Tetramethylrhodamine methyl ester (TMRM) in presence of 2 μM Cyclosporine H (CsH) in Hank’s Balanced Salt Solution (HBSS) supplemented with 10 mM Hepes. Fluorescence signals were analyzed using IMIC Andromeda system (Fondis Electronic) equipped with ORCA-03G Camera (Hamamatsu), a 60X oil objective (UPLAN 60X, 1.35NA, Olympus), a 561 nm laser for excitation and a FF01-446/523/600/677 emission filter (Semrock). Measurement of mitochondrial major axis length was performed in at least six mitochondria per cell, in a minimum of 30 cells/experiment (180 mitochondria/experiment). Mitochondrial length was then quantified using Image J tool ‘Freehand line selection’.
For imaging of mitochondrial membrane potential, 7 x 10^5 cells seeded on 24 mm round glass coverslips were incubated with 5 nM TMRM in presence of 2 μM CsH in HBSS supplemented with 10 mM Hepes for 30 min at 37°C. TMRM fluorescence was followed in time by acquisition of sequential images every minute with an Olympus IMT-2 inverted microscope equipped with a CellR Imaging system. Where indicated Oligomycin (2μM) or Carbonyl cyanide-4-(trifluoromethoxy)phenylhydrazone (FCCP, 2 μM) were added.

Analysis of mitochondrial respiration

Oxygen consumption rate (OCR) was measured with the XF24 Extracellular Flux Analyzer (Seahorse Bioscience, Billerica, MA, USA). MAFs were seeded in XF24 cell culture microplates at 6 × 10^4 cells/well with complete DMEM. After 24 hrs cells were transfected with Mic60 siRNA (#1-3) or scramble oligos and maintained 36-48 hrs in 0.2 ml complete medium at 37°C in 5% CO_2. Experiments were carried out on confluent monolayers and the measurements were initiated after replacing the medium with Cellular Assay Solution (8.3 g/L DMEM, 5 g/L Glucose, 0.58 g/L Glutamine, 1 mM Sodium Pyruvate, 0.015 g/L Phenol Red, pH 7.2). Cells were incubated at 37°C for 30 min to allow temperature and pH equilibration. A titration with FCCP was performed to determine the optimal FCCP concentration. After three measurements of basal OCR each 5 min, 70 μl of a solution containing oligomycin, FCCP or rotenone plus antimycin A were sequentially added to each well to reach final concentrations of 1.3 μM oligomycin, 0.4 μM FCCP and 1 μM for rotenone and 1.2 μM antimycin A. Data are expressed as pmol of O_2 per minute. Three measurements were performed after each compound injection. The analysis was performed simultaneously in the same plate for the control and *Mic60* knockdown cells.

Electron Microscopy and Tomography

Cells were fixed and processed for electron microscopy analysis 48 hrs after transfection. When indicated, cell sorting (FACS Canto, BD Biosciences) was performed 24 hr post-infection to select GFP or/and RFP positive cells, which were subsequently seeded and fixed 48 hr post-infection or as specified. Cell fixation was performed for 1 hr at 37°C with freshly prepared 1.25% (V/V) glutaraldehyde in 0.1 M sodium cacodylate, pH 7.4. After washing with 0.1 M sodium cacodylate, cells were post-fixed in 1% OsO_4, 1.5% $K_4Fe(CN)_6$ in 0.1 M sodium cacodylate pH 7.4, stained with 0.5% uranyl acetate, dehydrated in ethanol and embedded in Embed 812. Thin sections were imaged on a Tecnai-12 electron microscope (Philips-FEI) at the EM Facility of University of Padua.
Electron tomography was performed as previously described (Frezza et al., 2006) on 200-250 nm thick sections collected on Formvar-coated copper slot grids with gold fiducials (10 nm) applied on both surfaces of the grids imaged in a 200 kV Tecnai G2 20 electron microscope (FEI, Eindhoven, The Netherlands) at magnification of 25k or 29k resulting in a pixel's size of 0.92 nm and 0.77 nm. Tilted images (+65/-65 according to a Saxton scheme with a starting angle of 1.5°) were acquired using Xplorer 3D (FEI, Eindhoven, The Netherlands) with an Eagle 2k×2k CCD camera (FEI, Eindhoven, The Netherlands). Tilted series alignment and tomography reconstruction was performed using IMOD (Mastronarde, 1997) and segmentation and 3D rendering using Reconstruct (Fiala, 2005). Measurements were performed using ImageJ and the z scale was stretched using a 1.6 factor to correct for resin shrinkage (Mastronarde, 1997).
Number of frames reconstructed in the tomograms of Figure 5 were as following: EV- scr/EV: 72f; EV- Mic60 siRNA: 98f; EV-(+MIC60): 62f; CRE- scr/EV: 98f; CRE- Mic60 siRNA: 120f; CRE-(+MIC60): 108f; $Opa1^{tg}$-scr/EV: 98f; $Opa1^{tg}$-Mic60 siRNA:110f; $Opa1^{tg}$-(+MIC60):110f.

Supplemental References

Bonzon-Kulichenko, E., Pérez-Hernández, D., Núñez, E., Martínez-Acedo, P., Navarro, P., Trevisan-Herraz, M., Ramos Mdel, C., Sierra, S., Martínez-Martínez, S., Ruiz-Meana, M., Miró-Casas, E., García-Dorado, D., Redondo, J.M., Burgos, J.S., Vázquez, J. (2011). A robust method for quantitative high-throughput analysis of proteomes by 18O labeling. Mol Cell Proteomics *10*, M110.003335.

Cogliati, S., Calvo, E., Loureiro, M., Guaras, A.M., Nieto-Arellano, R., Garcia-Poyatos, C., Ezkurdia, I., Mercader, N., Vázquez, J., Enriquez, J.A. (2016). Mechanism of super-assembly ofrespiratory complexes III and IV. Nature. DOI: 10.1038/nature20157.

Fiala, J.C. (2005). Reconstruct, a free editor for serial section microscopy. J Microsc. *218*, 52-61.

Guarás, A., Perales-Clemente, E., Calvo, E., Acín-Pérez, R., Loureiro-Lopez, M., Pujol, C., Martínez-Carrascoso, I., Nuñez, E., García-Marqués, F., Rodríguez-Hernández, M.A., *et al*. (2016). The CoQH2/CoQ ratio serves as a sensor of respiratory chain efficiency. Cell Rep. *15*, 197-209.

Jorge, I., Navarro, P., Martínez-Acedo, P., Núñez, E., Serrano, H., Alfranca, A., Redondo, J.M., Vázquez, J. (2009). Statistical model to analyze quantitative proteomics data obtained by 18O/16O labeling and linear ion trap mass spectrometry, application to the study of vascular endothelial growth factor-induced angiogenesis in endothelial cells. Mol Cell Proteomics *8*, 1130-49.

López-Ferrer, D., Capelo, J.L., Vázquez, J. (2005). Ultra fast trypsin digestion of proteins by high intensity focused ultrasound. J Proteome Res. *4*, 1569-74.

López-Ferrer, D., Ramos-Fernández, A., Martínez-Bartolomé, S., García-Ruiz, P., Vázquez, J. (2006). Quantitative proteomics using 16O/18O labeling and linear ion trap mass spectrometry. Proteomics *6*, S4-11.

Mastronarde, DN. (1997). Dual-axis tomography, an approach with alignment methods that preserve resolution. J Struct Biol. *120*, 343-52.

Ramos-Fernández, A., López-Ferrer, D., Vázquez, J. (2007). Improved method for differential expression proteomics using trypsin-catalyzed 18O labeling with a correction for labeling efficiency. Mol Cell Proteomics *6*, 1274-86.

Schägger, H. (1995). Quantification of oxidative phosphorylation enzymes after blue native electrophoresis and two-dimensional resolution, normal complex I protein amounts in Parkinson's disease conflict with reduced catalytic activities. Electrophoresis *16*, 763-70.
