# Supplementary material for: Optic Atrophy 1 Is Epistatic to the Core MICOS Component MIC60 in Mitochondrial Cristae Shape Control
Source: Cell Rep. 2016 Dec 13;17(11):3024–34. doi: 10.1016/j.celrep.2016.11.049 (PMC5186903; doi:10.1016/j.celrep.2016.11.049)
Supplement: Document S2. Article plus Supplemental Information [file mmc2.pdf]

Cell Reports

Article

Optic Atrophy 1 Is Epistatic to the Core MICOS Component MIC60 in Mitochondrial Cristae Shape Control

Graphical Abstract

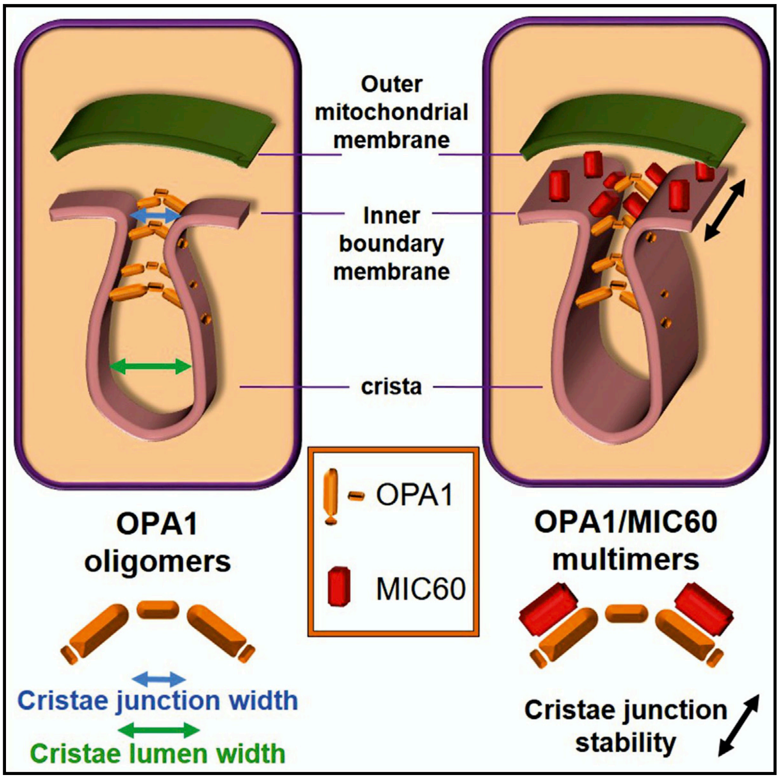

Highlights

- Complexes containing OPA1 and MIC60 are targeted during cristae remodeling
- OPA1 lies upstream of MIC60 in regulating cristae junction number and stability
- OPA1 is the sole regulator of cristae junction width

Authors

Christina Glytsou, Enrique Calvo, Sara Cogliati, ..., Jose Antonio Enriquez, Luca Scorrano, Maria Eugenia Soriano

Correspondence

luca.scorrano@unipd.it (L.S.), mariaeugenia.soriano@unipd.it (M.E.S.)

In Brief

Mitochondrial ultrastructure dictates mitochondrial function and cell viability. Glytsou et al. find that Optic Atrophy 1 (OPA1) and the MICOS component MIC60 interact in cristae shape regulation and place OPA1 epistatic to MIC60 in cristae biogenesis and remodeling, providing a unifying molecular model of cristae morphology control.

Glytsou et al., 2016, Cell Reports *17*, 3024–3034
December 13, 2016 © 2016 The Author(s).
http://dx.doi.org/10.1016/j.celrep.2016.11.049

OPEN ACCESS
CellPress

Cell Reports
Article

Optic Atrophy 1 Is Epistatic to the Core MICOS Component MIC60 in Mitochondrial Cristae Shape Control

Christina Glytsou,[1,2,3] Enrique Calvo,[4] Sara Cogliati,[1,4] Arpit Mehrotra,[1] Irene Anastasia,[1] Giovanni Rigoni,[1] Andrea Raimondi,[5] Norihito Shintani,[1,3,6] Marta Loureiro,[4] Jesùs Vazquez,[4] Luca Pellegrini,[7] Jose Antonio Enriquez,[4] Luca Scorrano,[1,3,8,*] and Maria Eugenia Soriano[1,3,*]
[1]Department of Biology, University of Padova, Padova 35121, Italy
[2]IRCCS Fondazione Santa Lucia, Rome 00179, Italy
[3]Dulbecco-Telethon Institute, Venetian Institute of Molecular Medicine, Padova 35129, Italy
[4]Centro Nacional de Investigaciones Cardiovasculares Carlos III, Madrid 28029, Spain
[5]Centro Imaging Sperimentale, IRCCS Istituto Scientifico San Raffaele, Milano 20132, Italy
[6]Graduate School of Pharmaceutical Sciences, Osaka University, Osaka 565-0871, Japan
[7]Department of Molecular Biology, Medical Biochemistry and Pathology, Université Laval, Quebec G1J 2G3, Canada
[8]Lead Contact
*Correspondence: luca.scorrano@unipd.it (L.S.), mariaeugenia.soriano@unipd.it (M.E.S.)
http://dx.doi.org/10.1016/j.celrep.2016.11.049

SUMMARY

The mitochondrial contact site and cristae organizing system (MICOS) and Optic atrophy 1 (OPA1) control cristae shape, thus affecting mitochondrial function and apoptosis. Whether and how they physically and functionally interact is unclear. Here, we provide evidence that OPA1 is epistatic to MICOS in the regulation of cristae shape. Proteomic analysis identifies multiple MICOS components in native OPA1-containing high molecular weight complexes disrupted during cristae remodeling. MIC60, a core MICOS protein, physically interacts with OPA1, and together, they control cristae junction number and stability, OPA1 being epistatic to MIC60. OPA1 defines cristae width and junction diameter independently of MIC60. Our combination of proteomics, biochemistry, genetics, and electron tomography provides a unifying model for mammalian cristae biogenesis by OPA1 and MICOS.

INTRODUCTION

The pleotropic roles of mitochondria in energy conversion, cell death, calcium homeostasis, intermediary metabolism, cell differentiation, and even immunity are matched by their morphological and ultrastructural complexity (Pernas and Scorrano, 2016). Mitochondria are organized into five sub-compartments: outer membrane (OMM), intermembrane space (IMS), inner boundary membrane (IBM), cristae, and matrix (Cogliati et al., 2016). This organization allows sub-compartmentalization of biochemical processes. For instance, cristae host the oxidative phosphorylation components, including cytochrome *c*, an essential cofactor of effector caspases activation during apoptosis (Pernas and Scorrano, 2016), and allow their assembly into respiration-competent complexes (RCCs) and respiration-competent super-complexes (RCSs) (Acin-Perez and Enriquez, 2014; Acín-Pérez et al., 2008; Lapuente-Brun et al., 2013). Therefore, cristae dictate organelle bioenergetic capacity (Hackenbrock, 1966), a concept validated in vivo using mouse genetic models (Cogliati et al., 2013; Varanita et al., 2015), and participate in apoptosis by controlling the complete cytochrome *c* release (Scorrano et al., 2002).

Functional and electron tomography (ET) studies revealed a high degree of structural complexity of the cristae sub-compartment at the level of both the cristae lumen (CL, the space inside the cristae invagination) and the cristae junction (CJ, the opening of the cristae into the IBM) (Frey and Mannella, 2000). Width is a critical functional parameter of both CL and CJ: the cristae lumen width (CLW), i.e., the distance between the opposing faces of the cristae membrane, controls RCSs assembly and mitochondrial respiratory efficiency (Cogliati et al., 2013). The cristae junction width (CJW) measures 20–40 nm and regulates metabolite diffusion and respiratory chain component segregation between the inner and the cristae membranes (Gilkerson et al., 2003). In addition, CJW increases during apoptosis, allowing cytochrome *c* redistribution from the lumen to the IMS (Große et al., 2016; Scorrano et al., 2002; Yamaguchi et al., 2008). Both CLW and CJW are controlled by the dynamin guanosine triphosphatase (GTPase) OPA1 (Cogliati et al., 2013; Frezza et al., 2006), independent of its role in mitochondrial fusion (Cipolat et al., 2004; Frezza et al., 2006). The direct relationship among CJW, CLW, and OPA1 affects cell death in vitro (Corrado et al., 2016; Costa et al., 2010; Jiang et al., 2014; Landes et al., 2010; Yamaguchi et al., 2008) and in vivo (Civiletto et al., 2015; Varanita et al., 2015). Molecularly, increased apoptotic CLW and CJW correlates with destabilization of ~720 kDa OPA1-containing complexes of unknown composition (Cogliati et al., 2013; Frezza et al., 2006; Varanita et al., 2015).

In addition to OPA1, a large complex, the mitochondrial contact site and cristae organizing system (MICOS) regulates CJ biogenesis in multiple organisms (Herrmann, 2011; Huynen

 © 2016 The Author(s).
This is an open access article under the CC BY license (http://creativecommons.org/licenses/by/4.0/).

et al., 2016; Jans et al., 2013; John et al., 2005; van der Laan et al., 2016; Zerbes et al., 2012). In yeast, MICOS can be subdivided into a sub-complex formed by Mic27, Mic10, and Mic12 and a second one comprising Mic60 (mitofilin) and Mic19 (CHCHD3). Mic19 connects the two sub-complexes (Friedman et al., 2015). This picture is less clear in mammals: first, a homolog of Mic12 has not been yet identified; second, the existence of these two sub-complexes remains unexplored (Huynen et al., 2016); third, silencing of the mammalian homolog of yeast Mic10 does not alter cristae shape, whereas in yeast, its deletion results in a thylakoid-like cristae morphology (Alkhaja et al., 2012; Barbot et al., 2015; Bohnert et al., 2015). Thus, MICOS complex composition and regulation may have changed during vertebrate evolution, perhaps to accommodate its recruitment into the process of apoptotic cristae remodeling. The intriguing possibility that MIC60 and more generally the MICOS complex interact with OPA1 in CLW and CJW regulation has not been substantiated by the retrieval of OPA1 or of its yeast homolog Mgm1p in the MICOS complex so far.

Here we set out to investigate whether MICOS and OPA1 interact in cristae biogenesis and remodeling. A combination of proteomics and biochemistry indicates that mammalian MIC60 interacts with OPA1 in high molecular weight complexes (HMWCs) eliminated during cristae remodeling. An epistasis analysis places OPA1 upstream of MIC60 in the same pathway controlling CJ stability; conversely, OPA1 defines CLW and CJW independently of MIC60. Accordingly, MIC60 does not regulate apoptotic cristae remodeling and cytochrome *c* redistribution. Our results provide a unifying model for mammalian cristae biogenesis and apoptotic remodeling.

RESULTS

MIC60 and OPA1 Are Retrieved in the Same Complexes Targeted during Cristae Remodeling

Cytochrome *c* is a key activator of programmed cell death in vertebrates, possibly explaining the recruitment of cristae remodeling and CJ proteins like OPA1 in this process at the onset of vertebrate evolution. Yeast and mammalian MIC60 form high molecular weight complexes (HMWCs) (Guarani et al., 2015; Li et al., 2016), but whether and how its core component MIC60, conserved in all ancestral mitochondrial precursors from which cristae are retrieved (Muñoz-Gómez et al., 2015a), changes during physiopathological processes remains unknown. We therefore decided to address whether mammalian MIC60 HMWCs change during apoptotic cristae remodeling, when OPA1-containing HMWCs (~720 kDa) are disassembled (Cogliati et al., 2013; Varanita et al., 2015). Blue native gel electrophoresis (BNGE) confirmed that in isolated heart mitochondria, MIC60 and MIC19, the other crucial MICOS component interacting with MIC60 to regulate CJ biogenesis (Darshi et al., 2011; Li et al., 2016; Xie et al., 2007), are retrieved in 600–1,000 kDa HMWCs that partially overlap with the OPA1-containing HMWCs (dashed box in Figure 1A). We next induced apoptotic changes to mitochondria by incubating them with the recombinant caspase-8 cleaved BH3-only BCL-2 family member BID (cBID) or with its cristae remodeling incompetent mutant cBID^{KKAA} (Cogliati et al., 2013). Not only the ~720 kDa OPA1 but also the MIC60-containing complexes running at the same apparent molecular weight (MW) were selectively destabilized during cristae remodeling (dashed box in Figure 1B and quantification in Figure 1C).

To further characterize the fate of the MIC60/MIC19-containing complexes changed during apoptotic-cristae remodeling, we excised and processed for mass spectrometry (MS) analysis protein complexes separated by BNGE from cBID- and cBID^{KKAA}-treated mouse heart mitochondria. A color contour plot representation of the number of spectral counts from MIC60 peptides identified by semiquantitative proteomic analysis in the different HMWCs isolated from normal and apoptotic mitochondria indicated that cBID, but not cBID^{KKAA}, greatly reduced the number of MIC60/MIC19 peptides across the ~720 kDa HMWCs. In these HMWCs, OPA1 levels were also reduced (dashed boxes in Figure 1D). Finally, a quantitative proteomic analysis of mitochondrial complexes isolated after stable isotope labeling with amino acids in cell culture (SILAC) of mouse adult fibroblasts (MAFs) (Figure 1E) confirmed that cBID, but not cBID^{KKAA}, caused a significant loss of a comprehensive set of MIC60/MIC19 peptides in the ~720 kDa MIC60/MIC19-containing HMWCs (Figure 1F). In conclusion, the two critical mammalian MICOS components, MIC60 and MIC19, assemble in HMWCs disrupted during apoptotic cristae remodeling.

Yeast and Mammalian MIC60 Diverge

Because OPA1 and MICOS components are retrieved in the same HMWC targeted during cristae remodeling, we reasoned that OPA1 and MIC60 could physically interact. However, because in yeast the interaction between the OPA1 homolog Mgm1p and the MIC60 homolog Fcj1 does not occur, divergent OPA1 and MIC60 structural features are present in vertebrates to support their physical interaction. A bioinformatic analysis indicated that the transmembrane helices (TMHs) of mammalian and yeast MIC60 are highly divergent (Figures S1A–S1C). This finding is not unexpected, because TMHs are typically poorly conserved; however, whereas the TMH of yeast MIC60 can form a highly structured hydrophobic α helix, that of mammalian MIC60 is predicted to be poorly structured and hydrophobic, mostly because it contains two clusters of glycine residues (Figure S1B) that could facilitate the mobility and dynamic MIC60 interaction with a variety of proteins. Furthermore, MIC60 TMH is strictly conserved in vertebrates, indicating that it was subjected to structural constraints not only linked to its membrane-anchoring function. We also found motifs strictly conserved in all vertebrates absent in yeast Mic60 (Figure S1C): these sequences span the middle mammalian MIC60 and do not display obvious homology with any other mouse and human sequence, suggesting again strong purifying selection during vertebrate evolution. In conclusion, whereas the C-terminal domain is conserved in all eukaryotic orthologs of MIC60 (Muñoz-Gómez et al., 2015a, 2015b), novel TMH and N-terminal domains emerged at the outset of vertebrate evolution, possibly to recruit MIC60 into a pathway-like apoptosis or to confer new partners and mechanisms of regulation.

OPA1 and MIC60 Interact Physically

Comforted by the results of the bioinformatics analysis, we decided to verify in the wet lab the possibility of an OPA1-MIC60

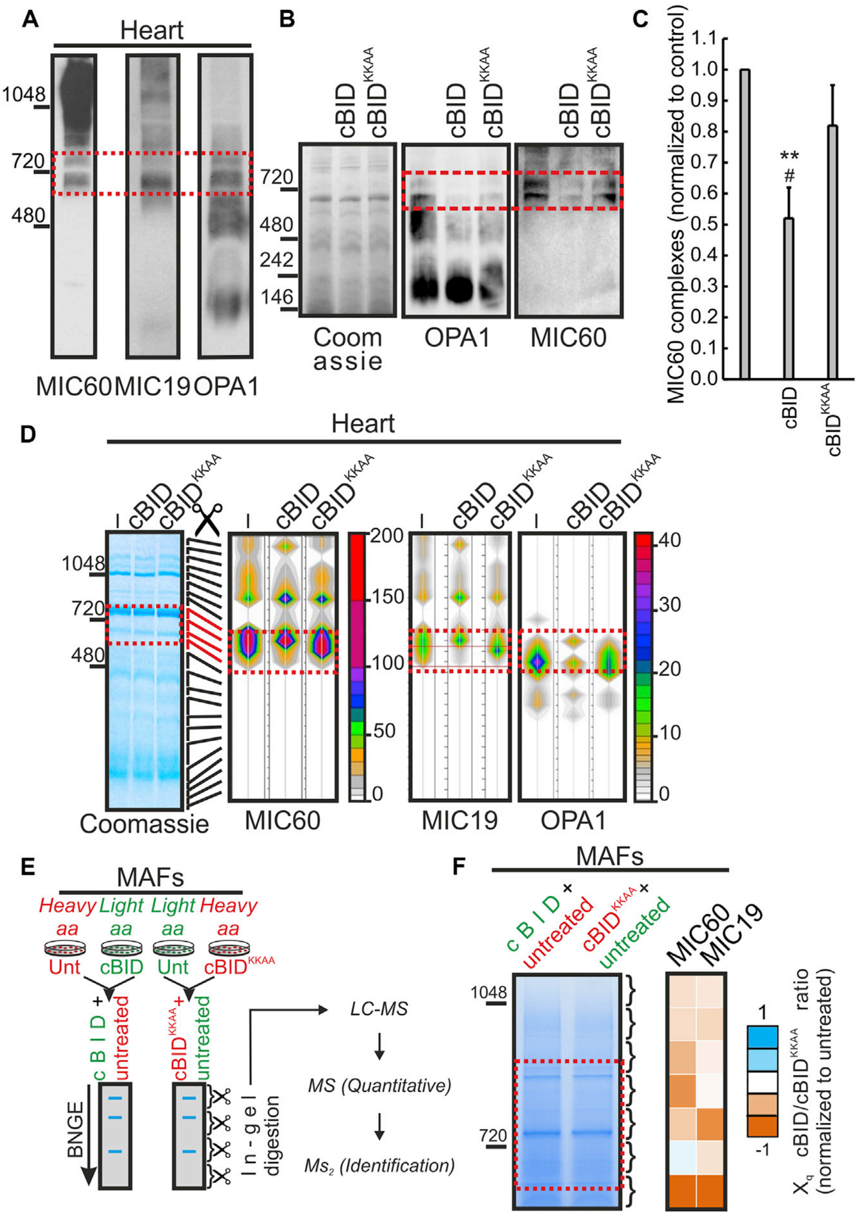

Figure 1. MIC60-Containing Complexes Are Disrupted during Cristae Remodeling

(A) Complexes extracted from mouse heart mitochondria were separated by blue native (BN)-PAGE, transferred onto a PVDF membrane, and probed with the indicated antibodies. Boxed area: common OPA1 and MIC60 complexes.

(B) Protein complexes extracted from mouse heart mitochondria treated as indicated were separated by BNGE, Coomassie stained or transferred onto PVDF membranes, and probed using the indicated antibodies.

(C) Quantitative densitometric analysis of MIC60 complex levels in experiments as in (B). Data represent mean ± SEM of three independent experiments. $^{**}p < 0.01$ in a paired sample Student's t test between cBID and untreated; $\#p < 0.05$ between cBID and cBID^{KKAA}.

(D) Mass spectrometry analysis of MIC60, MIC19, and OPA1 in HMWCs. Heart mitochondria were treated for 20 min as indicated, and extracted complexes were separated by BNGE and Coomassie stained (left). The indicated gel bands were sliced, processed, and analyzed by MS. Color contour plots (right) indicate the number of spectral counts corresponding to MIC60, MIC19, or OPA1 peptides along the BNGE.

(E) Schematic representation of the SILAC experiments. One experiment (left) contains complexes extracted from control (Unt) and cBID-treated mitochondria isolated from mouse adult fibroblasts (MAFs) grown in a medium containing heavy and light amino acids, respectively. A second experiment (right) contains complexes extracted from control (Unt, light) and cBID^{KKAA}-treated (heavy) mitochondria. Mixed mitochondrial complexes were separated in native conditions, and the obtained gel bands from 500 to 1,048 kDa were excised. A pool of triplicates for each experiment was analyzed by qualitative and quantitative MS.

(F) Heatmap of the ratios of MIC60 peptides in cBID-treated versus cBID^{KKAA}-treated samples from three independent SILAC experiments as in (E). Orange, decrease; white, no change; blue, increase.

interaction. First, we found that a fraction of OPA1 co-immunoprecipitated with MIC60 (Figure 2A) and reciprocally that MIC60 co-immunoprecipitated with OPA1 (Figure 2B). To address whether this interaction also occurred in native complexes, we turned to three-dimensional (3D) blue native-blue native-SDS gel electrophoresis (BN-BN-SDS-GE) that allows the identification of proteins coexisting in detergent-resistant complexes (Figure 2C). Immunoblotting of 3D SDS gel electrophoresis (SDS-GE) showed that MIC60 was retrieved in the ~720 kDa OPA1-containing complex targeted during cristae remodeling (Figure 2D). Moreover, the long-arm, homo-bifunctional, maleimide crosslinker bismaleimidohexane (BMH) stabilized a ~180–190 kDa complex immunoreactive for both OPA1 (MW ≈ 90–100 kDa) and MIC60 (MW ≈ 90 kDa) that was also reduced in apoptotic cBID-treated mitochondria (Figure 2E). This OPA1- and MIC60-immunoreactive adduct was also observed when we crosslinked proteins using the primary amine's long-arm cleavable crosslinker dithiobis(succinimidyl propionate) (DSP) (Figure S2A), suggesting that its formation does not depend on the crosslinker used. Finally, the amount of OPA1 co-immunoprecipitated with MIC60 was greater in these DSP-treated mitochondria (Figure S2B), substantiating that crosslinking stabilizes a specific MIC60-OPA1 interaction. To verify that the crosslinked adduct was specifically formed by OPA1 and MIC60, we inspected its stability following deletion of either OPA1 or MIC60. Upon adenoviral CRE recombinase delivery to *Opa1*$^{flx/flx}$ mouse adult fibroblasts (MAFs) (Cogliati et al., 2013), *Opa1* was successfully deleted (Figure S2C) and MIC60 was no longer retrieved in the crosslinked ~180–190 kDa complex

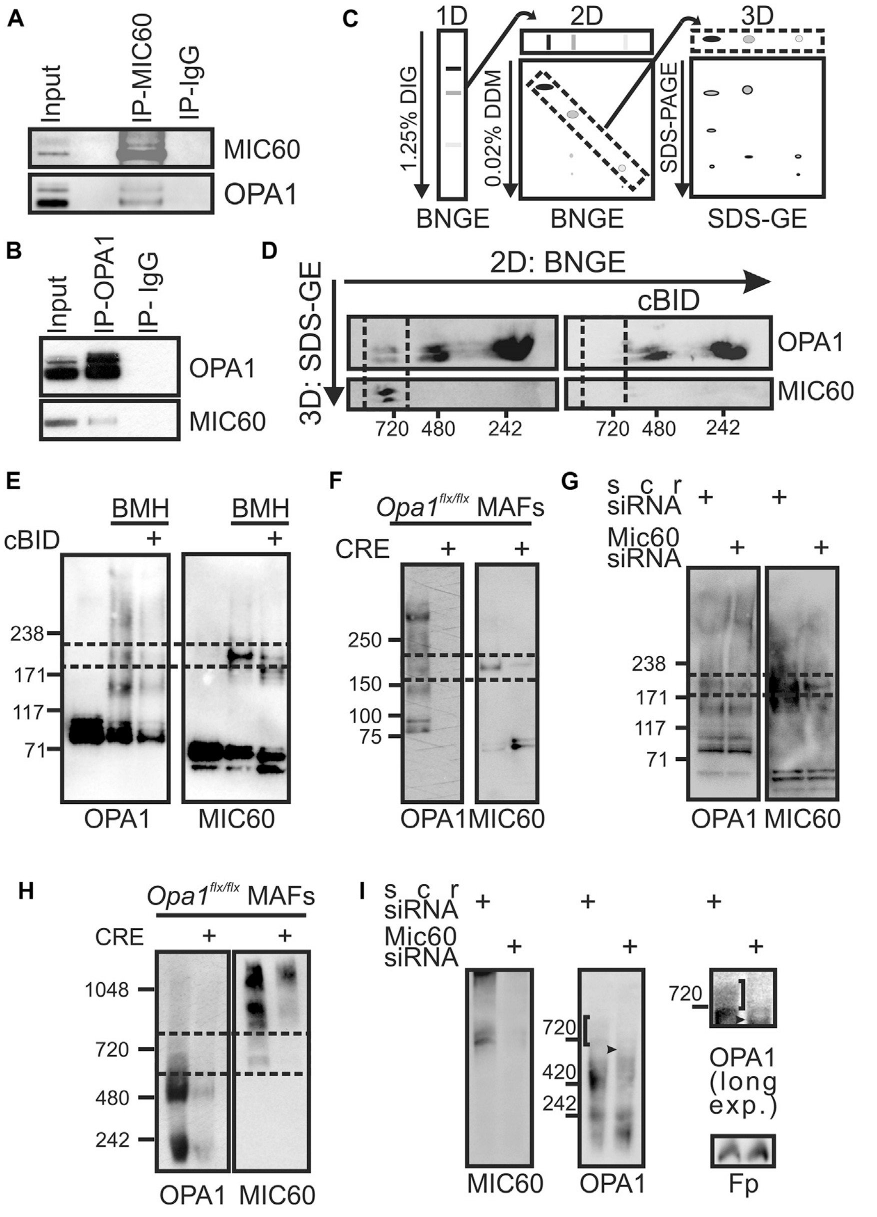

Figure 2. MIC60 and OPA1 Interact in Complexes Disrupted during Cristae Remodeling

(A) Liver mitochondrial lysates (250 μg) were immunoprecipitated with anti-MIC60 coupled to Protein A agarose beads. Bound proteins were separated by SDS-GE and immunoblotted using the indicated antibodies. Input was diluted 1:10.

(B) Mitochondrial lysates from mouse embryonic fibroblast (MEF; 125 μg) were immunoprecipitated with anti-OPA1 coupled to Protein A agarose beads. Bound proteins were separated by SDS-GE and immunoblotted using the indicated antibodies. Input was diluted 1:5.

(C) Schematic representation of 3D BN-BN-SDS-GE analysis of membrane complexes isolated from mouse heart mitochondria.

(D) Western blots using the indicated antibodies of 3D SDS-GE of mouse heart mitochondria treated as indicated.

(E) Equal amounts (25 μg) of protein from MAF mitochondria treated as indicated and crosslinked where indicated (BMH) were separated by SDS-GE and immunoblotted using the indicated antibodies. Boxed area: OPA1-MIC60 adduct.

(F) Mitochondria isolated from *Opa1*$^{flx/flx}$ MAFs infected for 48 hr as indicated were crosslinked with 5 mM BMH. Equal amounts (25 μg) of proteins were separated by SDS-GE and immunoblotted with the indicated antibodies. Boxed area: OPA1-MIC60 adduct. See also Figure S2C.

(G) Equal amounts (25 μg) of mitochondrial protein from MAFs transfected with scramble (scr) or Mic60 siRNA (3) were crosslinked with 5 mM BMH, separated by SDS-GE, and immunoblotted using the indicated antibodies. Boxed area: OPA1-MIC60 adduct. See also Figure S2D.

(H) Protein complexes extracted from mitochondria (150 μg) isolated from *Opa1*$^{flx/flx}$ MAFs infected for 48 hr as indicated were separated by BNGE and immunoblotted using the indicated antibodies. Boxed area: common OPA1 and MIC60 complexes.

(I) Protein complexes extracted from mitochondria (150 μg) isolated from MAFs transfected with scramble (scr) or Mic60 siRNA (3) were separated by BNGE and immunoblotted using the indicated antibodies. Square brackets: OPA1 complex destabilized after *Mic60* silencing. Arrows: lower HMWC containing OPA1.

DIG, digitonin; DDM, n-dodecyl β-D-maltoside; IP, immunoprecipitation; IP-IgG, beads coupled to irrelevant immunoglobulin G (IgG).

(Figure 2F). Similarly, *Mic60* silencing using three different short interfering RNA (siRNA) decreased MIC60 levels (Figure S2D), as well as the amount of OPA1 found in the ~180–190 kDa crosslinked form (Figure 2G). These experiments confirm that the MIC60/OPA1 crosslinked adduct is specific and depends on both OPA1 and MIC60.

We next analyzed whether also the MIC60-OPA1 native ~720 kDa complexes were affected by *Opa1* and *Mic60* ablation. After acute *Opa1* ablation, MIC60 was no longer retrieved in ~720 kDa complexes (Figure 2H). Similarly, in *Mic60*-silenced mitochondria, the ~720 kDa complex was no longer immunoreactive for OPA1 and a new OPA1-positive complex ~600–650 kDa appeared (Figure 2I). In conclusion, these experiments indicate that OPA1 physically interacts with MIC60 and recruits it in a ~720 kDa HMWC: when *Opa1* is deleted, MIC60 is no longer retrieved in this complex, and when MIC60 is silenced, OPA1 is found in a new ~600–650 kDa HMWC.

The Effect of MIC60 on Mammalian Cristae Junctions Requires OPA1

The physical interaction between MIC60 and OPA1 in the ~720 kDa complex that is targeted during cristae remodeling prompted us to investigate their relative role in cristae and CJ

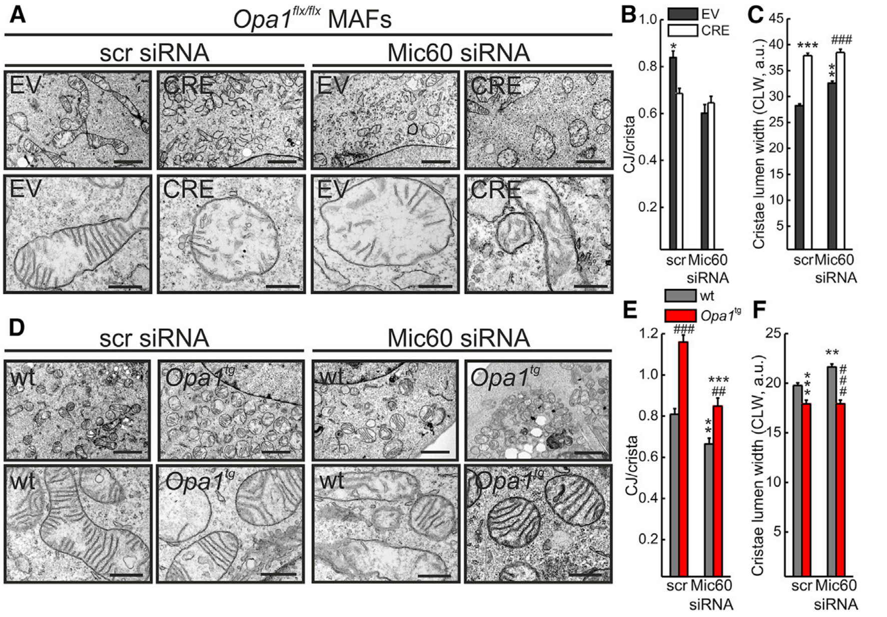

Figure 3. MIC60 and OPA1 Lie in the Same Genetic Pathway Controlling the CJ Number

(A) Representative EM of *Opa1*$^{flx/flx}$ MAFs infected with empty vector-GFP (EV) or Cre recombinase-GFP (CRE) adenoviruses and transfected with scramble (scr) or Mic60 siRNA (2). Scale bars, 2 μm (upper panel) and 500 nm (lower panel). See also Figure S3A.

(B) Quantification of CJ number per crista in experiments as in (A). Data represent mean ± SEM of four independent experiments (n = 100–150 mitochondria per condition). *p < 0.05 in a paired sample Student's t test between EV-scr and all other conditions.

(C) CLW analysis in experiments as in (A). Data represent mean ± SEM of four independent experiments (n = 150–400 cristae per condition). ***p < 0.001 in a paired sample Student's t test between EV-scr and CRE-scr; **p < 0.01 in a paired sample Student's t test between EV-scr and EV-Mic60 siRNA; ###p < 0.001 in a paired sample Student's t test EV-Mic60 siRNA and CRE-Mic60 siRNA.

(D) Representative EM of mitochondria from MAFs of the indicated genotype transfected where indicated using scr or Mic60 siRNA (2). Scale bars, 2 μm (upper panel) and 500 nm (lower panel). See also Figures S3B and S3C.

(E) Quantification of CJ number per crista in experiments as in (D). Data represent mean ± SEM of four independent experiments (n = 100–150 per condition). ###p < 0.001 in one-way ANOVA between wt-scr and *Opa1*^{tg}-scr; ***p < 0.001 in one-way ANOVA between wt-Mic60 siRNA and *Opa1*^{tg}-Mic60 siRNA; **p < 0.01 in a paired sample Student's t test between wt-scr and wt-Mic60 siRNA; ##p < 0.01 in a paired sample Student's t test between *Opa1*^{tg}-scr and *Opa1*^{tg}-Mic60 siRNA.

(F) CLW analysis in experiments as in (D). Data represent mean ± SEM of three independent experiments (n = 130–300 cristae per condition). ***p < 0.001 in one-way ANOVA between WT and *Opa1*^{tg}-scr; ###p < 0.001 in one-way ANOVA between wt-Mic60 siRNA and *Opa1*^{tg}-Mic60 siRNA; **p < 0.01 in a paired sample Student's t test between wt-scr and wt-Mic60 siRNA.

biogenesis. We therefore turned to electron microscopy (EM) and morphometric analysis to perform a genetic epistatic analysis, capitalizing on *Mic60* silencing or overexpression in established models of acute *Opa1* ablation by means of adenoviral CRE delivery to *Opa1*$^{flx/flx}$ MAFs, or permanent, mild, transgene-driven *Opa1* overexpression (*Opa1*^{tg} MAFs). When we efficiently silenced *Mic60* in empty vector (EV)-infected *Opa1*$^{flx/flx}$ MAFs (Figure S3A), the number of CJs per crista (Figures 3A and 3B) and number of cristae with CJ (Figure S3E) were reduced by ~30%, similar to previous reports in yeast and mammals (John et al., 2005; Rabl et al., 2009). However, *Opa1* deletion (Figure S3A) per se induced a significant reduction in the number of CJs per crista (Figures 3A and 3B) and number of cristae with CJ (Figures S3D and S3E). Combined *Mic60* downregulation and *Opa1* ablation induced a quasi-complete reduction in OPA1 and MIC60 levels (Figure S3A) but did not further reduce the number of CJs per crista (Figures 3A and 3B) and number of cristae with CJ (Figure S3E). Thus, OPA1 and MIC60 are components of the same pathway regulating CJ biogenesis. Conversely, *Mic60* silencing induced a minor increase in CLW, which was dramatically increased upon *Opa1* deletion (Figures 3A and 3C). Cristae were not further widened when *Mic60* was ablated following *Opa1* deletion (Figures 3A and 3C), indicating that OPA1 acts as a master regulator of CLW. In *Opa1*^{tg} MAFs, where OPA1 is mildly overexpressed (Figure S3B), the number of CJs per crista (Figures 3D and 3E) and number of cristae with CJ (Figure S3F) were increased; efficient *Mic60* silencing (Figure S3C) reduced both the number of CJs per crista (Figures 3D and 3E) and the number of cristae with CJ (Figure S3F), irrespective of whether OPA1 was overexpressed. Conversely, CLW was narrower in *Opa1*^{tg} MAFs and was not affected by *Mic60* silencing (Figures 3D and 3F), confirming the main role of OPA1 in the regulation of CLW. To relatively position MIC60 and OPA1 in the pathway controlling CJ biogenesis, we completed our epistatic analysis by measuring the same parameters of CJ number and CLW in sorted GFP^{+} and RFP^{+} cells upon infection with the GFP-expressing adenoviruses and MIC60-V5/mtRFP cotransfection. As expected, MIC60-V5 expression in EV-infected *Opa1*$^{flx/flx}$ MAFs (Figure S4A) increased the number of CJs per crista (Figures 4A and 4B) and number of cristae displaying a CJ (Figure S4B), whereas it did not have any effect on CLW (Figures 4A and 4C). The number of CJs per crista was reduced when *Opa1* was deleted; when MIC60-V5 was expressed in *Opa1*-deleted cells (at levels comparable to those achieved in EV-infected *Opa1*$^{flx/flx}$ MAFs) (Figure S4A), the number of CJs per crista increased only marginally, and it did not reach the levels obtained upon MIC60-V5 expression in EV-infected *Opa1*$^{flx/flx}$ MAFs (Figures 4A and 4B). The same picture was observed when we measured the percentage of cristae displaying a CJ (Figure S4B). As expected, MIC60 overexpression was not able to reduce the cristae lumen widening caused by *Opa1* ablation (Figures 4A and 4C). Finally, MIC60 overexpression (Figure S4C) did not further increase the number of CJs per crista (Figures 4D and 4E) and number of cristae displaying a CJ (Figure S4D), nor it did further narrow CLW in *Opa1*^{tg} MAFs (Figures 4D and 4F). In conclusion, OPA1 and MIC60 cooperate to control

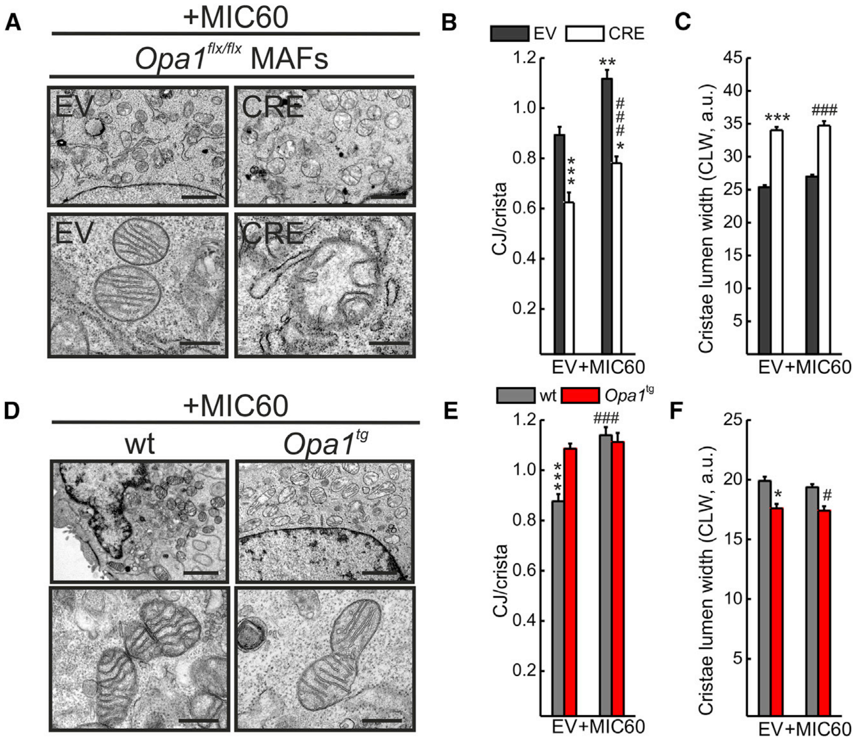

Figure 4. OPA1 Is Epistatic to MIC60 in the Control of the CJ Number

(A) Representative EM of *Opa1*$^{flx/flx}$ MAFs infected and transfected as indicated. Scale bars, 2 μm (upper panel) and 500 nm (lower panel). See also Figure S4A.

(B) Quantification of CJ number per crista in experiments as in (A). Data represent mean ± SEM of four independent experiments (n = 100–150 per condition). ***$p < 0.001$ in a paired sample Student's t test between EV-(EV) and CRE-(EV); **$p < 0.01$ in a paired sample Student's t test between EV-(EV) and EV-(+MIC60); *$p < 0.05$ in a paired sample Student's t test between CRE-(EV) and CRE-(+MIC60); ###$p < 0.001$ in a paired sample Student's t test between EV-(EV) and CRE-(+MIC60).

(C) CLW analysis in experiments as in in (A). Data represent mean ± SEM of four independent experiments (n = 150–250 cristae per condition). ***$p < 0.001$ in a paired sample Student's t test between EV-(EV) and CRE-(EV); ###$p < 0.001$ in a paired sample Student's t test between EV-(+MIC60) and CRE-(+MIC60).

(D) Representative EM of mitochondria from MAFs of the indicated genotype transfected as indicated. Scale bars, 2 μm (upper panel) and 500 nm (lower panel). See also Figure S4C.

(E) Quantification of CJ number per crista in experiments as in (D). Data represent mean ± SEM of four independent experiments (n = 100–150 per condition). ***$p < 0.001$ in one-way ANOVA between wt-(EV) and *Opa1*^{tg}-(EV) or *Opa1*^{tg}-(+MIC60); ###$p < 0.001$ in a paired sample Student's t test between wt-(EV) and wt-(+MIC60).

(F) CLW analysis in experiments as in (D). Data represent mean ± SEM of four independent experiments (n = 100–150 cristae per condition). *$p < 0.05$ in one-way ANOVA between wt-(EV) and *Opa1*^{tg}-(EV); #$p < 0.05$ in a one-way ANOVA between wt-(+MIC60) and *Opa1*^{tg}-(+MIC60).

mammalian CJ biogenesis, OPA1 being placed upstream of MIC60. Conversely, CLW is controlled solely by OPA1.

OPA1 Specifies Cristae Junction Width Independently of MIC60

OPA1 oligomers also control CJW (Figure S5) (Frezza et al., 2006), crucial to limiting cytochrome *c* mobilization from the cristae compartment to the IMS and hence to modulating apoptosis. We therefore wished to understand whether MIC60 cooperated with OPA1 to regulate CJW. To this end, we acquired electron tomograms of mitochondria in the same cellular models employed before for the epistatic analysis of the relative position of OPA1 and MIC60 in CJ biogenesis. Rotations of representative surface that were rendered views of tomographic reconstructions of mitochondria allowed to highlight the individual openings of the CJs (cyan) into the IBM (orange in Figures 5A and S5) and to measure the CJW (Figure S5). Acute *Opa1* ablation increased CJW by ~35%–40% (37.74 ± 1.57 nm in EV-infected *Opa1*$^{flx/flx}$ mitochondria versus 51.82 ± 2.20 nm in CRE-infected *Opa1*$^{flx/flx}$ mitochondria; n = 20 CJ in three to four independent tomograms) (Figures 5A and 5B), whereas CJW was not affected by *Mic60* silencing (37.74 ± 1.57 nm in control-silenced mitochondria versus 38.48 ± 1.61 nm in *Mic60*-silenced mitochondria; n = 20 CJ in three to four independent tomograms) (Figures 5A and 5B) or overexpression (37.42 ± 2.09 nm in control-transfected mitochondria versus 37.91 ± 1.81 nm in MIC60-transfected mitochondria; n = 20–26 CJ in three independent tomograms) (Figures 5A and 5C). In conclusion, OPA1 specifies independently of MIC60 not only cristae lumen but also cristae junction width.

MIC60 Requires OPA1 to Stabilize the Inner Membrane-Crista Junction

A closer inspection of tomograms from MIC60-transfected mitochondria revealed a striking increase in the major axis of otherwise normal, narrow CJs (Figure 5A, top right panel). We therefore decided to measure whether levels of MIC60 influenced the CJ-IBM length, i.e., the relative IBM occupancy by an individual CJ (Figure S5). *Mic60* silencing decreased this parameter, whereas its overexpression increased it. While *Opa1* ablation per se did not affect CJ-IBM length, it abolished the increase induced by MIC60 overexpression (Figures 5A, 5D, and 5E); accordingly, OPA1 overexpression blunted the decrease in CJ-IBM length caused by *Mic60* silencing, without influencing per se the degree of IBM occupancy by CJs (Figures 5A, 5F, and 5G). In conclusion, stability of the junction between cristae and IBM requires MIC60; however, OPA1 is epistatic to MIC60 in this function.

MIC60 Is Dispensable for Apoptotic Cristae Remodeling and Cytochrome *c* Mobilization

The extent of CJ-IBM junction might influence cytochrome *c* redistribution, release, and cell death. In such a model, *Mic60* silencing, by reducing the cristae-IBM junction, should inhibit intramitochondrial cytochrome *c* redistribution, release, and

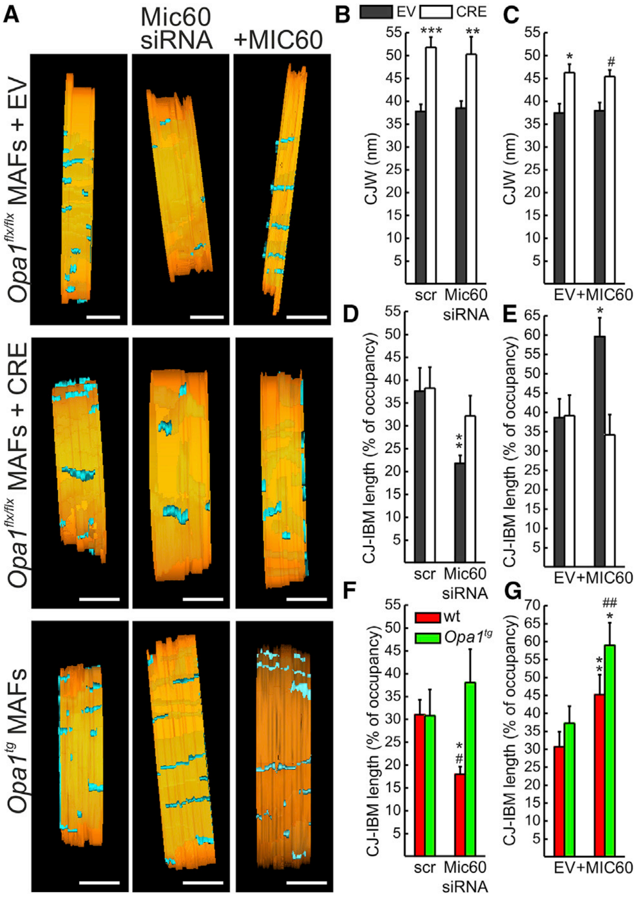

Figure 5. OPA1 Is Epistatic to MIC60 in the Control of CJ Stability and CJW

(A) Surface-rendered views of representative tomographic reconstructions of mitochondria from MAFs of the indicated genotype infected and transfected as indicated. Scr, scramble; +MIC60, MIC60 overexpression; EV, empty vector; Mic60 siRNA, siRNA (3); CRE, Cre recombinase. The inner membrane is pseudocolored in orange, and cristae are pseudocolored in cyan. The outer membrane has been electronically peeled out to highlight the CJ. Scale bars, 120 nm.

(B and C) Quantification of CJW in experiments as in (A). Data represent mean ± SEM of three independent experiments (three to four tomograms per condition). (B) ***p < 0.001 in a paired sample Student's t test between EV-scr and CRE-scr; **p < 0.01 between EV-Mic60 siRNA and CRE-Mic60 siRNA. (C) *p < 0.05 in a paired sample Student's t test between EV-(EV) and CRE-(EV); #p < 0.05 between EV-(+MIC60) and CRE-(+MIC60).

(D–G) Quantification of the percentage of IBM occupied by a CJ in experiments as in (A). Data represent average ± SEM of three independent experiments (three to four tomograms per condition). (D) **p < 0.01 in a paired sample Student's t test between EV-Mic60 siRNA and all other conditions. (E) *p < 0.05 in a paired sample Student's t test between EV-(+MIC60) and all other conditions. (F) *p < 0.05 in one-way ANOVA between wt-Mic60 siRNA and *Opa1*^{tg}-scr or *Opa1*^{tg}-Mic60 siRNA; #p < 0.05 in a paired sample Student's t test between wt-Mic60 siRNA and wt-scr. (G) *p < 0.05 in one-way ANOVA between *Opa1*^{tg}-(+MIC60) and wt-(+MIC60); **p < 0.01 in a paired sample Student's t test between wt-(EV) and wt-(+MIC60); ##p < 0.01 in a paired sample Student's t test between *Opa1*^{tg}-(EV) and *Opa1*^{tg}-(+MIC60). See also Figure S5.

apoptosis. However, quantitation of electron tomograms indicated that *Mic60* silencing did not reduce the CJ widening triggered by the proapoptotic stimulus cBID (Figures 6A and 6B); accordingly, cytochrome *c* mobilization (Figure 6C) and release (Figure 6D) were not affected. Conversely, apoptosis was slightly increased by *Mic60* ablation (Figure 6E), opposite to what the model predicted. This marginal increase in cell death possibly reflects the mitochondrial fragmentation (Figures S6A and S6B) and dysfunction (Figures S6C and S6D) caused by *Mic60* ablation. In conclusion, the MIC60-controlled CJ-IBM length does not participate in the apoptotic redistribution of cytochrome *c* and apoptosis.

DISCUSSION

How membranes are shaped is a crucial question in biology. The case of the inner mitochondrial membrane is particularly interesting because of its organization into two separate yet connected compartments, the IBM and cristae (Frey and Mannella, 2000). The discovery of OPA1 as a master regulator of cristae remodeling was instrumental to probe the role of cristae shape in mitochondrial bioenergetics (Cogliati et al., 2013), apoptosis (Frezza et al., 2006; Yamaguchi et al., 2008), and tissue damage (Varanita et al., 2015). Studies in yeast identified an essential role for MICOS, a large multiprotein complex whose components are partially conserved up to *H. sapiens*, in CJ formation, raising the question of the role of OPA1 in cristae biogenesis and MICOS in cristae remodeling in mammals. Proteomics and biochemistry identify complexes composed of, and physical interactions between, the core MICOS component MIC60 and OPA1. Genetics, electron tomography, and functional studies identify that OPA1 is epistatic to MIC60 in the regulation of CJ number and stability. OPA1 is the sole regulator of CJW and CLW, explaining the marginal effect of MIC60 on cytochrome *c* redistribution, release, and apoptosis.

While in yeast the central MICOS component MIC60 controls not only mitochondrial protein import (Körner et al., 2012; Pfanner et al., 2014; Stojanovski et al., 2012) and inner-outer membrane contact sites (Ott et al., 2012; Xie et al., 2007) but especially cristae and CJ biogenesis (Alkhaja et al., 2012; Darshi et al., 2011; John et al., 2005; Körner et al., 2012), in mammals MIC60 and MICOS complex function is less defined. Mammals lack some MICOS components (Muñoz-Gómez et al., 2015a, 2015b), and our bioinformatics analysis identified some striking primary sequence differences even in the strongly conserved MIC60. At a major difference from yeast, *Chordata* MIC60 harbors a highly conserved TMH with a glycine cluster that probably facilitates interaction with other proteins. This highly conserved TMH appeared in evolution, together with the use of mitochondrial cytochrome *c*, to amplify intrinsic apoptosis, suggesting that mammalian MIC60 could participate in the mitochondrial apoptotic remodeling controlled by OPA1 (Cogliati et al., 2013; Frezza et al., 2006; Jiang et al., 2014; Yamaguchi et al., 2008).

In mammalian mitochondria, MIC60 is retrieved also in ~720 kDa HMWCs (Guarani et al., 2015; Li et al., 2016), the same MW of the OPA1 complexes targeted during cristae remodeling. Proteomic analysis of the ~720 kDa OPA1-containing complexes targeted during cristae remodeling in heart and

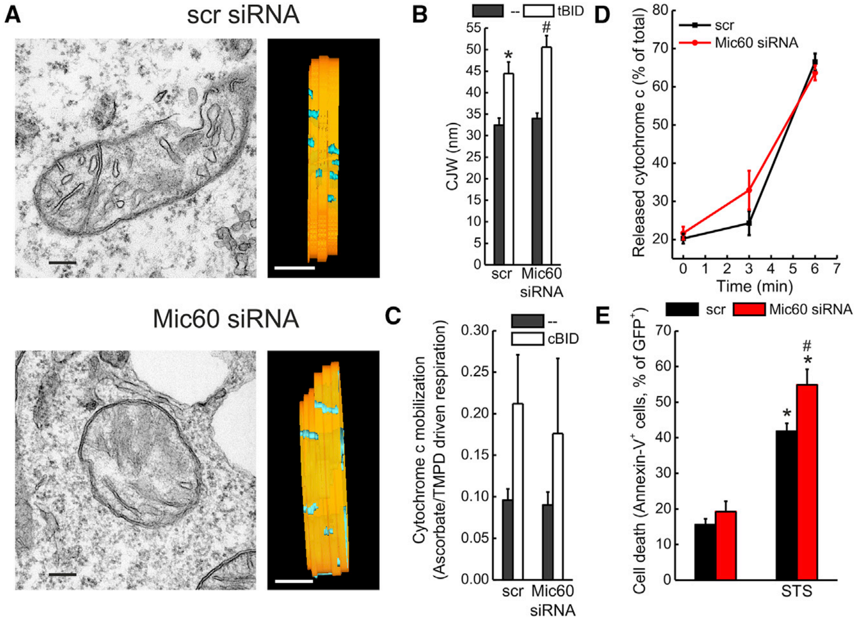

Figure 6. MIC60 Does Not Control Apoptotic Cristae Remodeling and Cytochrome Release

(A) Representative electron micrographs (left panels) and rotations of surface-rendered views of tomographic reconstructions (right panels) of mitochondria from MAFs transfected with scramble (scr) or Mic60 siRNA (2) and transduced with tBID-GFP-expressing retroviruses. In tomographic reconstructions, the inner membrane is depicted in orange and the CJ is depicted in cyan. The outer membrane has been omitted to highlight the CJ. Scale bars, 200 nm (EM) and 120 nm (tomograms).

(B) Quantification of the CJW in experiments as in (A). Data represent average ± SEM of three independent experiments (n = 3 tomograms per condition). *p < 0.05 in a paired sample Student's t test between scr-tBID and scr-untreated (–); #p < 0.05 in a paired sample Student's t test between Mic60 siRNA-tBID and Mic60 siRNA-untreated (–).

(C) Ascorbate/TMPD-driven respiration of mitochondria isolated from MAFs transfected with scramble (scr) or Mic60 siRNA (1–3) and treated where indicated with cBID. Data represent average ± SEM of three independent experiments.

(D) Isolated mitochondria from MAFs transfected as indicated with scr or siRNA Mic60 (1–3) were treated with cBID, and cytochrome *c* release was measured at the indicated time points. Data represent mean ± SEM of four independent experiments.

(E) MAFs transfected with scramble (scr) or Mic60 siRNA (1–3) were treated where indicated with 2 μM staurosporine (STS) for 6 hr, and cell death was determined cytofluorimetrically. Data represent mean ± SEM of three independent experiments. *p < 0.05 in a paired sample Student's t test between scr-STS and scr-untreated and between Mic60 siRNA-STS and Mic60 siRNA-untreated; #p < 0.05 in a paired sample Student's t test between Mic60 siRNA-STS and scr-STS.

fibroblast mitochondria identified MIC60 and the other MICOS component, MIC19. In a proteomic repertoire of mitochondrial proteins co-immunoprecipitated with tagged MICOS components, OPA1 was not cataloged (Guarani et al., 2015). However, MIC60 tagging might have perturbed the MIC60-OPA1 interaction, and several other lines of evidence substantiate the MICOS-OPA1 interaction: (1) in the same complexes targeted during cristae remodeling, we retrieved not only MIC60 and MIC19 but also the MICOS components and interactors QIL1, SAMM50, MTX2, and SLC25A12; (2) OPA1 and MIC60 directly interact; and (3) *Opa1* or *Mic60* deletion destabilizes this HMWC. When *Opa1* is depleted, MIC60 is retrieved not in ~720 kDa complexes but in >1 MDa complexes; when *Mic60* is downregulated, a new ~650 kDa OPA1-containing complex appears, compatible with the loss of MIC60 from the ~720 kDa complex. The partial destabilization of the ~720 kDa OPA1 complex can also lend a molecular explanation to the marginal effect of *Mic60* ablation on CLW, mitochondrial bioenergetics, and cell death, all controlled by this OPA1 complex (Cogliati et al., 2013).

MIC60 and OPA1 not only interact physically but also are components of the same pathway regulating cristae biogenesis. We addressed the roles of MIC60 and OPA1 on a handful of EM or ET cristae morphometric parameters: CJ number, CJW, CJ-IBM occupancy (an indicator of CJ stability), and cristae lumen width (a parameter correlating with RCSs assembly and mitochondrial respiratory efficiency) (Cogliati et al., 2013). This comprehensive morphometry analysis revealed that (1) OPA1 is the sole regulator of CLW and CJW, (2) OPA1 and MIC60 lie in the same pathway controlling CJ number and stability, and (2) surprisingly, OPA1 is epistatic to MIC60. While this work was under revision, Barrera et al., (2016) confirmed that OPA1 and MIC60 interact physically and that levels of the former affect CJ number. However, whether they lie in the same genetic pathway and how they are relatively positioned in the control of the multiple parameters of cristae shape was not addressed (Barrera et al., 2016).

In principle, our genetic analysis does not address whether the epistatic effect of OPA1 is direct or mediated, for example, by the stimulation of ATP synthase dimerization (Patten et al., 2014). Dimers of ATP synthase modulate cristae shape at least in yeast (Paumard et al., 2002), suggesting that a genetic analysis similar to the one presented here should be performed to dissect the relative role of ATPase dimerization subunits in OPA1-MIC60-controlled CJ biogenesis. However, the discovery of a physical interaction between OPA1 and MIC60 strongly suggests that OPA1 directly effects MIC60 to control cristae shape. Our epistatic analysis can also explain molecularly why mild OPA1 overexpression can inhibit apoptotic cristae remodeling and correct models of primary mitochondrial dysfunction with altered cristae shape (Civiletto et al., 2015; Varanita et al., 2015): even if other major cristae biogenesis components like MICOS are altered, OPA1 per se ameliorates all cristae biogenesis parameters. Finally, they can rationalize why *Mic60* silencing reduces CJ number but paradoxically increases cytochrome *c* release and apoptosis (John et al., 2005; Van Laar et al., 2016; Yang et al., 2012): *Mic60* ablation destabilizes the OPA1-containing complex and results in mitochondrial fragmentation and dysfunction, both of which can contribute to cell death.

Our work unravels that OPA1 epistatically influences the core MICOS component MIC60 in CJ biogenesis and assigns OPA1

the key role in the control of apoptotic cristae remodeling. These discoveries pave the way toward investigating how cristae shape coordinates with OPA1-dependent mitochondrial fusion.

EXPERIMENTAL PROCEDURES

Molecular Biology

Details on pcDNA6.2-hMic60-V5 generation, mtRFP plasmids, Mic60 targeting siRNAs, and cBID production can be found in the Supplemental Information.

Cell Culture, Transfection, and Infection

Details on the used cell types can be found in the Supplemental Information. Acute *Opa1* ablation in *Opa1*$^{flx/flx}$ MAFs was obtained by infection with adenoviruses expressing cytomegalovirus (CMV)-Cre-GFP (CRE; 200 pfu/cell; Vector BioLabs) or (CMV)-GFP (EV; Vector BioLabs). Infection efficiency was typically around 80% after 48 hr, as determined by counting GFP-positive cells. Details on simultaneous MIC60 overexpression or silencing and *Opa1* ablation can be found in the Supplemental Information.

Mitochondrial Isolation and In Vitro Assays

Mitochondrial isolation and functional assays were performed as in Frezza et al. (2007). Details can be found in the Supplemental Information.

Biochemistry

Total cell lysates prepared in RIPA buffer were separated under denaturing conditions in Tris-acetate 3%–8% or Bis-Tris 4%–12% (NuPAGE, Life Technologies) polyacrylamide gels, transferred onto polyvinylidene fluoride (PVDF) membranes (Millipore), and probed using the indicated antibodies. Further details on the used antibodies can be found in the Supplemental Information. Details on chemical crosslinking can also be found in the Supplemental Information.

BN-BN-SDS-GE

Digitonin (1.25%, Life Technologies) extracted protein complexes from mitochondria purified from heart (250 μg) or MAFs of the specified genotype after the indicated genetic manipulation (150 μg) were separated by blue native gel electrophoresis (BNGE) on a precast native Bis-Tris 3%–12% gel (Life Technologies).

Two-dimensional (2D) blue native-blue native gel electrophoresis (BN-BNGE) was performed by excising and casting the lane obtained from the first BNGE onto a single-well native gel (NuPAGE Novex Bis-Tris 4%–12% ZOOM gel; Life Technologies), adding 0.02% n-dodecyl-D-maltoside (DDM; Sigma) in the cathode buffers.

Third dimensional (3D) SDS gel electrophoresis (SDS-GE) was performed after excising the diagonal and incubating it with reducing solutions (RSs) RSa for 10 min, RSb for 7 min, and RSc for 12 min to facilitate the complexes' dissociation during the 3D SDS-GE. After the treatment, the diagonal was loaded onto a Bis-Tris 4%–12% ZOOM NuPAGE Novex gel (Life Technologies) and run under denaturating conditions to separate the individual proteins from the complexes, which were then transferred onto PVDF membrane and probed using the indicated antibodies. Further details can be found in the Supplemental Information.

BNGE-Based Semiquantitative Proteomic Analysis, SILAC Labeling, and Quantitative Proteomic Analysis

Details on BNGE-based semiquantitative proteomic analysis can be found in the Supplemental Information. For SILAC labeling, MAFs were grown separately in DMEM containing 4.5 g/L glucose, 2 mM glutamine, 10% fetal bovine serum (FBS), 50 U/mL penicillin, 50 mg/mL streptomycin, 50 mg/mL uridine, and phenol red supplemented with either light L-lysine and L-arginine or heavy [U-13C6]-L-lysine HCl and [U-13C6]-L-arginine (100 mg/L of each amino acid) (SILAC Protein Identification and Quantification Media Kit; Invitrogen). After six doublings, light and heavy cell subpopulations were harvested and mitochondria were isolated separately as described previously. Details on treatments, BNGE, tryptic digestion, liquid chromatography-mass spectrometry (LC-MS), protein identification, and peptide quantification can be found in the Supplemental Information.

Electron Microscopy and Tomography

Electron microscopy and electron tomography were performed as previously described. Details can be found in the Supplemental Information.

Mitochondrial Morphometry

Mitochondrial parameters were measured using ImageJ (NIH) by two operators blinded to the identity of the sample. Cristae lumen width was quantified with the ImageJ Freehand line selection tool. Sample size is indicated in the figure legends.

SUPPLEMENTAL INFORMATION

Supplemental Information includes Supplemental Experimental Procedures and six figures and can be found with this article online at http://dx.doi.org/10.1016/j.celrep.2016.11.049.

AUTHOR CONTRIBUTIONS

Conceptualization, M.E.S. and L.S.; Methodology, E.C., J.V., J.A.E., L.S., and M.E.S.; Software, E.C. and J.V.; Validation, C.G., S.C., and M.E.S.; Formal Analysis, C.G., E.C., J.V., M.L., L.P., and M.E.S.; Investigation, C.G., S.C., A.M., I.A., G.R., E.C., A.R., N.S., and M.E.S.; Resources, J.A.E., J.V., M.E.S., and L.S.; Data Curation, C.G., E.C., L.P., and M.E.S.; Writing – Original Draft, C.G., L.P., L.S., and M.E.S.; Writing – Review & Editing, C.G., M.E.S., and L.S.; Visualization, C.G., E.C., L.P., L.S., and M.E.S., Supervision, M.E.S.; Project Administration, M.E.S.; Funding Acquisition, L.S. and M.E.S.

ACKNOWLEDGMENTS

We thank Drs. F. Caicci and F. Boldrin (EM Facility, Department of Biology, University of Padova) for EM and ALEMBIC, San Raffaele Scientific Institute, for tomography. L.S. is a senior scientist of the Dulbecco-Telethon Institute. Support was provided by Telethon-Italy (GGP15091 and GGP14187), AIRC Italy (ERC FP7-282280), FP7 CIG (PCIG13-GA-2013-618697), the Italian Ministry of Research (FIRB RBAP11Z3YA_005), the Italian Ministry of Health (GR-2009-1600051 to L.S.), a University of Padua grant for a postdoctoral fellowship (2015 to M.E.S.), and an International Brain Research Organization-International Society for Neurochemistry research fellowship (2016 to A.M.).

Received: July 19, 2016
Revised: August 31, 2016
Accepted: November 10, 2016
Published: December 13, 2016

REFERENCES

Acin-Perez, R., and Enriquez, J.A. (2014). The function of the respiratory supercomplexes: the plasticity model. Biochim. Biophys. Acta *1837*, 444–450.

Acín-Pérez, R., Fernández-Silva, P., Peleato, M.L., Pérez-Martos, A., and Enriquez, J.A. (2008). Respiratory active mitochondrial supercomplexes. Mol. Cell *32*, 529–539.

Alkhaja, A.K., Jans, D.C., Nikolov, M., Vukotic, M., Lytovchenko, O., Ludewig, F., Schliebs, W., Riedel, D., Urlaub, H., Jakobs, S., and Deckers, M. (2012). MINOS1 is a conserved component of mitofilin complexes and required for mitochondrial function and cristae organization. Mol. Biol. Cell *23*, 247–257.

Barbot, M., Jans, D.C., Schulz, C., Denkert, N., Kroppen, B., Hoppert, M., Jakobs, S., and Meinecke, M. (2015). Mic10 oligomerizes to bend mitochondrial inner membranes at cristae junctions. Cell Metab. *21*, 756–763.

Barrera, M., Koob, S., Dikov, D., Vogel, F., and Reichert, A.S. (2016). OPA1 functionally interacts with MIC60 but is dispensable for crista junction formation. FEBS Lett. *590*, 3309–3322.

Bohnert, M., Zerbes, R.M., Davies, K.M., Mühleip, A.W., Rampelt, H., Horvath, S.E., Boenke, T., Kram, A., Perschil, I., Veenhuis, M., et al. (2015). Central role of Mic10 in the mitochondrial contact site and cristae organizing system. Cell Metab. *21*, 747–755.

Cipolat, S., Martins de Brito, O., Dal Zilio, B., and Scorrano, L. (2004). OPA1 requires mitofusin 1 to promote mitochondrial fusion. Proc. Natl. Acad. Sci. USA *101*, 15927–15932.

Civiletto, G., Varanita, T., Cerutti, R., Gorletta, T., Barbaro, S., Marchet, S., Lamperti, C., Viscomi, C., Scorrano, L., and Zeviani, M. (2015). Opa1 overexpression ameliorates the phenotype of two mitochondrial disease mouse models. Cell Metab. *21*, 845–854.

Cogliati, S., Frezza, C., Soriano, M.E., Varanita, T., Quintana-Cabrera, R., Corrado, M., Cipolat, S., Costa, V., Casarin, A., Gomes, L.C., et al. (2013). Mitochondrial cristae shape determines respiratory chain supercomplexes assembly and respiratory efficiency. Cell *155*, 160–171.

Cogliati, S., Enriquez, J.A., and Scorrano, L. (2016). Mitochondrial cristae: where beauty meets functionality. Trends Biochem. Sci. *41*, 261–273.

Corrado, M., Mariotti, F.R., Trapani, L., Taraborrelli, L., Nazio, F., Cianfanelli, V., Soriano, M.E., Schrepfer, E., Cecconi, F., Scorrano, L., and Campello, S. (2016). Macroautophagy inhibition maintains fragmented mitochondria to foster T cell receptor-dependent apoptosis. EMBO J. *35*, 1793–1809.

Costa, V., Giacomello, M., Hudec, R., Lopreiato, R., Ermak, G., Lim, D., Malorni, W., Davies, K.J., Carafoli, E., and Scorrano, L. (2010). Mitochondrial fission and cristae disruption increase the response of cell models of Huntington's disease to apoptotic stimuli. EMBO Mol. Med. *2*, 490–503.

Darshi, M., Mendiola, V.L., Mackey, M.R., Murphy, A.N., Koller, A., Perkins, G.A., Ellisman, M.H., and Taylor, S.S. (2011). ChChd3, an inner mitochondrial membrane protein, is essential for maintaining crista integrity and mitochondrial function. J. Biol. Chem. *286*, 2918–2932.

Frey, T.G., and Mannella, C.A. (2000). The internal structure of mitochondria. Trends Biochem. Sci. *25*, 319–324.

Frezza, C., Cipolat, S., Martins de Brito, O., Micaroni, M., Beznoussenko, G.V., Rudka, T., Bartoli, D., Polishuck, R.S., Danial, N.N., De Strooper, B., and Scorrano, L. (2006). OPA1 controls apoptotic cristae remodeling independently from mitochondrial fusion. Cell *126*, 177–189.

Frezza, C., Cipolat, S., and Scorrano, L. (2007). Organelle isolation: functional mitochondria from mouse liver, muscle and cultured fibroblasts. Nat. Protoc. *2*, 287–295.

Friedman, J.R., Mourier, A., Yamada, J., McCaffery, J.M., and Nunnari, J. (2015). MICOS coordinates with respiratory complexes and lipids to establish mitochondrial inner membrane architecture. eLife *4*. http://dx.doi.org/10.7554/eLife.07739.

Gilkerson, R.W., Selker, J.M., and Capaldi, R.A. (2003). The cristal membrane of mitochondria is the principal site of oxidative phosphorylation. FEBS Lett. *546*, 355–358.

Große, L., Wurm, C.A., Brüser, C., Neumann, D., Jans, D.C., and Jakobs, S. (2016). Bax assembles into large ring-like structures remodeling the mitochondrial outer membrane in apoptosis. EMBO J. *35*, 402–413.

Guarani, V., McNeill, E.M., Paulo, J.A., Huttlin, E.L., Fröhlich, F., Gygi, S.P., Van Vactor, D., and Harper, J.W. (2015). QIL1 is a novel mitochondrial protein required for MICOS complex stability and cristae morphology. eLife *4*. http://dx.doi.org/10.7554/eLife.06265.

Hackenbrock, C.R. (1966). Ultrastructural bases for metabolically linked mechanical activity in mitochondria. I. Reversible ultrastructural changes with change in metabolic steady state in isolated liver mitochondria. J. Cell Biol. *30*, 269–297.

Herrmann, J.M. (2011). MINOS is plus: a Mitofilin complex for mitochondrial membrane contacts. Dev. Cell *21*, 599–600.

Huynen, M.A., Mühlmeister, M., Gotthardt, K., Guerrero-Castillo, S., and Brandt, U. (2016). Evolution and structural organization of the mitochondrial contact site (MICOS) complex and the mitochondrial intermembrane space bridging (MIB) complex. Biochim. Biophys. Acta *1863*, 91–101.

Jans, D.C., Wurm, C.A., Riedel, D., Wenzel, D., Stagge, F., Deckers, M., Rehling, P., and Jakobs, S. (2013). STED super-resolution microscopy reveals an array of MINOS clusters along human mitochondria. Proc. Natl. Acad. Sci. USA *110*, 8936–8941.

Jiang, X., Jiang, H., Shen, Z., and Wang, X. (2014). Activation of mitochondrial protease OMA1 by Bax and Bak promotes cytochrome *c* release during apoptosis. Proc. Natl. Acad. Sci. USA *111*, 14782–14787.

John, G.B., Shang, Y., Li, L., Renken, C., Mannella, C.A., Selker, J.M., Rangell, L., Bennett, M.J., and Zha, J. (2005). The mitochondrial inner membrane protein mitofilin controls cristae morphology. Mol. Biol. Cell *16*, 1543–1554.

Körner, C., Barrera, M., Dukanovic, J., Eydt, K., Harner, M., Rabl, R., Vogel, F., Rapaport, D., Neupert, W., and Reichert, A.S. (2012). The C-terminal domain of Fcj1 is required for formation of crista junctions and interacts with the TOB/SAM complex in mitochondria. Mol. Biol. Cell *23*, 2143–2155.

Landes, T., Emorine, L.J., Courilleau, D., Rojo, M., Belenguer, P., and Arnauné-Pelloquin, L. (2010). The BH3-only Bnip3 binds to the dynamin Opa1 to promote mitochondrial fragmentation and apoptosis by distinct mechanisms. EMBO Rep. *11*, 459–465.

Lapuente-Brun, E., Moreno-Loshuertos, R., Acín-Pérez, R., Latorre-Pellicer, A., Colás, C., Balsa, E., Perales-Clemente, E., Quirós, P.M., Calvo, E., Rodríguez-Hernández, M.A., et al. (2013). Supercomplex assembly determines electron flux in the mitochondrial electron transport chain. Science *340*, 1567–1570.

Li, H., Ruan, Y., Zhang, K., Jian, F., Hu, C., Miao, L., Gong, L., Sun, L., Zhang, X., Chen, S., et al. (2016). Mic60/Mitofilin determines MICOS assembly essential for mitochondrial dynamics and mtDNA nucleoid organization. Cell Death Differ. *23*, 380–392.

Muñoz-Gómez, S.A., Slamovits, C.H., Dacks, J.B., Baier, K.A., Spencer, K.D., and Wideman, J.G. (2015a). Ancient homology of the mitochondrial contact site and cristae organizing system points to an endosymbiotic origin of mitochondrial cristae. Curr. Biol. *25*, 1489–1495.

Muñoz-Gómez, S.A., Slamovits, C.H., Dacks, J.B., and Wideman, J.G. (2015b). The evolution of MICOS: Ancestral and derived functions and interactions. Commun. Integr. Biol. *8*, e1094593.

Ott, C., Ross, K., Straub, S., Thiede, B., Götz, M., Goosmann, C., Krischke, M., Mueller, M.J., Krohne, G., Rudel, T., and Kozjak-Pavlovic, V. (2012). Sam50 functions in mitochondrial intermembrane space bridging and biogenesis of respiratory complexes. Mol. Cell. Biol. *32*, 1173–1188.

Patten, D.A., Wong, J., Khacho, M., Soubannier, V., Mailloux, R.J., Pilon-Larose, K., MacLaurin, J.G., Park, D.S., McBride, H.M., Trinkle-Mulcahy, L., et al. (2014). OPA1-dependent cristae modulation is essential for cellular adaptation to metabolic demand. EMBO J. *33*, 2676–2691.

Paumard, P., Vaillier, J., Coulary, B., Schaeffer, J., Soubannier, V., Mueller, D.M., Brèthes, D., di Rago, J.P., and Velours, J. (2002). The ATP synthase is involved in generating mitochondrial cristae morphology. EMBO J. *21*, 221–230.

Pernas, L., and Scorrano, L. (2016). Mito-morphosis: mitochondrial fusion, fission, and cristae remodeling as key mediators of cellular function. Annu. Rev. Physiol. *78*, 505–531.

Pfanner, N., van der Laan, M., Amati, P., Capaldi, R.A., Caudy, A.A., Chacinska, A., Darshi, M., Deckers, M., Hoppins, S., Icho, T., et al. (2014). Uniform nomenclature for the mitochondrial contact site and cristae organizing system. J. Cell Biol. *204*, 1083–1086.

Rabl, R., Soubannier, V., Scholz, R., Vogel, F., Mendl, N., Vasiljev-Neumeyer, A., Körner, C., Jagasia, R., Keil, T., Baumeister, W., et al. (2009). Formation of cristae and crista junctions in mitochondria depends on antagonism between Fcj1 and Su e/g. J. Cell Biol. *185*, 1047–1063.

Scorrano, L., Ashiya, M., Buttle, K., Weiler, S., Oakes, S.A., Mannella, C.A., and Korsmeyer, S.J. (2002). A distinct pathway remodels mitochondrial cristae and mobilizes cytochrome *c* during apoptosis. Dev. Cell *2*, 55–67.

Stojanovski, D., Bragoszewski, P., and Chacinska, A. (2012). The MIA pathway: a tight bond between protein transport and oxidative folding in mitochondria. Biochim. Biophys. Acta *1823*, 1142–1150.

van der Laan, M., Horvath, S.E., and Pfanner, N. (2016). Mitochondrial contact site and cristae organizing system. Curr. Opin. Cell Biol. *41*, 33–42.

Van Laar, V.S., Berman, S.B., and Hastings, T.G. (2016). Mic60/mitofilin overexpression alters mitochondrial dynamics and attenuates vulnerability of dopaminergic cells to dopamine and rotenone. Neurobiol. Dis. *91*, 247–261.

Varanita, T., Soriano, M.E., Romanello, V., Zaglia, T., Quintana-Cabrera, R., Semenzato, M., Menabò, R., Costa, V., Civiletto, G., Pesce, P., et al. (2015). The OPA1-dependent mitochondrial cristae remodeling pathway controls atrophic, apoptotic, and ischemic tissue damage. Cell Metab. *21*, 834–844.

Xie, J., Marusich, M.F., Souda, P., Whitelegge, J., and Capaldi, R.A. (2007). The mitochondrial inner membrane protein mitofilin exists as a complex with SAM50, metaxins 1 and 2, coiled-coil-helix coiled-coil-helix domain-containing protein 3 and 6 and DnaJC11. FEBS Lett. *581*, 3545–3549.

Yamaguchi, R., Lartigue, L., Perkins, G., Scott, R.T., Dixit, A., Kushnareva, Y., Kuwana, T., Ellisman, M.H., and Newmeyer, D.D. (2008). Opa1-mediated cristae opening is Bax/Bak and BH3 dependent, required for apoptosis, and independent of Bak oligomerization. Mol. Cell *31*, 557–569.

Yang, R.F., Zhao, G.W., Liang, S.T., Zhang, Y., Sun, L.H., Chen, H.Z., and Liu, D.P. (2012). Mitofilin regulates cytochrome *c* release during apoptosis by controlling mitochondrial cristae remodeling. Biochem. Biophys. Res. Commun. *428*, 93–98.

Zerbes, R.M., van der Klei, I.J., Veenhuis, M., Pfanner, N., van der Laan, M., and Bohnert, M. (2012). Mitofilin complexes: conserved organizers of mitochondrial membrane architecture. Biol. Chem. *393*, 1247–1261.

Cell Reports, Volume *17*

Supplemental Information

Optic Atrophy 1 Is Epistatic to the Core MICOS Component MIC60 in Mitochondrial Cristae Shape Control

Christina Glytsou, Enrique Calvo, Sara Cogliati, Arpit Mehrotra, Irene Anastasia, Giovanni Rigoni, Andrea Raimondi, Norihito Shintani, Marta Loureiro, Jesùs Vazquez, Luca Pellegrini, Jose Antonio Enriquez, Luca Scorrano, and Maria Eugenia Soriano

Supplemental Figures

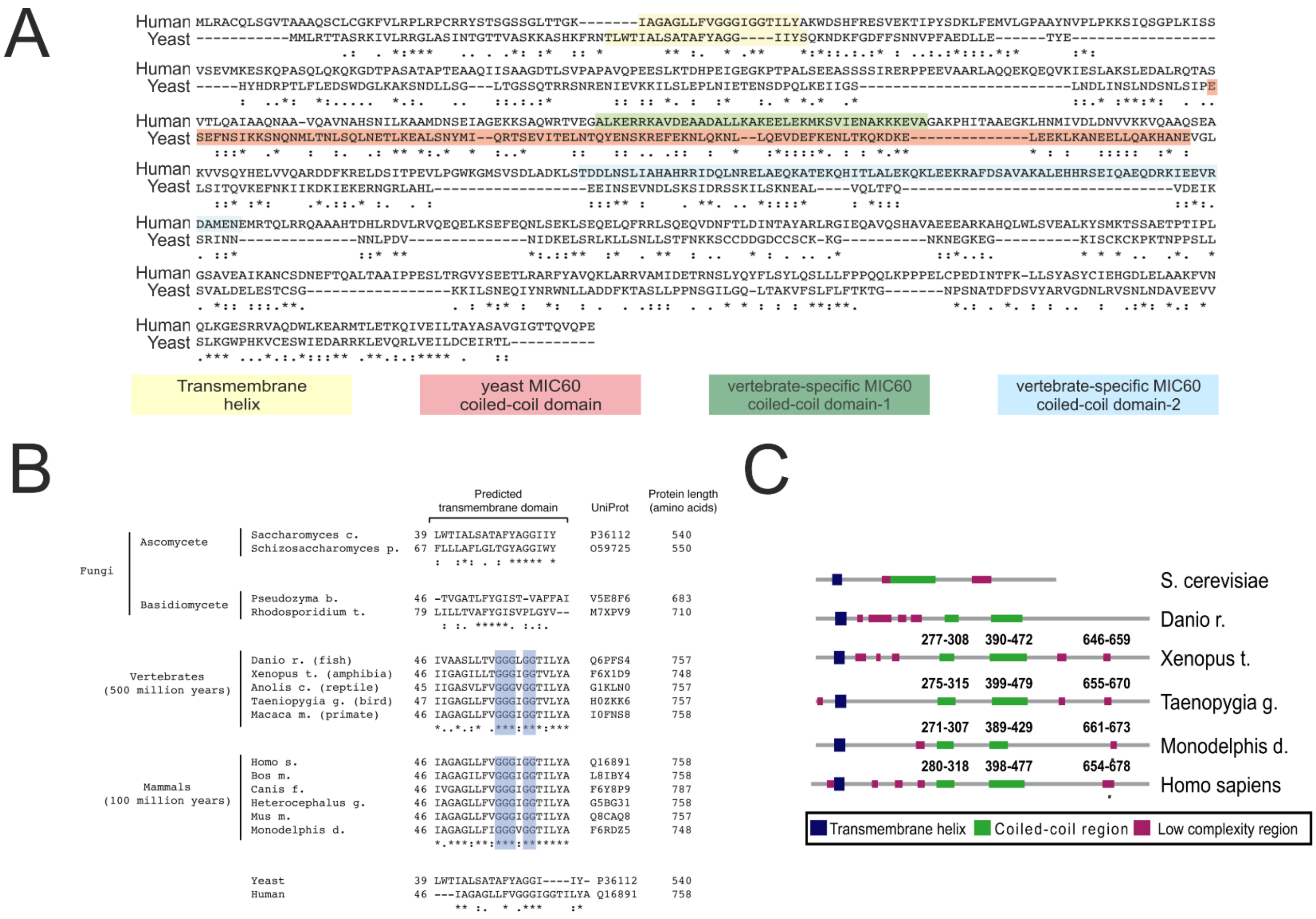

Figure S1. Comparative evolutionary analysis of MIC60 homologues in fungi and vertebrates. Related to Figure 1.
(A) Bioinformatic analysis showing limited evolutionary and structural conservation between yeast and mammalian MIC60 homologs. The predicted transmembrane and coil-coiled domains are highlighted.
(B) Multiple amino acid sequence alignment of the predicted transmembrane domain of MIC60 homologues identified in the genomes of key eukaryotic model organisms (fungi, vertebrates, and mammals). The boxed areas indicate the clusters of glycine residues.
(C) Spatial and structural organization of the predicted protein domains that compose MIC60 homologues showing conservation among vertebrates but not between mammals and yeast.

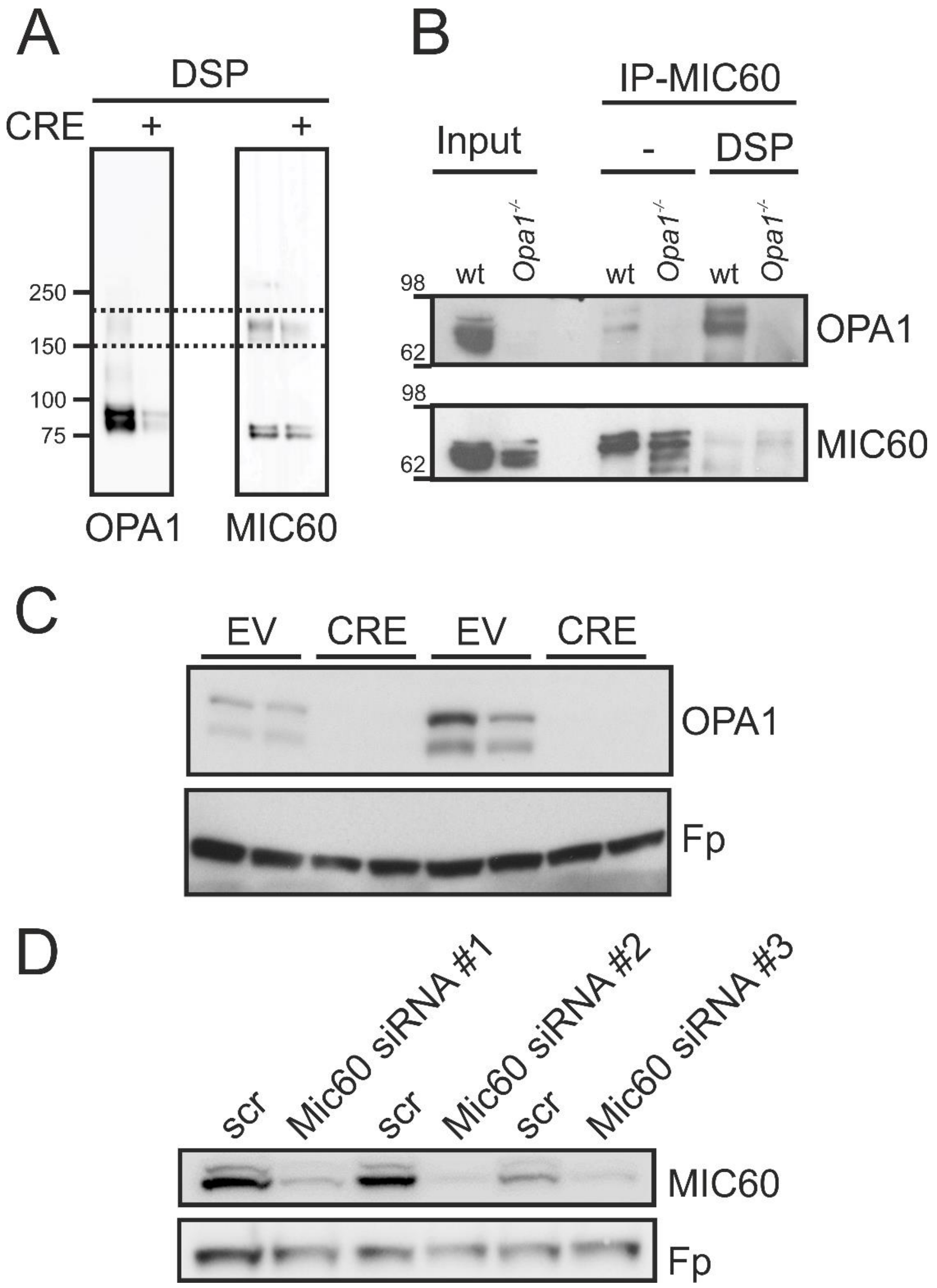

Figure S2. OPA1-MIC60 crosslinked complexes co-immunoprecipitate. Related to Figure 2.

(A) Isolated mitochondria from *Opa1*$^{flx/flx}$ MAFs infected with the indicated adenoviruses (EV, empty vector-GFP; CRE, Cre recombinase-GFP) were crosslinked with 2 mM DSP and equal amounts (20 µg) of proteins were separated in Tris-Acetate 3%-8% gel and immunoblotted with the indicated antibodies. The boxed are shows OPA1 and MIC60 complexes with the same electrophoretic migration.

(B) Lysates from DSP-crosslinked or native mitochondria (125 µg) were immunoprecipitated with anti-MIC60 antibody coupled to Protein-A agarose beads and bound proteins were separated by SDS-GE and immunoblotted using the indicated antibodies. Input is diluted 1:10.

(C) Western blot analysis of OPA1 protein levels after acute ablation. Equal amounts of proteins (40 µg) from *Opa1*$^{flx/flx}$ MAFs infected with the indicated adenoviruses (EV, empty vector-GFP; CRE, Cre recombinase-GFP) were separated by SDS-GE and immunoblotted with the indicated antibodies.

(D) Western blot analysis of MIC60 protein levels after silencing. Equal amounts of proteins (40 µg) from MAFs transfected with scramble (scr) or three independent Mic60 siRNAs (#1, #2 or #3) were separated by SDS-GE and immunoblotted with the indicated antibodies. All three Mic60 siRNA oligos were used independently to perform all the experiments where *Mic60* silencing is indicated. For each figure the specific Mic60 siRNA used is indicated in the figure legend.

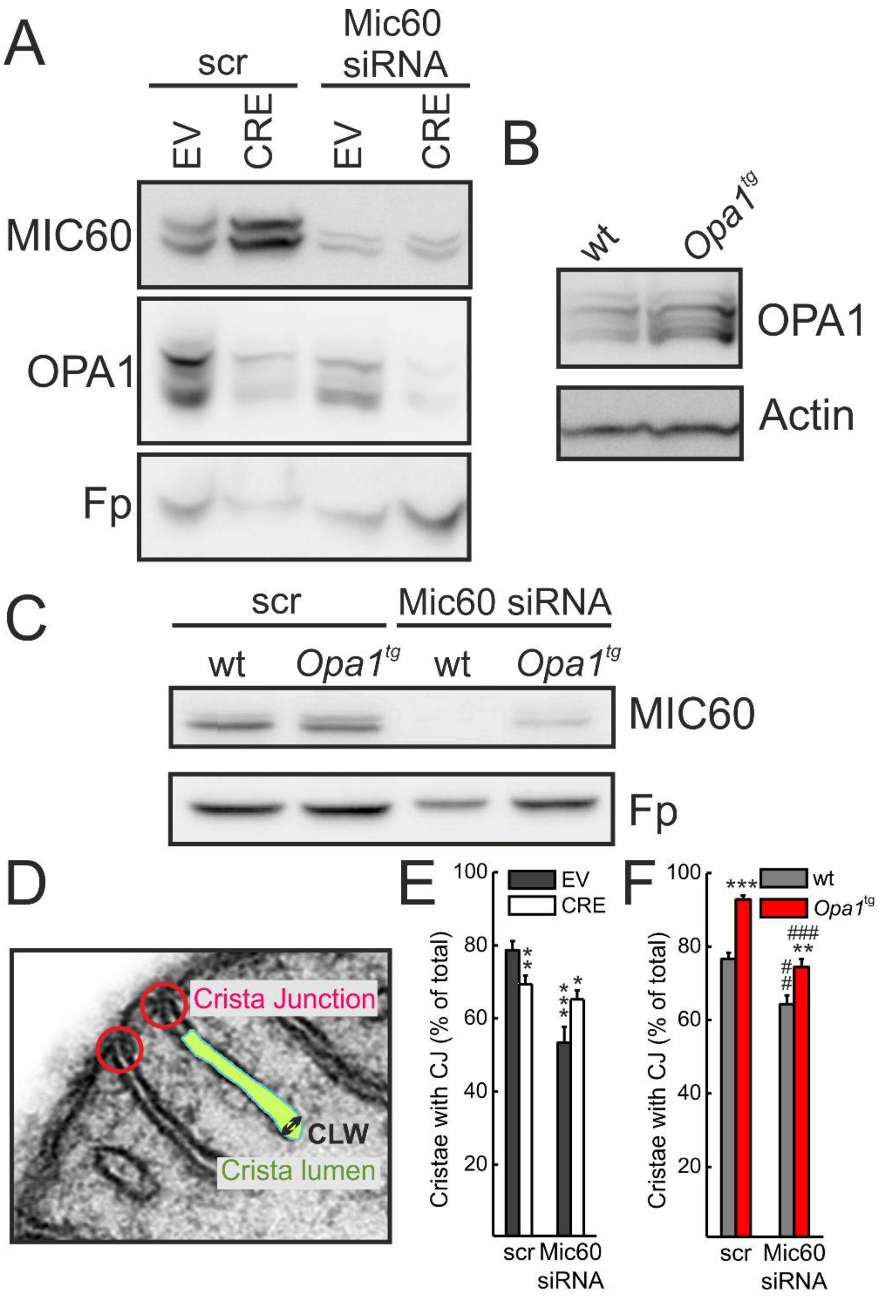

Figure S3. MIC60 and OPA1 are in the same pathway controlling CJ biogenesis. Related to Figure 3.

(A) Protein levels of MIC60 and OPA1 after *Mic60* knock-down (Mic60 siRNA #2) and/or *Opa1* acute ablation *Opa1*$^{flx/flx}$ MAFs. Equal amounts of protein (25 µg) were loaded onto a Tris-Acetate 3%-8% gel and immunoblotted with the indicated antibodies. EV: empty vector-GFP; CRE: Cre recombinase-GFP; scr: scramble; Mic60 siRNA: MIC60 silencing.

(B) OPA1 protein levels in wt and *Opa1*^{tg} MAFs. Equal amounts of protein samples (25 µg) were separated in a Tris-Acetate 3%-8% gel and immunoblotted with the indicated antibodies.

(C) MIC60 protein levels after *Mic60* knock-down (Mic60 siRNA #2) in wt and *Opa1*^{tg} MAFs. Equal amounts of protein samples (25 µg) were separated in a Tris-Acetate 3%-8% gel and immunoblotted with the indicated antibodies.

(D) Scheme illustrating the CJ (red circles) and CLW (black arrow) parameters. Cristae lumen is colored in green.

(E) Percentage of cristae with CJ in 100-150 randomly selected mitochondria from each experimental condition, as indicated. Data represent average ± SEM of 4 independent experiments. ***p<0.001 in a paired sample Student's t-test between EV-scr and -Mic60 siRNA; **p < 0.01 in a paired sample Student's t-test between EV- and CRE-scr; *p<0.05 in a paired sample Student's t-test between EV- and CRE-Mic60 siRNA.

(F) Percentage of cristae with CJ in 100-150 randomly selected mitochondria from each experimental condition, as indicated. Data represent average ± SEM of 4 independent experiments. ***p<0.001 in one-way ANOVA between wt- and *Opa1*^{tg}-scr; ## p<0.01 in a paired sample Student's t-test between wt-scr and -Mic60 siRNA; ### p<0.001 in a paired sample Student's t-test between *Opa1*^{tg}-scr and -Mic60 siRNA*;* **p<0.01 in one-way ANOVA between wt- and *Opa1*^{tg}-Mic60 siRNA.

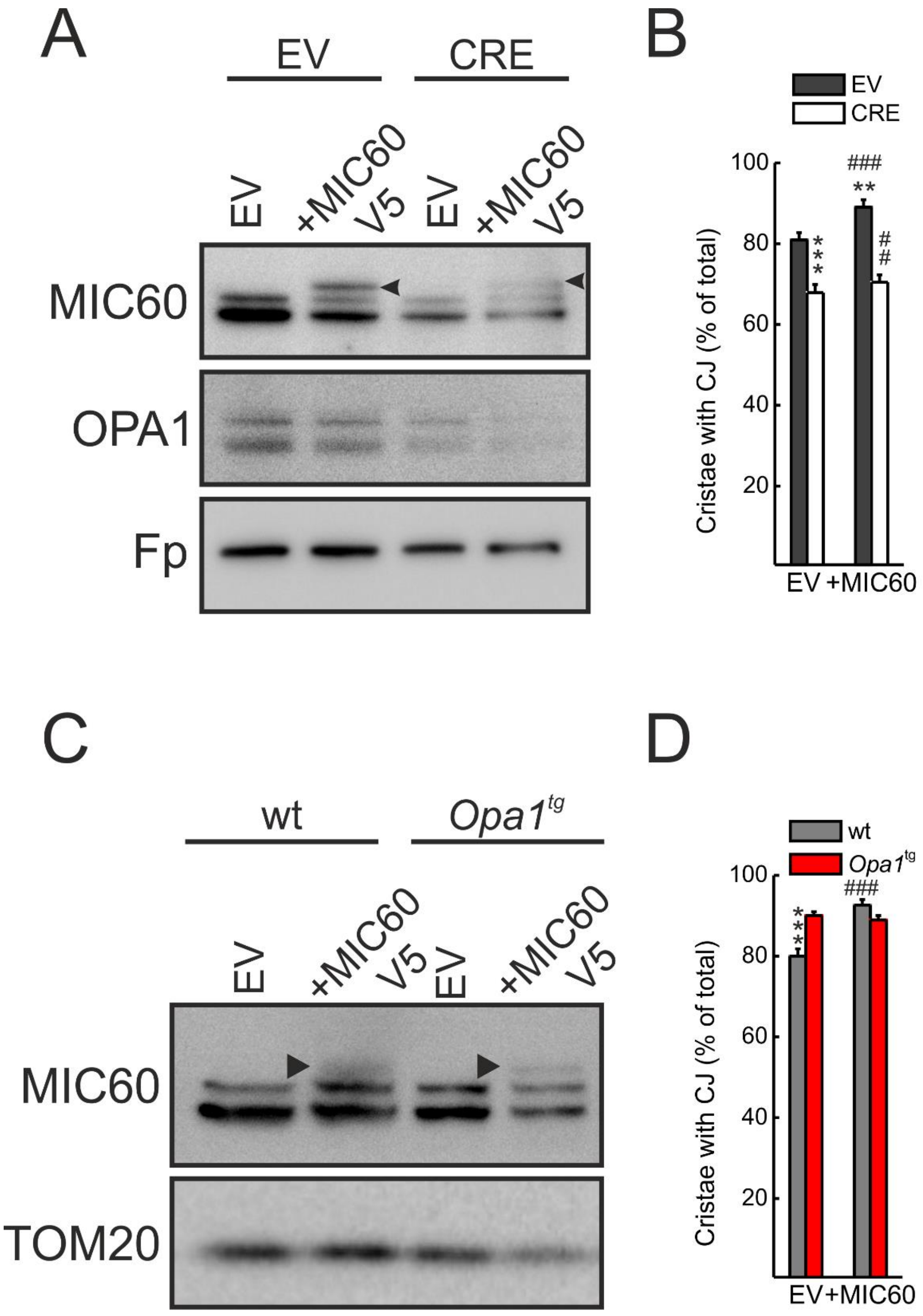

Figure S4. OPA1 is epistatic to MIC60 in regulation of CJ biogenesis. Related to Figure 4.

(A) Protein levels of MIC60 and OPA1 after MIC60 overexpression and/or *Opa1* acute ablation in *Opa1*$^{flx/flx}$ MAFs. Equal amounts of protein lysates (25 µg) were loaded onto a 3%-8% Tris-Acetate gel and immunoblotted with the indicated antibodies. EV: empty vector-GFP; CRE: Cre recombinase-GFP; +MIC60-V5: overexpression of MIC60.

(B) Percentage of cristae with CJ in 100-150 randomly selected mitochondria from each experimental condition. Data represent average ± SEM of four independent experiments. ***$p<0.001$ in a paired sample Student's t-test between EV- and CRE-(EV); ### $p<0.001$ in a paired sample Student's t-test between EV- and CRE-(+MIC60); **$p<0.01$ in a paired sample Student's t-test between EV-(EV) and -(+MIC60); ## $p<0.01$ in a paired sample Student's t-test between EV-(EV) and CRE-(+MIC60).

(C) Protein levels of MIC60 after MIC60 overexpression in wt and *Opa1*^{tg} MAFs. Equal amounts of protein samples (25 µg) were separated in a Tris-Acetate 3%-8% gel and immunoblotted with the indicated antibodies.

(D) Percentage of cristae with CJ in 100-150 randomly selected mitochondria from each experimental condition. Data represent average ± SEM of four independent experiments. ***$p<0.001$ in one-way ANOVA between wt- and *Opa1*^{tg}-(EV) or *Opa1*^{tg}-(+MIC60); ### $p<0.001$ in a paired sample Student's t-test between wt-(EV) and -(+MIC60).

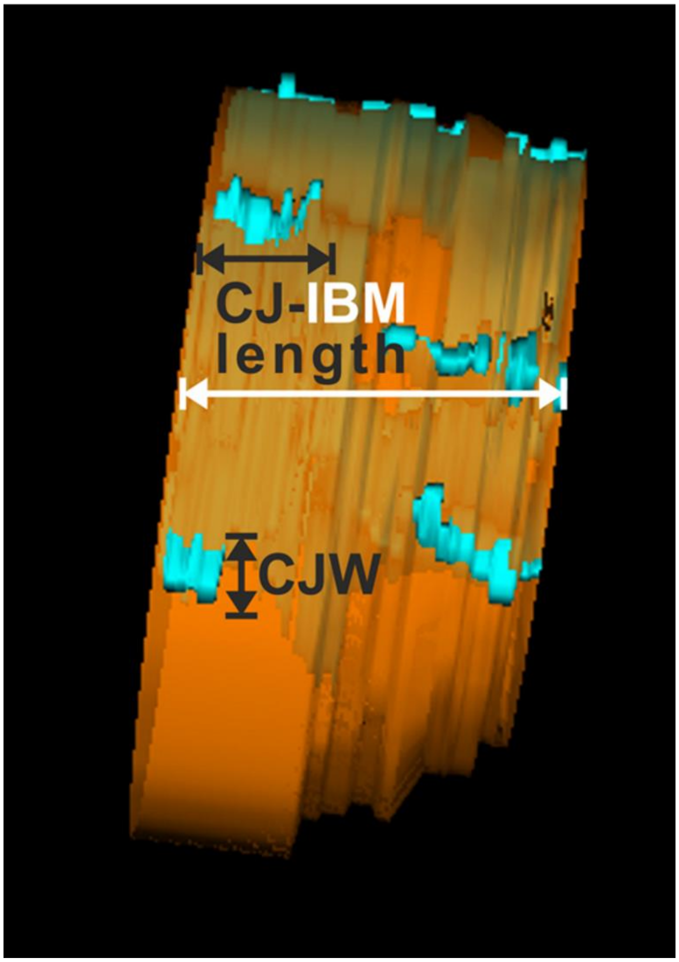

Figure S5. CJ parameters measurements on a 3D tomographic reconstruction of a mitochondrion. Related to Figure 5.
Inner membrane is depicted in orange and cristae junction in cyan. Outer membrane has been peeled out to highlight CJ. The length of the inner boundary membrane (IBM) is denoted with a white double-edged arrow. Cristae junction width (CJW) and cristae junction-inner boundary membrane length (CJ-IBM length) are denoted with black double-edged arrows. The CJW corresponds to the length of the vertical line that defines the CJ in the different frames. CJ-IBM length corresponds to the length of the horizontal line that defines the presence of the same CJ over successive tomography frames. Details in their quantification are described in *Experimental procedures section.*

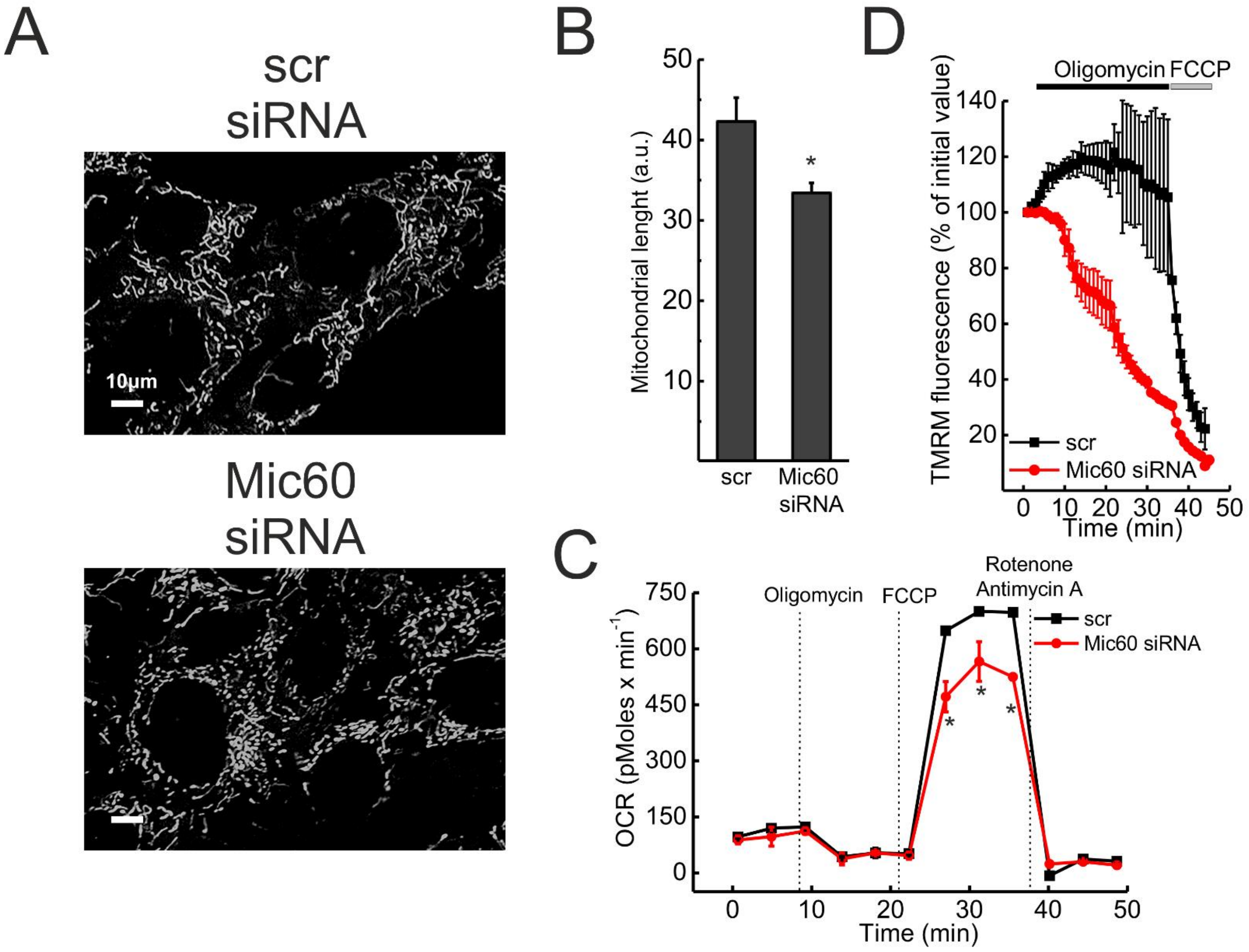

Figure S6. ***Mic60* silencing leads to mitochondrial dysfunction. Related to Figure 6.**

(A) Representative confocal images of MAFs transfected with scramble (scr) or Mic60 siRNA (#3) and labelled with tetramethylrhodamine methyl ester (TMRM). Scale bar: 10 µm.

(B) Average mitochondrial major axis length. Experiments were as in (A). Data represent average ± SEM of three independent experiments (six mitochondria per cell, at least 30 cells/experiment). *p<0.05 in a paired sample Student's t-test.

(C) Seahorse analysis of MAFs upon *Mic60* silencing (Mic60 siRNA #1-3). Oxygen consumption rate was measured in basal conditions and after the addition of oligomycin 1.3 µM, FCCP 0.4 µM and Rotenone 1 µM/Antimycin A 1.2 µM. Data represent average ± SEM of three independent experiments. * p<0.05 in a paired sample Student's t-test.

(D) Mitochondrial membrane potential in MAFs transfected with scramble (scr) or Mic60 siRNA (Mic60 siRNA #1-3) was monitored following TMRM fluorescence change. Where indicated, 2 µM oligomycin and 2 µM FCCP were added. Data represent average ± SEM of four independent experiments.

Supplemental Experimental Procedures. Related to Experimental Procedures

Bioinformatic analysis

The non-redundant protein sequence database at the National Center for Biotechnology Information (NIH, Bethesda) was searched iteratively using the PSI-BLAST program with the yeast and human MIC60 as starting queries. PSI-BLAST was run with expectation value of 0.01 as the cut-off for inclusion of sequences into the position-specific scoring matrix. Multiple alignments of MIC60 homologues were constructed using the MUltiple Sequence Comparison by Log- Expectation program (MUSCLE) and manually adjusted on the basis of the examination of PSI-BLAST search outputs and the superposition of the predicted transmembrane helices. Transmembrane helices were manually predicted and validated using the TMpred, HMMTOP, and TMHMM programs. Protein domains composition and location were detected and analyzed using the Simple Modular Architecture Research Tool program (SMART).

Molecular Biology

pcDNA6.2-hMic60-V5 vector was generated by subcloning the human, transcript variant 1 DNA NM_006839.1 from donor plasmid pCMV6-AC to pDEST-V5, using the Gateway technology (ThermoFisher). Mitochondrially targeted red fluorescent protein (mtRFP), corresponding to pDsRed2-Mito was previously described (Cipolat et al., 2004).
For Mic60 silencing the following siRNAs were used: Stealth Mic60 siRNA#1 (MSS233674, LifeTechnologies), Mic60 siRNA#2 (MSS233683, LifeTechnologies) or Mic60 siRNA#3 (MSS233684, LifeTechnologies). Silencer Negative Control N° 1 siRNA (Ambion, Life technologies).
Recombinant wild type and mutant BID were produced, purified and cleaved with caspase-8, as previously described (Frezza et al., 2006).

Cell culture, Transfection and Infection

Mouse embryonic fibroblasts (MEFs) and wild type (WT), *Opa1*$^{flx/flx}$ and *Opa1*^{tg} SV40 transformed mouse adult fibroblast (MAFs) were cultured as previously described (Cogliati et al., 2013).
For MIC60 overexpression and simultaneous acute *Opa1* ablation, *Opa1*$^{flx/flx}$ MAFs were infected with the indicated adenoviruses, and after 16 hrs co-transfected with mtRFP and pcDNA6.2-hMIC60-V5 (+MIC60) or pcDNA6.2 (EV) using Lipofectamine. After 24 hrs GFP-positive and RFP–positive cells were sorted and subcultured for 16 hrs before being processed for further analysis.
For *Mic60* silencing (Mic60 siRNA) and simultaneous acute *Opa1* ablation, *Opa1*$^{flx/flx}$ MAFs were infected with the indicated adenoviruses, and after 24 hrs cells were transfected with the indicated siRNA. Cells were sorted for GFP-positive cells and processed for further analysis.

Mitochondrial isolation and *in vitro* assays

Mitochondria from mouse adult fibroblasts (MAFs) and mouse liver and heart tissues were isolated as previously described (Frezza et al., 2007).
Mitochondria (1 mg/ml) were resuspended in Experimental Buffer (EB) (Frezza et al., 2007) and treated as detailed in the Figure legends. Cytochrome c redistribution and release assays were performed as previously described (Frezza et al., 2006).

Electrophoresis

For 1st dimension BNGE sample preparation, isolated mitochondria treated as specified in the Figure legends were resuspended in Native solubilization buffer (NB, 50µl) containing Native Sample buffer (Life Technologies), 1.25% digitonin (Life Technologies) and 1:100 protease inhibitor cocktail (PIC, SIGMA). After 10 min incubation on ice, samples were centrifuged at 25000 x *g* at 4°C. Supernatants were collected and 2 µl of 5% Coomassie G-250 (Life Technologies) were added before loading. Electrophoresis was performed in the presence of cathode (dark blue cathode: 45 min; light blue cathode: 90 min) and anode buffers prepared following manufacturer's instructions (Life Technologies) in precast native Bis-Tris 3%-12% gel (Life Technologies). When indicated, protein complexes were blotted onto polyvinylidene difluoride (PVDF) membranes (Millipore) and probed for the indicated antibodies or stained with Coomassie Blue (40% methanol, 10% acetic acid, 0.25 gr Coomassie Brilliant Blue R-250 - Bio-Rad).
2nd dimension BN-BNGE was performed at 125 V for 1.5 hr using dark cathode buffer and at 150 V, overnight with light cathode buffer. During 2D BN-BNGE, complexes resistant to detergent are aligned in a diagonal easily identified by the Coomassie present in the running samples.
To prepare samples for 3rd dimension SDS-GE, the following solutions were used: RSa=1x LDS sample buffer (Life technologies), 4% β-mercaptoethanol; RSb=1x LDS sample buffer, 1% N, N-dimethylacrylamide (DMA, SIGMA) and RSc=1x LDS sample buffer, 20% ethanol and 0.1% β-mercaptoethanol.

BNGE of heart samples was performed in presence of dark cathode at 100 V for 3.5 hrs and bright cathode buffer at 150 V overnight.

Biochemistry

Protein lysis was performed in presence of proteases inhibitor cocktail (PIC, Sigma) for 30 min at 4°C. After centrifugation at maximal speed for 20 min, supernatants were collected and protein concentration was determined using Bradford reagent (Bio-Rad).

The following antibodies were used: anti-MIC60 polyclonal (1:1000, Proteintech), anti-OPA1 monoclonal (1:1000, BD PharMingen), anti-Fp (Flavoprotein subunit of succinate dehydrogenase, complex-II) monoclonal (1:4000, Abcam), anti-MIC19 polyclonal (1:1000, Proteintech), anti-TOM20 (1:2000, Santa Cruz) and anti-ACTIN (1:10000, Millipore) antibodies. Isotype matched secondary antibodies were conjugated to horseradish peroxidase and chemiluminescence was detected with ECL (Amersham) using either traditional methods with films (Amersham) or ImageQuant LAS 4000 (GE Healthcare Life Sciences).

Chemical crosslinking with dithiobis(succinimidyl propionate) (DSP, Pierce) was performed by incubating isolated mitochondria (1 mg/ml) with 2 mM DSP in EB for 30 min. Reaction was quenched with 20 mM Tris-HCl. Following quenching of the BMH or DSP crosslinking reaction, mitochondria were pelleted by centrifugation at 12000 x g, 10 min, 4°C. Pellet were solubilized in Laemmli buffer (LB) 2X, boiled and electrophoretically separated using a 3%-8% Tris-Acetate gel (Life technologies).

Immunoprecipitation

Isolated mitochondria from liver were lysed in 150 mM NaCl, 25 mM Tris-Cl pH 7.4, 1 mM EDTA, 5% glycerol, 0.1% Triton X-100 in the presence of PIC (Sigma). Lysates were precleared with 20 µl of Protein-A agarose beads (Roche) for 30 min at 4°C to avoid unspecific binding. Protein-A agarose beads (30 µl) were incubated with 2.4 µg anti-MIC60 in lysis buffer for 2 hr at 4°C. Subsequently, mitochondrial lysates (250 µg) were incubated with the antibody-coupled beads for 2 hr at room temperature. The immunoprecipitated material was washed once with 250 mM NaCl, 25 mM Tris-Cl pH 7.4, 1 mM EDTA, 0.05% Triton X-100. Bound proteins were eluted in LB 2X with 5% β-mercaptoethanol and 1% SDS, boiled and separated electrophoretically in a Bis-Tris 4%-12% gel (Life technologies).

Isolated mitochondria from mouse embryonic fibroblasts were lysed as described above. Beads were incubated with anti-OPA1 in lysis buffer (2 µg) and after 1 h the mitochondrial lysates (125 µg) were incubated with the pretreated beads for 3 hr. After washing of beads, bound proteins were eluted in LB 2X with 5% β-mercaptoethanol, and after boiling samples were separated electrophoretically in a Bis-Tris 4%-12% gel (Life technologies) and immunoblotted as indicated in figure legend.

For immunoprecipitation after chemical crosslinking with DSP, crosslinked mitochondria were lysed in 150 mM NaCl, 20 mM HEPES/KOH pH 7.4, 1 mM EDTA, 5% glycerol, 0.1% Triton X-100 and centrifuged at 21000 x g for 30 min. Part of the lysates were boiled in LB 1X containing when indicated 5% β-mercaptoethanol and 25 mM DTT to cleave the DSP linker. For immunoprecipitation, beads were incubated with anti-MIC60 in lysis buffer (2.4 µg) and after 1 hr the crosslinked mitochondria lysates (125 µg) were incubated with the pretreated beads for 3 hrs. The boiled samples were separated electrophoretically in a Tris-Acetate 3%-8% gel.

BNGE based semi-quantitative proteomic analysis

For mass spectrometry analysis of mitochondrial heart complexes, complexes solubilized from mitochondria purified from two mouse CD1 hearts as described above were separated by BNGE using a homemade 20x20 cm, 3%-13% acrylamide gel (Schagger, 1995). Three different experiments were loaded in parallel for each experimental condition. Gels were stained using GelCode Blue Stain (Thermo Scientific) and each lane was divided and excised in 25 bands covering the whole electrophoretic run. The same bands from the triplicate samples were pooled in an Eppendorf tube, minced and digested with modified porcine trypsin (Promega) at a final trypsin-protein 1:20 ratio overnight at 37°C in 100 mM ammonium bicarbonate, pH 8.8. The resulting tryptic peptide mixtures were subjected to nano-liquid chromatography coupled to mass spectrometry using a data-independent scanning (DiS) method as described (Guaras et al., 2016). Peptides were injected onto a C-18 reversed phase (RP) nano-column (75 µm I.D. and 50 cm, Acclaim PepMap100, Thermo Scientific) and analyzed in a continuous acetonitrile gradient consisting of 0%-30% B in 240 min, 50-90% B in 3 min (B=90% acetonitrile, 0.5% acetic acid). A flow rate of ca. 200 nL/min was used to elute peptides from the RP nano-column to an emitter nanospray needle for real time ionization and peptide fragmentation on an Orbitrap Fusion mass spectrometer (Thermo Fisher, San José, CA, USA). Protein identification was performed using Sequest running under Proteome Discoverer 1.4.0.288 (Thermo Fisher Scientific), allowing two missed cleavages, and using 3 Da and 20 ppm precursor and fragment mass tolerances, respectively. False discovery rate (FDR) of identification was controlled as described by the novel algorithm Dxtractor (Guaras et al., 2016; Cogliati et al., 2016).

SILAC labeling and proteomics

For SILAC labeling, in experiment "a" light mitochondria were treated with 20 pmol/mg cBID in EB for 30 min, 25°C, and heavy mitochondria were untreated. After centrifugation at 10000 x g, 10 min, 4°C, the pellets from both samples were mixed and resuspended together in NB to extract the complexes. In experiment "b", light mitochondria were untreated while heavy mitochondria were treated with 20 pmol/mg cBID^{KKAA} in EB for 30 min, 25°C. After centrifugation the pellets were mixed and processed as in experiment "a".

Protein complexes from experiments “a” and “b” were loaded onto parallel lanes and complexes were separated by BNGE. Three lanes from experiment “a” and “b” were stained with Coomassie and bands between 500 and 1000 kDa were excised with a scalpel and digested with trypsin. Digestion proceeded overnight at 37°C in 100 mM ammonium bicarbonate, pH 8.8. The resulting tryptic peptide mixtures were subjected to nano-liquid chromatography coupled to tandem mass spectrometry (LC-MS) for protein identification. Peptides were injected onto a C-18 reversed phase (RP) nano-column (75 µm I.D. and 50 cm, Acclaim PepMap100, Thermo Scientific) and analyzed in a continuous acetonitrile gradient consisting of 0-30% B in 240 min, 50-90% B in 3 min (B=90% acetonitrile, 0.5% acetic acid). A flow rate of ca. 200 nL/min was used to elute peptides from the RP nano-column to an emitter nanospray needle for real time ionization and peptide fragmentation on a Q Exactive mass spectrometer (Thermo Fisher, San José, CA, USA). Protein identification was performed using Sequest running under Proteome Discoverer 1.4.0.288 (Thermo Fisher Scientific). Peptide quantification was performed as described (Lopez-Ferrer et al., 2006; Ramos-Fernandez et al., 2007; Bonzon-Kulichenko et al., 2011), using QuiXoT. Proteins identified with only one peptide or peptides identified with only one PSM were removed from the data. Isotopic envelope of ion parents produced by the superimposition of unlabeled and singly labeled peptides was determined by least-squares fitting to spectrum peaks, bycalculating isotopic distributions from the peptide mass as described previously (Lopez-Ferrer et al., 2005). Statistical analysis took into account four different sources of variance at the spectrum-fitting, scan, peptide, and protein levels (Jorge et al., 2009).

Virus production and infection

The retroviral expression vector pMSCV-IRES-GFP containing truncated Bid (pMIG-tBid) was used to generate amphotropic retroviruses expressing tBID as described previously (Cogliati et al., 2013). The efficiency of transduction was typically around 80%-90% as determined by counting GFP positive cells with an epifluorescence microscope (data not shown). Experiments were performed 48 hrs post-infection on sorted GFP–positive cells.

Apoptosis detection

For cell death detection, 4 x 10^5 *Opa1*$^{flx/flx}$ MAFs were grown in 12-well plates, transfected with Mic60 siRNA and after. 48 hr treated with 2 µM Staurosporine (STS). After 6 hr cells were harvested and stained with Annexin-V-Alexa 568 (eBiosciences). Cell death was measured by flow cytometry (FACSCalibur, BD Biosciences) as the percentage of the Annexin-V-positive events in the total population.

Imaging

For imaging of mitochondrial network, cells (3.5 x 10^5) were seeded on 13 mm round glass coverslips, transfected as indicated and after 48 hr incubated with 5 nM Tetramethylrhodamine methyl ester (TMRM) in presence of 2 µM Cyclosporine H (CsH) in Hank’s Balanced Salt Solution (HBSS) supplemented with 10 mM Hepes. Fluorescence signals were analyzed using IMIC Andromeda system (Fondis Electronic) equipped with ORCA-03G Camera (Hamamatsu), a 60X oil objective (UPLAN 60X, 1.35NA, Olympus), a 561 nm laser for excitation and a FF01-446/523/600/677 emission filter (Semrock). Measurement of mitochondrial major axis length was performed in at least six mitochondria per cell, in a minimum of 30 cells/experiment (180 mitochondria/experiment). Mitochondrial length was then quantified using Image J tool ‘Freehand line selection’.
For imaging of mitochondrial membrane potential, 7 x 10^5 cells seeded on 24 mm round glass coverslips were incubated with 5 nM TMRM in presence of 2 µM CsH in HBSS supplemented with 10 mM Hepes for 30 min at 37°C. TMRM fluorescence was followed in time by acquisition of sequential images every minute with an Olympus IMT-2 inverted microscope equipped with a CellR Imaging system. Where indicated Oligomycin (2µM) or Carbonyl cyanide-4-(trifluoromethoxy)phenylhydrazone (FCCP, 2 µM) were added.

Analysis of mitochondrial respiration

Oxygen consumption rate (OCR) was measured with the XF24 Extracellular Flux Analyzer (Seahorse Bioscience, Billerica, MA, USA). MAFs were seeded in XF24 cell culture microplates at 6 × 10^4 cells/well with complete DMEM. After 24 hrs cells were transfected with Mic60 siRNA (#1-3) or scramble oligos and maintained 36-48 hrs in 0.2 ml complete medium at 37°C in 5% CO_2. Experiments were carried out on confluent monolayers and the measurements were initiated after replacing the medium with Cellular Assay Solution (8.3 g/L DMEM, 5 g/L Glucose, 0.58 g/L Glutamine, 1 mM Sodium Pyruvate, 0.015 g/L Phenol Red, pH 7.2). Cells were incubated at 37°C for 30 min to allow temperature and pH equilibration. A titration with FCCP was performed to determine the optimal FCCP concentration. After three measurements of basal OCR each 5 min, 70 µl of a solution containing oligomycin, FCCP or rotenone plus antimycin A were sequentially added to each well to reach final concentrations of 1.3 µM oligomycin, 0.4 µM FCCP and 1 µM for rotenone and 1.2 µM antimycin A. Data are expressed as pmol of O_2 per minute. Three measurements were performed after each compound injection. The analysis was performed simultaneously in the same plate for the control and *Mic60* knockdown cells.

Electron Microscopy and Tomography

Cells were fixed and processed for electron microscopy analysis 48 hrs after transfection. When indicated, cell sorting (FACS Canto, BD Biosciences) was performed 24 hr post-infection to select GFP or/and RFP positive cells, which were subsequently seeded and fixed 48 hr post-infection or as specified. Cell fixation was performed for 1 hr at 37°C with freshly prepared 1.25% (V/V) glutaraldehyde in 0.1 M sodium cacodylate, pH 7.4. After washing with 0.1 M sodium cacodylate, cells were post-fixed in 1% OsO_4, 1.5% $K_4Fe(CN)_6$ in 0.1 M sodium cacodylate pH 7.4, stained with 0.5% uranyl acetate, dehydrated in ethanol and embedded in Embed 812. Thin sections were imaged on a Tecnai-12 electron microscope (Philips-FEI) at the EM Facility of University of Padua.
Electron tomography was performed as previously described (Frezza et al., 2006) on 200-250 nm thick sections collected on Formvar-coated copper slot grids with gold fiducials (10 nm) applied on both surfaces of the grids imaged in a 200 kV Tecnai G2 20 electron microscope (FEI, Eindhoven, The Netherlands) at magnification of 25k or 29k resulting in a pixel's size of 0.92 nm and 0.77 nm. Tilted images (+65/-65 according to a Saxton scheme with a starting angle of 1.5°) were acquired using Xplorer 3D (FEI, Eindhoven, The Netherlands) with an Eagle 2k×2k CCD camera (FEI, Eindhoven, The Netherlands). Tilted series alignment and tomography reconstruction was performed using IMOD (Mastronarde, 1997) and segmentation and 3D rendering using Reconstruct (Fiala, 2005). Measurements were performed using ImageJ and the z scale was stretched using a 1.6 factor to correct for resin shrinkage (Mastronarde, 1997).
Number of frames reconstructed in the tomograms of Figure 5 were as following: EV- scr/EV: 72f; EV- Mic60 siRNA: 98f; EV-(+MIC60): 62f; CRE- scr/EV: 98f; CRE- Mic60 siRNA: 120f; CRE-(+MIC60): 108f; *Opa1*^{tg}-scr/EV: 98f; *Opa1*^{tg}-Mic60 siRNA:110f; *Opa1*^{tg}-(+MIC60):110f.

Supplemental References

Bonzon-Kulichenko, E., Pérez-Hernández, D., Núñez, E., Martínez-Acedo, P., Navarro, P., Trevisan-Herraz, M., Ramos Mdel, C., Sierra, S., Martínez-Martínez, S., Ruiz-Meana, M., Miró-Casas, E., García-Dorado, D., Redondo, J.M., Burgos, J.S., Vázquez, J. (2011). A robust method for quantitative high-throughput analysis of proteomes by 18O labeling. Mol Cell Proteomics *10*, M110.003335.

Cogliati, S., Calvo, E., Loureiro, M., Guaras, A.M., Nieto-Arellano, R., Garcia-Poyatos, C., Ezkurdia, I., Mercader, N., Vázquez, J., Enriquez, J.A. (2016). Mechanism of super-assembly ofrespiratory complexes III and IV. Nature. DOI: 10.1038/nature20157.

Fiala, J.C. (2005). Reconstruct, a free editor for serial section microscopy. J Microsc. *218*, 52-61.

Guarás, A., Perales-Clemente, E., Calvo, E., Acín-Pérez, R., Loureiro-Lopez, M., Pujol, C., Martínez-Carrascoso, I., Nuñez, E., García-Marqués, F., Rodríguez-Hernández, M.A., *et al*. (2016). The CoQH2/CoQ ratio serves as a sensor of respiratory chain efficiency. Cell Rep. *15*, 197-209.

Jorge, I., Navarro, P., Martínez-Acedo, P., Núñez, E., Serrano, H., Alfranca, A., Redondo, J.M., Vázquez, J. (2009). Statistical model to analyze quantitative proteomics data obtained by 18O/16O labeling and linear ion trap mass spectrometry, application to the study of vascular endothelial growth factor-induced angiogenesis in endothelial cells. Mol Cell Proteomics *8*, 1130-49.

López-Ferrer, D., Capelo, J.L., Vázquez, J. (2005). Ultra fast trypsin digestion of proteins by high intensity focused ultrasound. J Proteome Res. *4*, 1569-74.

López-Ferrer, D., Ramos-Fernández, A., Martínez-Bartolomé, S., García-Ruiz, P., Vázquez, J. (2006). Quantitative proteomics using 16O/18O labeling and linear ion trap mass spectrometry. Proteomics *6*, S4-11.

Mastronarde, DN. (1997). Dual-axis tomography, an approach with alignment methods that preserve resolution. J Struct Biol. *120*, 343-52.

Ramos-Fernández, A., López-Ferrer, D., Vázquez, J. (2007). Improved method for differential expression proteomics using trypsin-catalyzed 18O labeling with a correction for labeling efficiency. Mol Cell Proteomics *6*, 1274-86.

Schägger, H. (1995). Quantification of oxidative phosphorylation enzymes after blue native electrophoresis and two-dimensional resolution, normal complex I protein amounts in Parkinson's disease conflict with reduced catalytic activities. Electrophoresis *16*, 763-70.
